# Supplementary material for: COmapper: high‐resolution mapping of meiotic crossovers by long‐read sequencing in Arabidopsis
Source: New Phytol. 2025 Jun 19;247(4):1942–57. doi: 10.1111/nph.70304 (PMC12267935; doi:10.1111/nph.70304)
Supplement: Supplementary file 3 — Fig. S1 Distribution of read length from long‐read nanopore sequencing. Fig. S2 Nanopore sequencing read coverage. Fig. S3 Comparison of genome structure and single nucleotide polymorphisms between the Col and Ler accessions. Fig. S4 Validation of COmapper for mapping crossovers using Col × Ler F2 individuals. Fig. S5 Estimating false positive rates attributable to nanopore sequencing errors. Fig. S6 Representative simple crossover and complex crossover sites identified by COmapper in nanopore sequencing reads. Fig. S7 Representative complex crossover sites identified by both COmapper and GBS allele frequency analysis. Fig. S8 Sources of false positives in COmapper results from pooled 4 or 46 Col × Ler F2 individuals. Fig. S9 Analysis of crossovers not detected by COmapper from 46 Col × Ler F2 individuals. Fig. S10 Impact of sequencing coverage on COmapper performance. Fig. S11 Validation of COmapper using recq4ab Col × recq4ab Ler F2 recombinant plants. Fig. S12 Validation of compatibility between TAIR10 and Col‐CEN genomes. Fig. S13 Comparison of genome‐wide crossover landscapes from WT Col × Ler F1 pollen COmapper replicates. Fig. S14 Comparison of SNP distances at crossover sites mapped by GBS and COmapper. Fig. S15 Genomic region classification based on 8‐SNP length and corresponding crossover distributions. Fig. S16 Crossovers occur more frequently at gene promoters and less frequently at gene bodies than a random distribution. Fig. S17 Resolution of crossovers mapped by GBS and COmapper. Fig. S18 Class I crossovers are associated with higher SNP density. Fig. S19 Analysis of SNP density between Solanum lycopersicum and Solanum pimpinellifolium. Table S1 Summary of long‐read nanopore sequencing libraries used in this study. Table S2 Detailed information for the sequencing libraries used in this study. Table S3 Comparison of genome‐wide crossover mapping techniques. Please note: Wiley is not responsible for the content or functionality of any Supporting Informa [file NPH-247-1942-s003.pdf]

New Phytologist

New Phytologist Supporting Information

Article title: COmapper: High-resolution mapping of meiotic crossovers by long-read sequencing in *Arabidopsis*

Authors: Dohwan Byun, Namil Son, Heejin Kim, Jaeil Kim, Jihye Park, Sang-jun Park, Hyein Kim, Jaebeom Kim, Juhyun Kim, Seula Lee, Youbong Hyun, Piotr A. Ziolkowski, Ian R. Henderson, and Kyuha Choi

Article acceptance date: 02 June 2025

The following Supporting Information is available for this article:

Fig. S1 Distribution of read length from long-read nanopore sequencing.

Fig. S2 Nanopore sequencing read coverage.

Fig. S3 Comparison of genome structure and single-nucleotide polymorphisms between the Col and L*er* accessions.

Fig. S4 Validation of COmapper for mapping crossovers using Col × L*er* F_2 individuals.

Fig. S5 Estimating false positive rates attributable to nanopore sequencing errors.

Fig. S6 Representative simple crossover and complex crossover sites identified by COmapper in nanopore sequencing reads.

Fig. S7 Representative complex crossover sites identified by both COmapper and GBS allele frequency analysis.

Fig. S8 Sources of false positives in COmapper results from pooled 4 or 46 Col × L*er* F_2 individuals.

Fig. S9 Analysis of crossovers not detected by COmapper from 46 Col × L*er* F_2 individuals.

Fig. S10 Impact of sequencing coverage on COmapper performance.

Fig. S11 Validation of COmapper using recq4ab Col × *recq4ab* L*er* F_2 recombinant plants.

Fig. S12 Validation of Compatibility between TAIR10 and Col-CEN Genomes.

Fig. S13 Comparison of genome-wide crossover landscapes from WT Col × L*er* F_1 pollen COmapper replicates.

Fig. S14 Comparison of SNP distances at crossover sites mapped by GBS and COmapper.

Fig. S15 Genomic region classification based on 8-SNP length and corresponding crossover distributions.

Fig. S16 Crossovers occur more frequently at gene promoters and less frequently at gene bodies than a random distribution.

Fig. S17 Resolution of crossovers mapped by GBS and COmapper.

Fig. S18 Class I crossovers are associated with higher SNP density.

Fig. S19 Analysis of SNP density between Solanum *lycopersicum* and Solanum *pimpinellifolium.*

Table S1 Summary of long-read nanopore sequencing libraries used in this study.

Table S2 Detailed information for the sequencing libraries used in this study.

Table S3 Comparison of genome-wide crossover mapping techniques.

Dataset S1 Allele frequency maps of 46 and 4 F_2 individuals.

Dataset S2 Information of crossover sites in 46 and 4 F_2 individuals (a separate excel file).

Dataset S3 Screenshots of false positives.

Dataset S4 Fisher's exact test for Fig. S9c (a separate excel file).

References

Fig. S1 Distribution of read length from long-read nanopore sequencing.

Distribution of read length from nanopore sequencing of the samples used in this study. Vertical blue lines indicate the N50 read length.

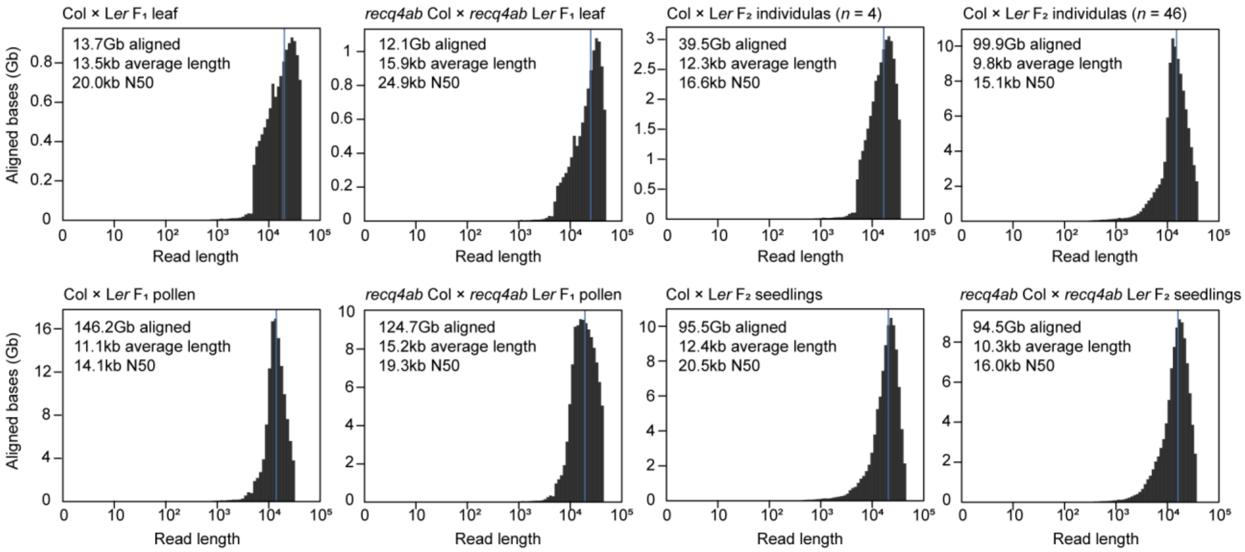

Fig. S2 Nanopore sequencing read coverage. Coverage frequencies of long-read nanopore sequencing depth in the number of reads along the five *Arabidopsis* chromosomes on a continuous *x*-axis in the samples used in this study. The vertical dashed lines indicate centromere assembly gap positions, and the vertical lines indicate the ends of the chromosomes. The pericentromeric regions are highlighted in blue.

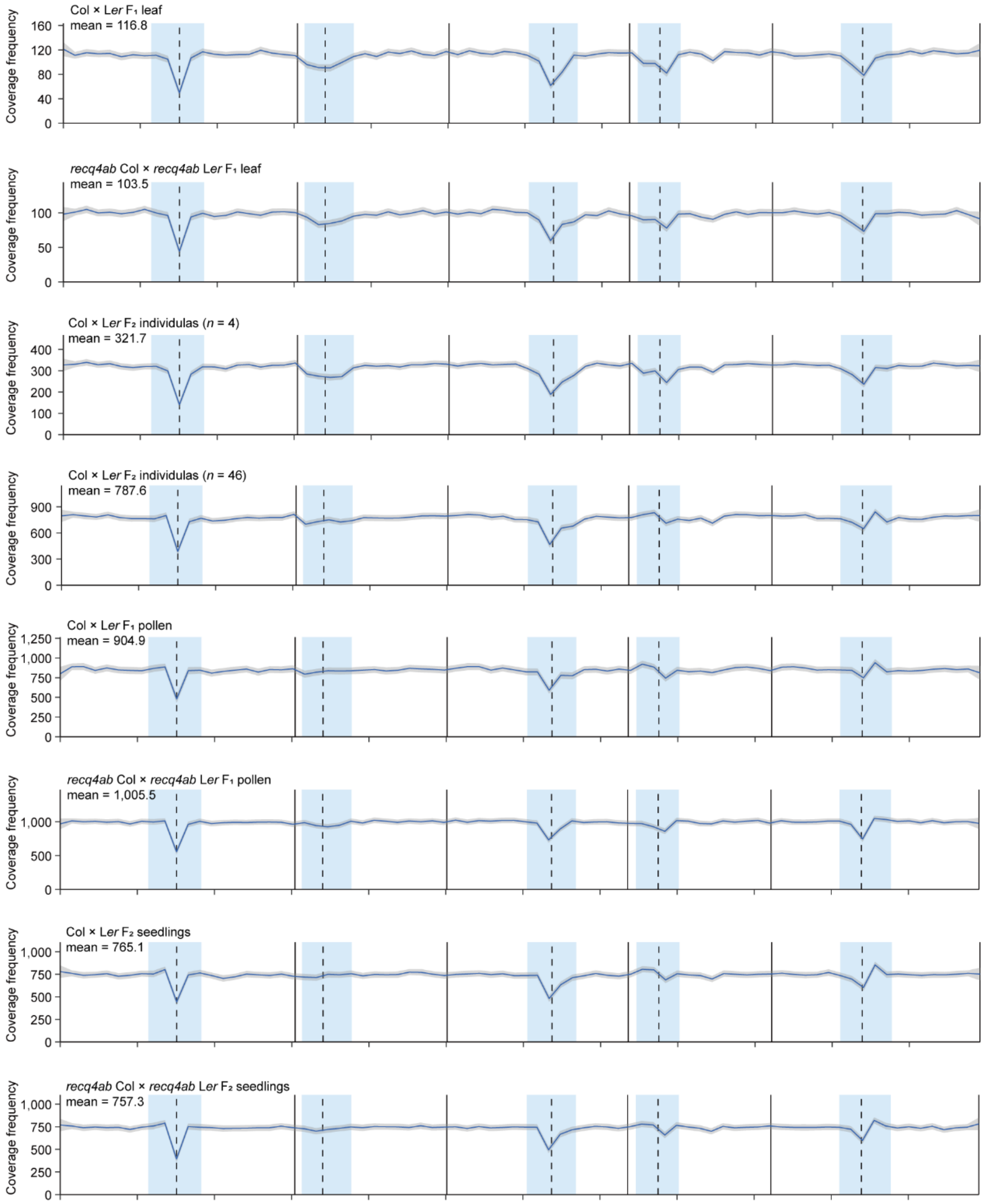

Fig. S3 Comparison of genome structure and single-nucleotide polymorphisms between the Col and L*er* accessions. SNP markers (black, SNPs/100 kb), syntenic (gray), translocation (green), duplication (sky blue), inversion (orange) and centromere gaps (black dot) are shown along the five chromosomes of *Arabidopsis* Col (red) and L*er* (blue) accessions.

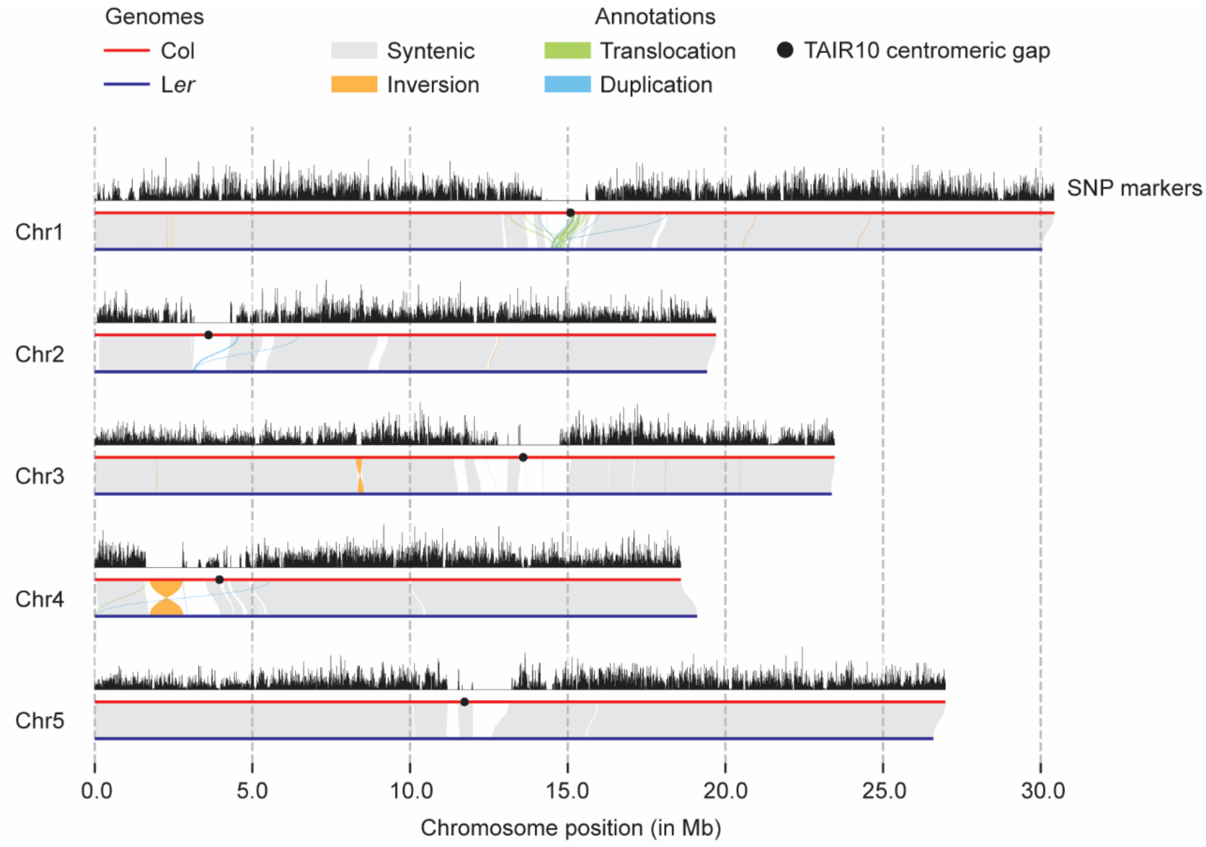

Fig. S4 Validation of COmapper for mapping crossovers using Col × L*er* F_2 individuals. (**a**) Diagram of the strategy used to map crossovers for each individual by individual barcoding, performing GBS, and running TIGER of genomic DNAs from 4 or 46 Col × L*er* F_2 or 39 *recq4ab* Col × *recq4ab* L*er* F_2 recombinant individuals. The same genomic DNA purified from each F_2 individuals were pooled to generate two separate libraries for nanopore sequencing. (**b**) Summary table of the amount of DNA and genome coverage from long-read nanopore sequencing, and the number of crossovers mapped by the GBS and COmapper approaches.

(a)

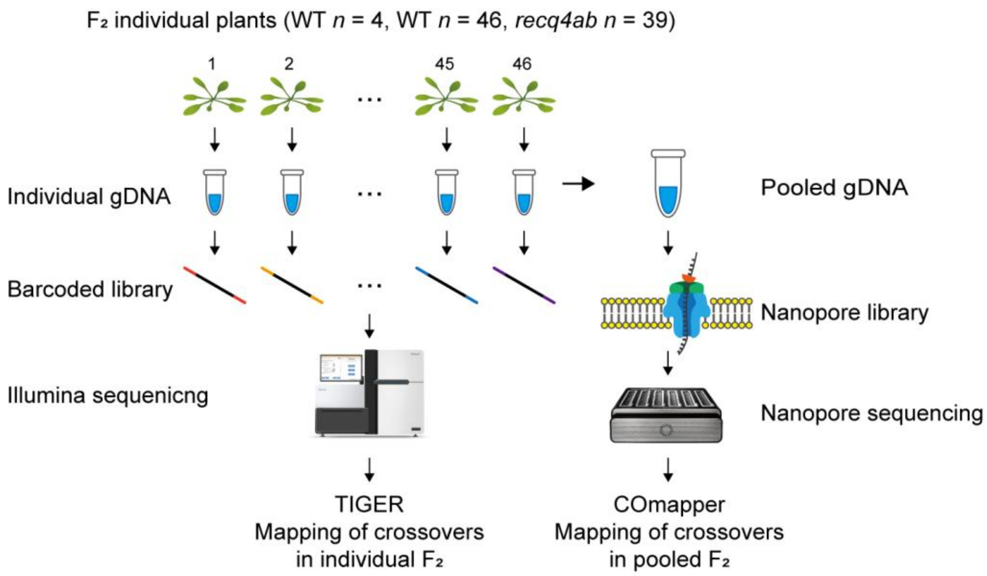

(b)

F_2 plants	Reference crossover sites	Nanopore sequenceing coverage (Gb)	Crossover reads mapped by COmapper	Reference sites mapped by COmapper
WT (*n* = 4)	33	40.1	630	33
WT (*n* = 46)	342	100.7	1659	299
recq4ab (*n* = 39)	1055	46.8	1448	532

Fig. S5 Estimating false positive rates attributable to nanopore sequencing errors. (**a**) Density plot showing the distribution of average base quality scores and sequence identity of nanopore reads, used to estimate average sequencing error rate. (**b**) Schematic illustration of how consecutive sequencing errors can result in false positive crossovers. The probability of each error was calculated based on a Q14 error rate, assuming that each of the three alternative bases has an equal probability of being erroneously called. The cumulative probability of such errors in the 4-SCO model was estimated to be 0.0357%. (**c**) Table showing the expected and observed numbers of false positive crossovers per million reads across different COmapper models. (**d**) Examples of a false positive crossover identified in the 4-SCO model. Col alleles are represented as ‘C’, L*er* alleles as ‘L’, and undetermined alleles as ‘N’; ‘N’ positions were excluded from the COmapper analysis.

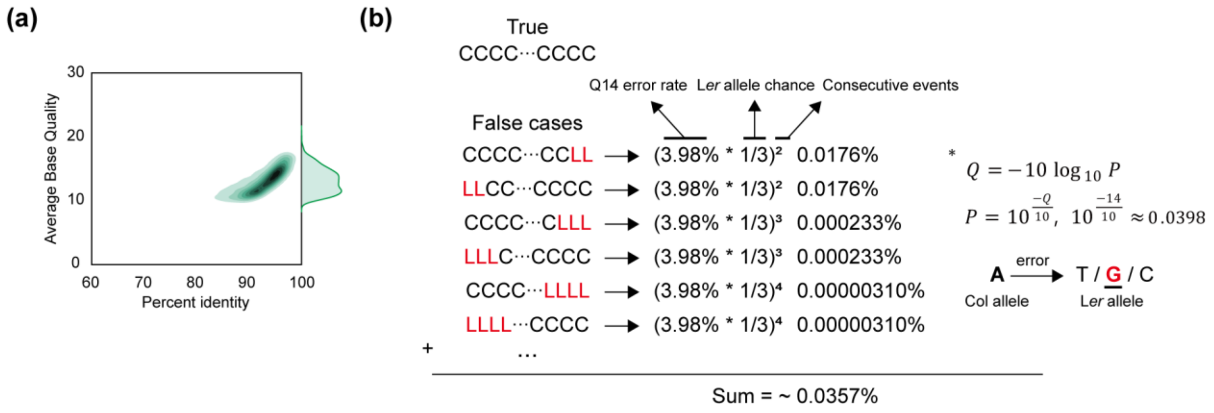

(c)

	4-SCO	6-SCO	8-SCO	8-CO
Expected	357	5	0.06	0.12
Observed	496	59	3.7	3.9

(False positives per million reads)

(d)

	Position	SNP genotypes
read1	Chr4::9,264,958-9,286,306	LNNLLLNLLLNLLLLLLLLNLLLCC
read2	Chr4::16,468,045-16,486,419	NCCNLLNLLNLLLLLLLLNN
read3	Chr5::18,014,967-18,029,258	CCCNCCCCNCCCCCCLL
read4	Chr1::17,232,360-17,240,240	LLNCCCCCCCCCC

Fig. S6 Representative simple crossover and complex crossover sites identified by COmapper in nanopore sequencing reads. (**a**) Plots showing the interval of three independent crossovers with a simple exchange between consecutive specific SNPs mapped by COmapper in five nanopore sequencing reads. Red dots indicate Col-specific SNPs, blue dots indicate L*er*-specific SNPs and gray dots indicate missing SNP information due to sequencing errors. Pink areas indicate Col haplotype regions and sky-blue areas indicate L*er* haplotype regions. (**b**) As in (**a**), but showing complex crossover sites.

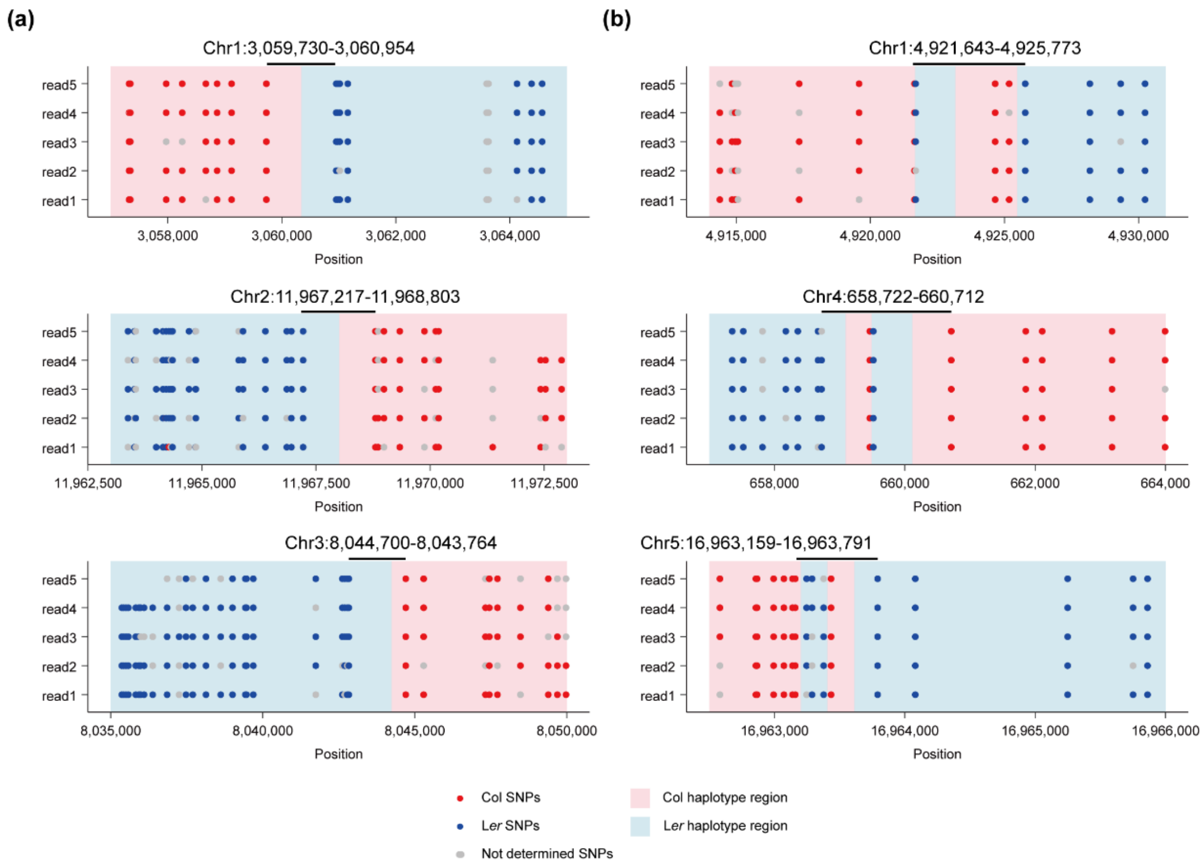

Fig. S7 Representative complex crossover sites identified by both COmapper and GBS allele frequency analysis. Plots showing three examples of complex crossover regions. The upper panel displays SNP mapping results from COmapper, while the lower panel shows allele frequency data from GBS. In the upper panel, red dots indicate Col-specific SNPs, blue dots indicate L*er*-specific SNPs, and gray dots indicate missing SNP information due to sequencing errors. In the lower panel, black dots show the L*er* allele frequency based on GBS data, pink areas indicate Col/Col diplotype regions, sky-blue areas indicate Col/L*er* diplotype regions, and yellow triangles point to GBS SNP sites with sequencing depth below 2.

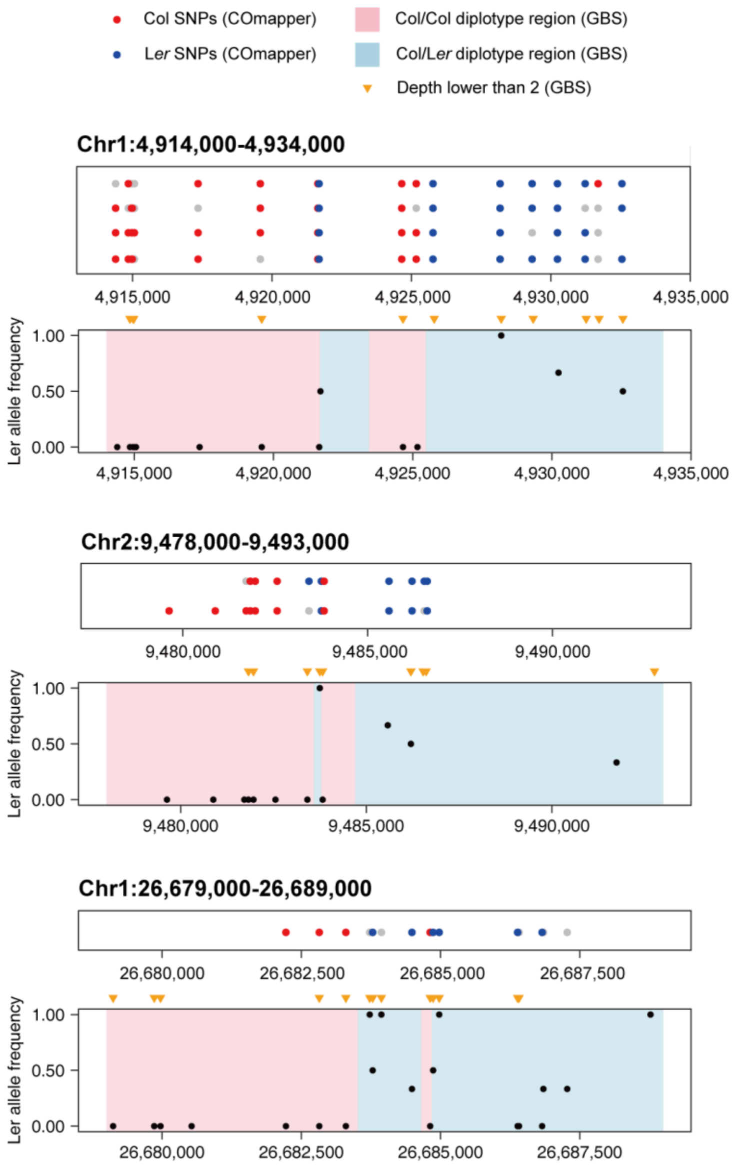

Fig. S8 Sources of false positives in COmapper results from pooled 4 or 46 Col × L*er* F2 individuals. The bar plot illustrates the number of different sources of false positive crossovers identified in WT Col × L*er* F2 individuals. (*n* = 4 and *n* = 46). The categories of sources are color-coded as follows: chimeric reads with error clusters (dark blue), substitution errors (orange), mitotic recombination artifacts (green), and chimeric reads with large indels (purple). The total number of false positives observed is indicated above the respective bar.

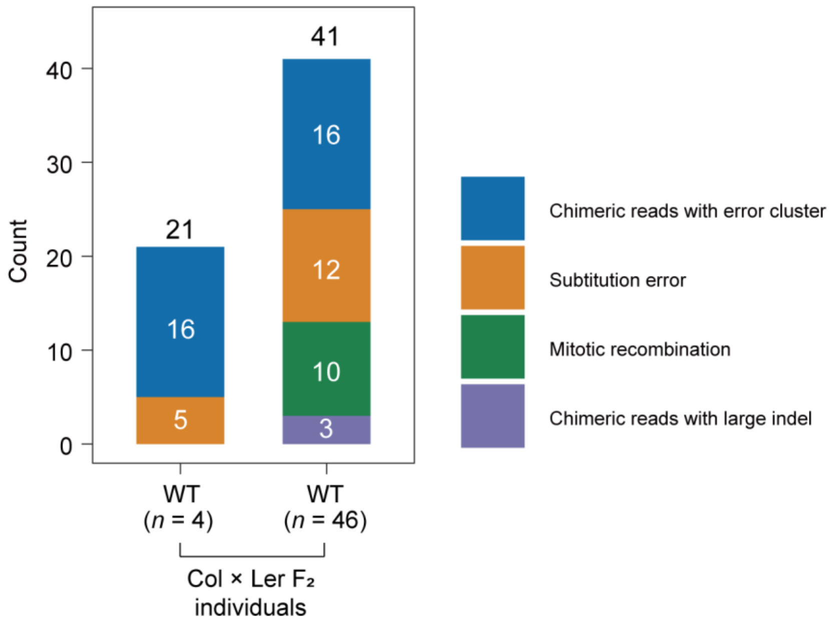

Fig. S9 Analysis of crossovers not detected by COmapper from 46 Col × L*er* F_2 individuals. (**a**) Number of crossovers detected and undetected by COmapper in the 46-individual pool, categorized by the length of 8 SNPs flanking the crossover sites. (**b**) Proportion of detected and undetected crossovers across the same 8-SNP length categories as in (a). (**c**) Genomic distribution of detected (blue) and undetected (red) crossovers along the five chromosomes (TAIR10 reference genome). Vertical dotted lines mark the centromeric gaps; grey regions indicate pericentromeric regions.

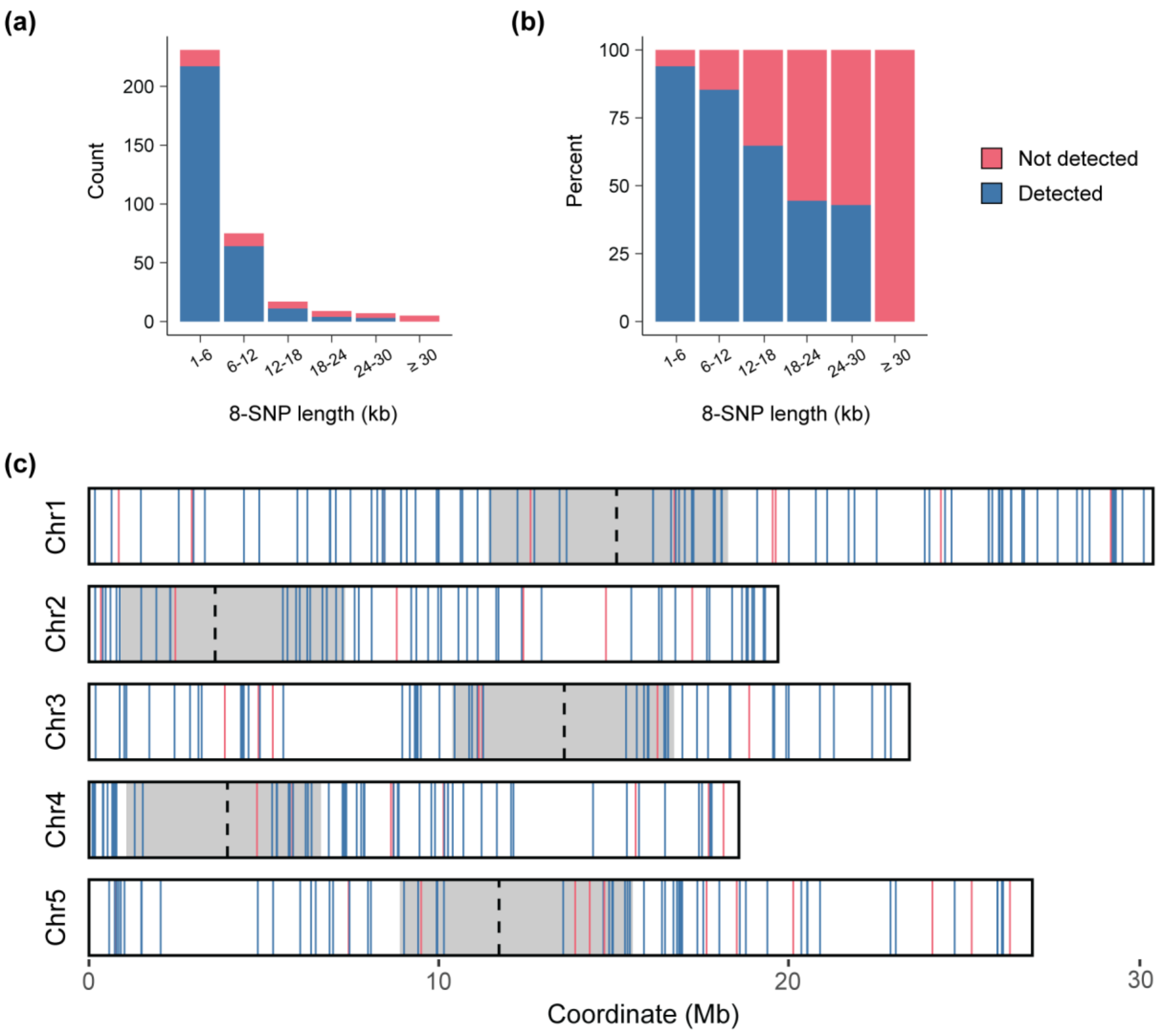

Fig. S10 Impact of sequencing coverage on COmapper performance. (**a**-**c**) True positive (**a**), false positive (**b**), and precision (TP/[TP + FP]) (**c**) as a function of sequencing coverage, based on subsampling of data from for 4 F_2 individuals. (**d**-**f**) Same as (**a-c**), but based on subsampling of data from 46 F_2 individuals. (**g**) Number of undetected crossovers (false negatives), plotted as a function of coverage per F_2. (**h**) The proportion of detected crossovers relative to total crossovers in the 4 F_2 dataset, plotted as a function of sequencing coverage per F_2. Vertical lines represent coverage per F_2 for 4 (blue), 46 (red) F_2 individuals, and 1,000 (yellow) F_2 seedlings. (**i**) Proportion of non-overlapping crossover sites per total detected crossovers, as a function of sequencing coverage per F_2. Vertical lines indicate coverage per F_2 for 4 (blue), 46 (red) F_2 individuals, and 1,000 (yellow) F_2 seedlings.

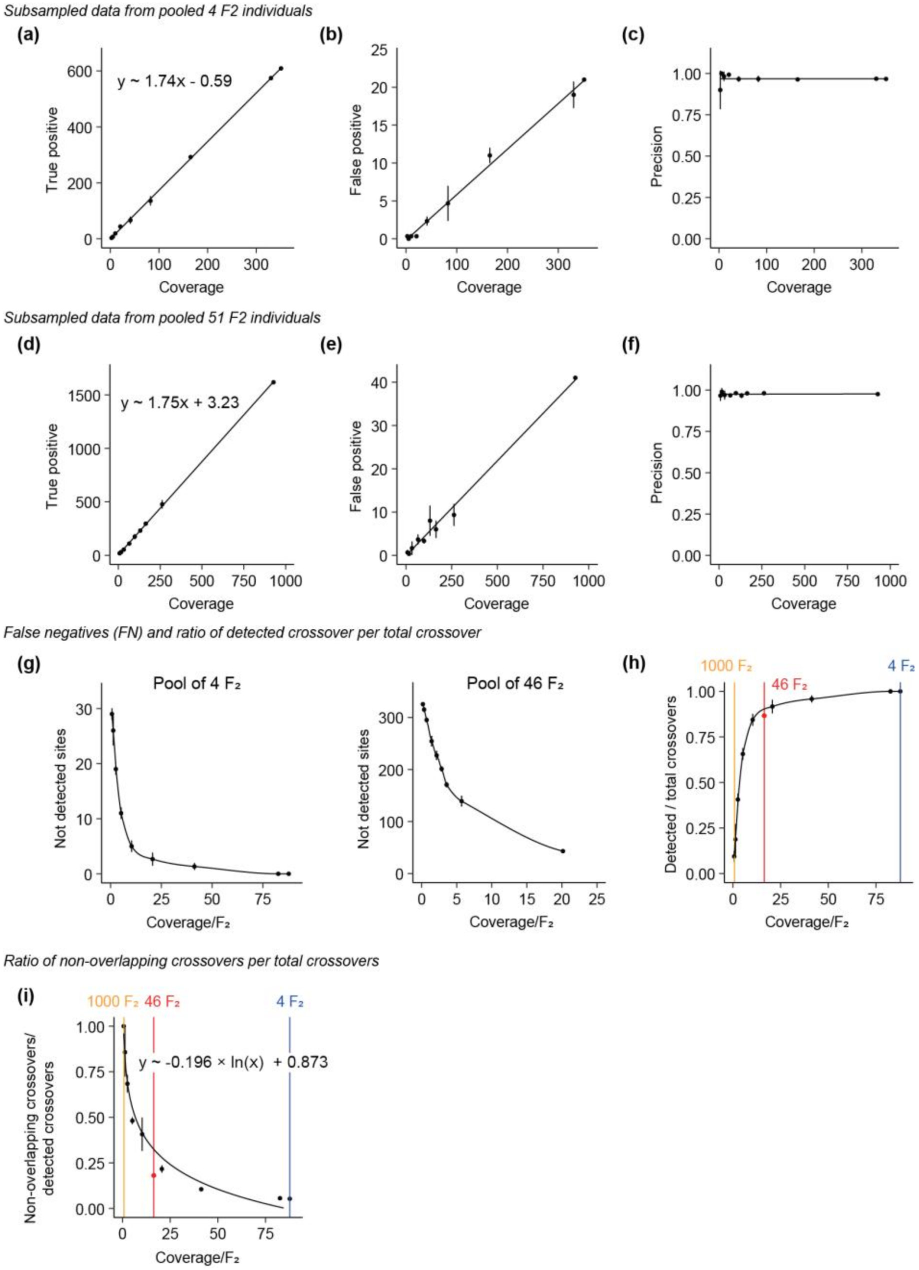

Fig. S11 Validation of COmapper using recq4ab Col × *recq4ab* Ler F_2 recombinant plants. (**a**) Number of crossovers detected per million reads in different COmapper models. True crossovers (blue) were detected by COmapper models among the crossovers mapped by GBS in 39 *recq4ab* Col × *recq4ab* Ler F_2 plants, and false crossovers (red) indicate the crossovers that differ from the crossovers defined by GBS. (**b**) Precision of crossover detection in the different COmapper models, include Col × L*er* F_2 plants shown in Fig. **3e**. (**c**) Precision of 8-CO COmapper model in WT and *recq4ab* F_2 individuals, respectively done by random subsampling. (**d**) Crossover per haploid in Col-0 × L*er* F_2 individuals and *recq4ab* Col × *recq4ab* Ler F_2 individuals. (**e**) Relative proportion of crossover types of *recq4ab* Col × *recq4ab* Ler F_2 individuals with Col × L*er* F_2 individuals, F_1 pollen, and F_2 seedlings data shown in Fig. **3f**. (**f**) Table of crossover numbers, coverage, and precision of Col × L*er* F_2 individuals (*n* = 4 and *n* = 46) and *recq4ab* Col × *recq4ab* Ler F_2 individuals (*n* = 39). (**g**) Categorization of false positive sources in *recq4ab* Col × *recq4ab* Ler F_2 individuals with Col × L*er* F_2 false positive sources data shown in Fig. **S8**.

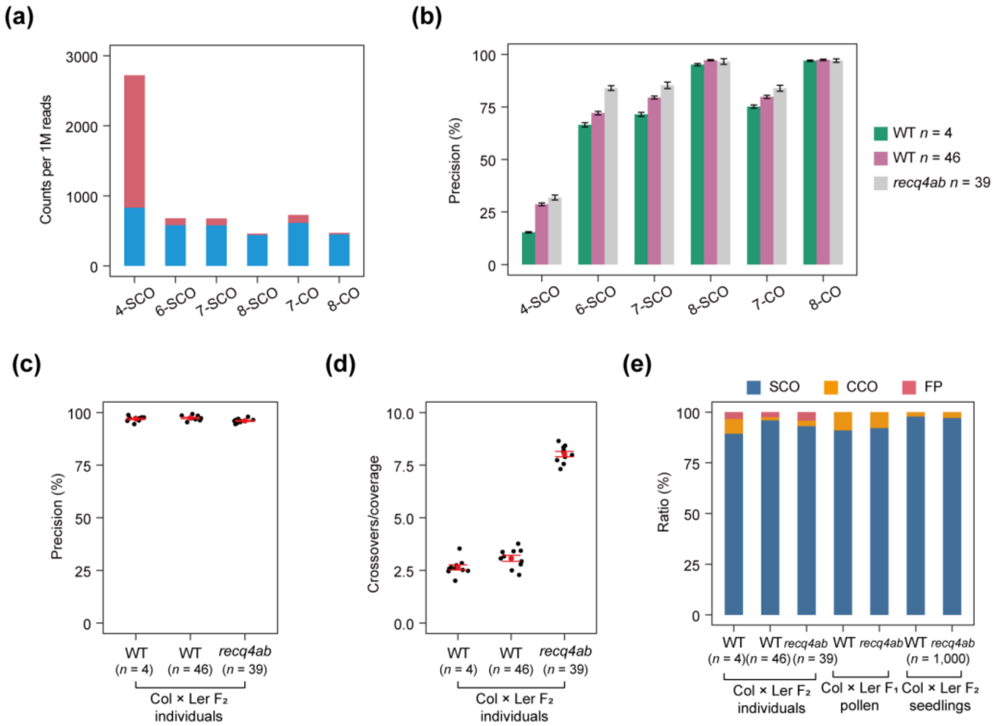

(f)

	n	Reference crossovers (GBS)	Coverage (COmapper)	True positive	False positive	Not detected	Precision (%)
WT F₂	4	33	337	609	21	0	96.7
WT F₂	46	342	845	1618	41	45	97.5
recq4 F₂	39	1055	410	1387	64	523	95.6

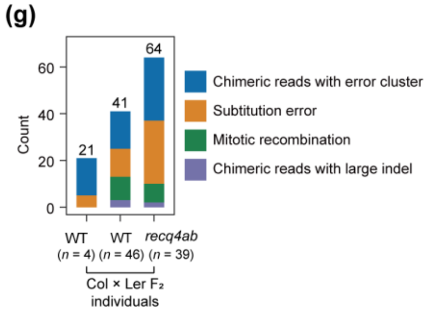

Fig. S12 Validation of Compatibility between TAIR10 and Col-CEN Genomes. (**a**) Procedure for TAIR10 SNP converting to Col-CEN using Liftoff pipeline. (**b**) A Venn diagram illustrating the overlap of crossover reads mapped by TAIR10 and Col-CEN reference genome within a set of 46 Col-0 × L*er* F_2 individuals. The overlapping region (purple) represents 1,658 SNPs that were consistently identified and mapped to both reference genomes. The non-overlapping regions (light red and light blue) indicate SNPs uniquely mapped to either TAIR10 or Col-CEN.

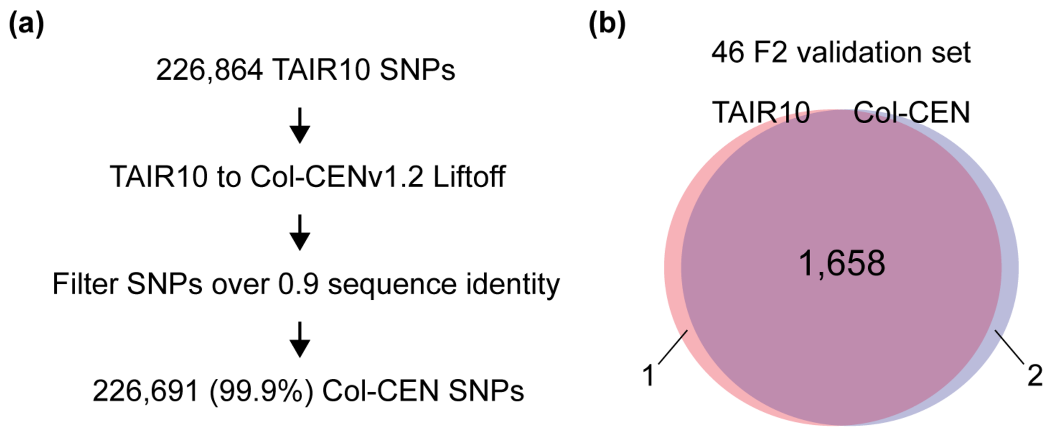

Fig. S13 Comparison of genome-wide crossover landscapes from WT Col × L*er* F_1 pollen COmapper replicates. (**a**) Genome-wide crossover landscapes obtained from two independent WT Col × L*er* F_1 pollen COmapper experiments. Relative crossover (CO) rates were calculated in non-overlapping 100-kb bins by dividing the number of crossovers per bin by the total number of crossovers, followed by smoothing using moving average. (**b**) As in (**a**), but comparing crossover landscape from GBS of WT Col × L*er* F_1 male meiosis with WT Col × L*er* F_1 pollen COmapper replicate 1. (**c**) As in (**a**), but comparing crossover landscape from GBS of WT Col × L*er* F_1 male meiosis with WT Col × L*er* F_1 pollen COmapper set 2. Fisher′s exact test was applied to each 100-kb bin to assess differences in relative crossover rates, with P-values corrected using Benjamini-Hochberg method. Bins with false discovery rate (FDR) < 0.05 are highlighted in yellow.

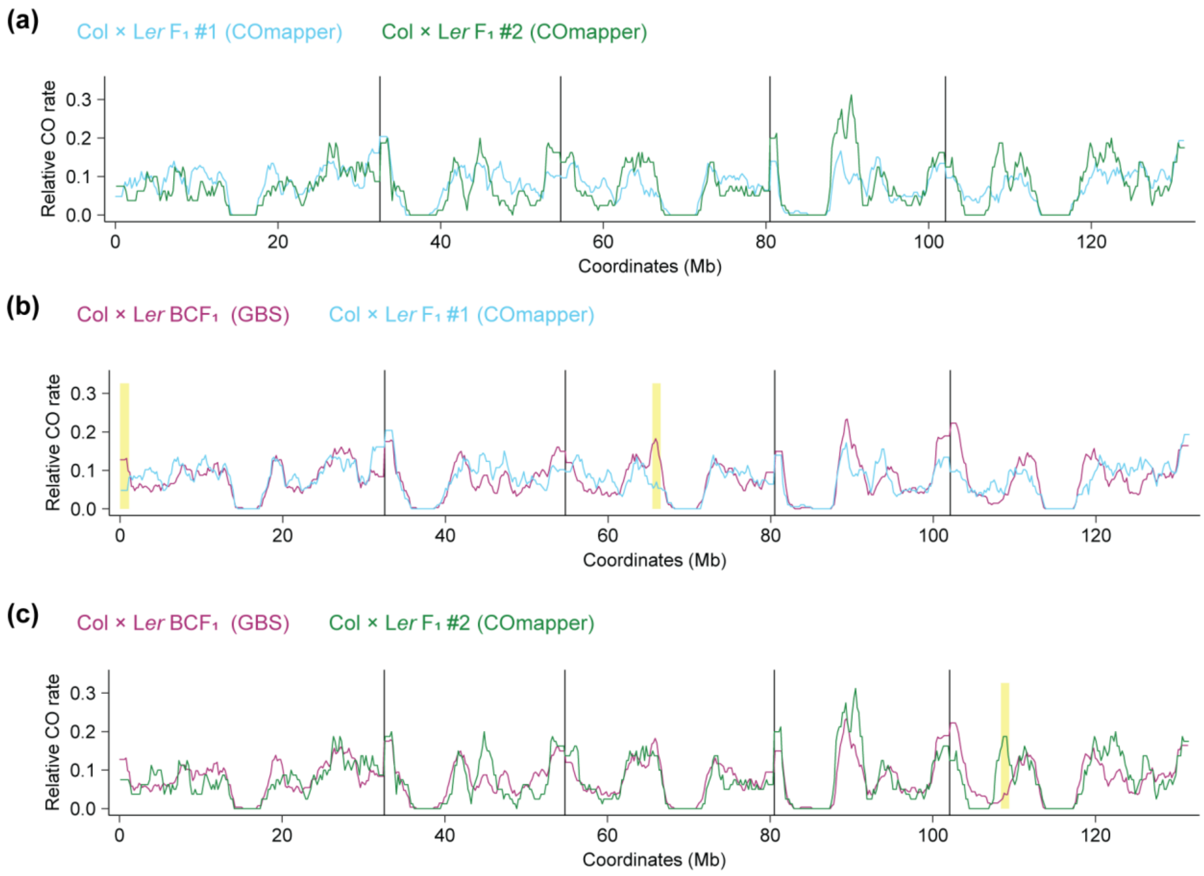

Fig. S14 Comparison of SNP distances at crossover sites mapped by GBS and COmapper. (**a**) Relative crossovers mapped by GBS (blue, 1,824 crossovers) and COmapper (red, 1,679 crossovers) along the chromosome telomere (TEL)-to-centromere (CEN) axis. (**b**) Summary of the physical coordinates of sub-telomeric, mid-chromosomal, pericentromeric, and centromeric regions for each chromosome. (**c**) SNP density in sub-telomeric, mid-chromosomal and pericentromeric regions along chromosomes. Vertical dashed lines indicate the median number of SNPs per 10-kb window. Asterisks indicate significant differences between regions (*P < 0.05, **P < 0.01, ***P < 0.001; Wilcoxon test). (**d**) Histogram showing the length of eight-SNP strings at crossovers mapped by GBS (blue, Col × L*er* BCF_1) and COmapper (red, Col × L*er* F_1 pollen) for male meiosis in sub-telomeric regions. Vertical dashed lines indicate the median length (in bp) of eight-SNP strings. Asterisks indicate significant differences between genotypes (*P < 0.05, **P < 0.01, ***P < 0.001; Wilcoxon test). (**e**) As in (**d**), but showing the mid-chromosomal regions. (**f**) As in (**d**), but showing the pericentromeric regions.

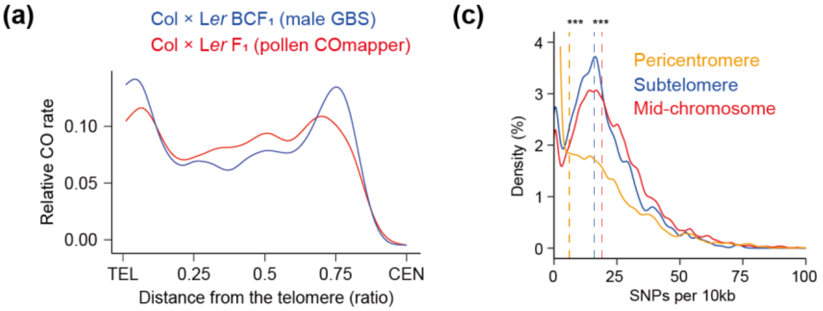

(b)

Chr.	Subtelomere (northern arm)	Mid-chromosome (northern arm)	Pericentromere	Mid-chromosome (southern arm)	Subtelomere (southern arm)
1	1~1,508,604	1,508,605~ 11,420,000	11,420,001~ 18,270,000	18,270,001~ 28,893,508	28,893,509~ 30,427,671
2	1~910,000		910,001~ 7,320,000	7,320,001~ 18,089,253	18,089,254~ 19,698,289
3	1~1,358,778	1,358,779~ 10,390,000	10,390,001~ 16,730,000	16,730,001~ 22,472,625	22,472,626~ 23,459,830
4	1~1,070,000		1,070,001~ 6,630,000	6,630,001~ 17,122,152	17,122,153~ 18,585,056
5	1~1,172,502	1,172,503~ 8,890,000	8,890,001~ 15,550,000	15,550,001~ 25,450,454	25,450,455~ 26,975,502

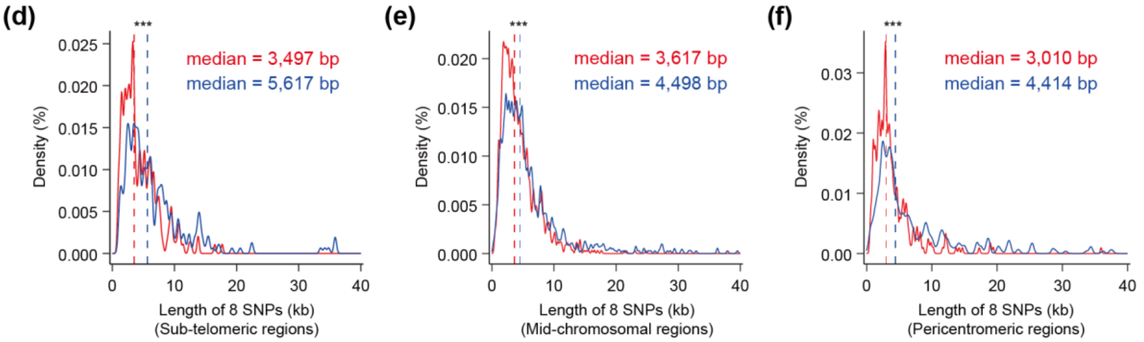

Fig. S15 Genomic region classification based on 8-SNP length and corresponding crossover distributions. (**a**) Genomic regions were classified into four categories based on the length of 8 SNPs flanking each SNP interval. (**b**) The proportion of the genome corresponding to each 8-SNP length category is shown. The proportion of regions with 8-SNP lengths ≥ 15 kb that overlap with *CEN178* repeats or structural variants (SVs) is also indicated. (**c**) Proportions of crossovers occurring within each genomic category. Crossovers detected by GBS and COmapper (from Col × L*er* F_2 and F_1 pollen) are shown separately.

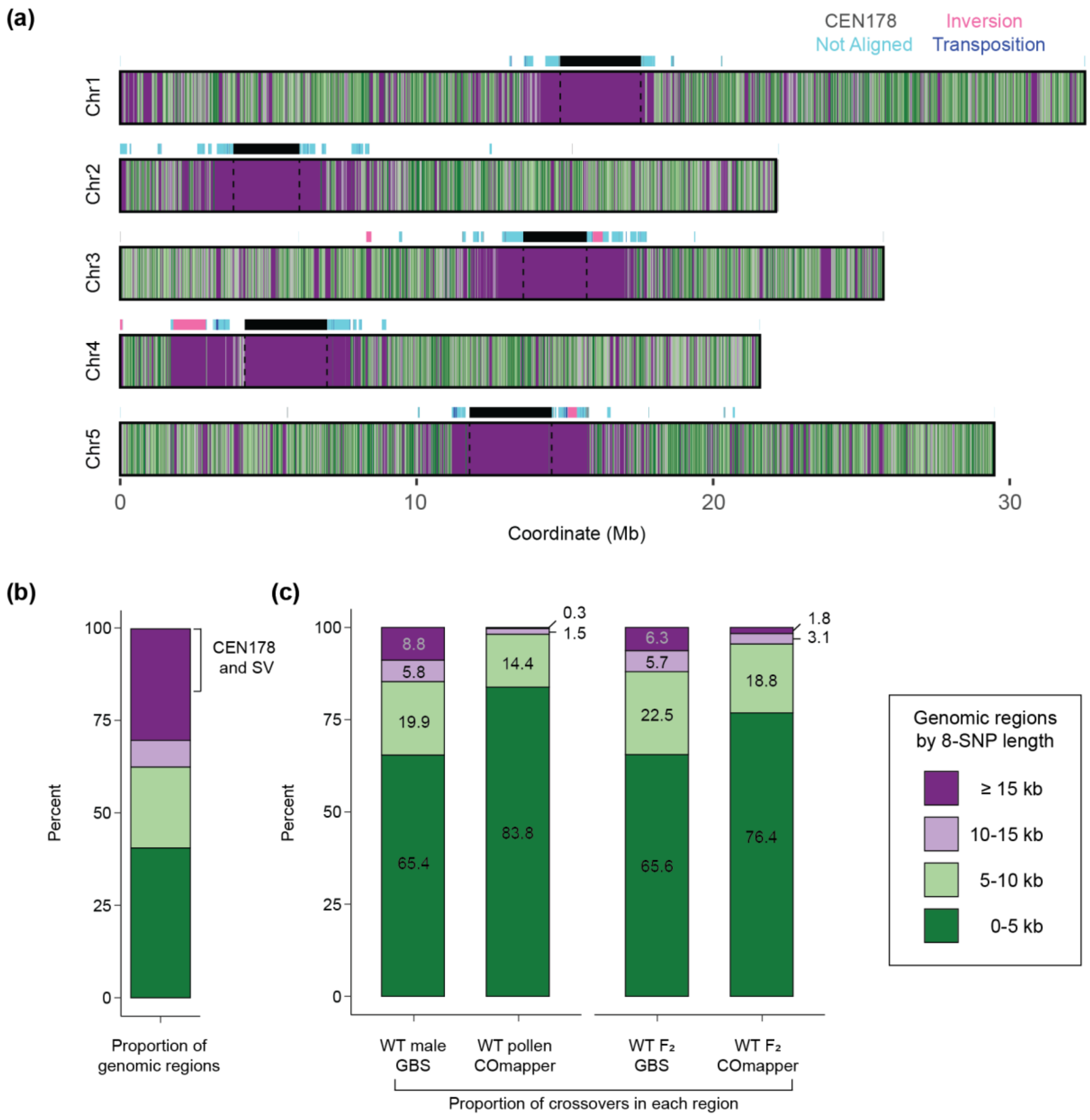

Fig. S16 Crossovers occur more frequently at gene promoters and less frequently at gene bodies than a random distribution. (**a**) Diagram showing the number of 0.5-kb bins around gene transcription start sites (TSS) and end sites (TES) in a 4-kb window for the analysis of crossovers. (**b**) Number of crossovers in each bin for crossovers mapped by GBS (left, Col × L*er* BCF1 plants) or COmapper (right, Col × L*er* F1 pollen) and number of crossovers from a random distribution. Asterisks indicate significant differences compared to the random distribution (**P* < 0.05, ***P* < 0.01, ****P* < 0.001; Wilcoxon test). (**c**) As in (**b**), but showing data for Col × L*er* F_2 plants (GBS) and Col × L*er* F_2 seedlings (COmapper). (**d**) As in (**c**), but showing data for *recq4ab* Col × *recq4ab* L*er* F_2 plants (GBS) and seedlings (COmapper).

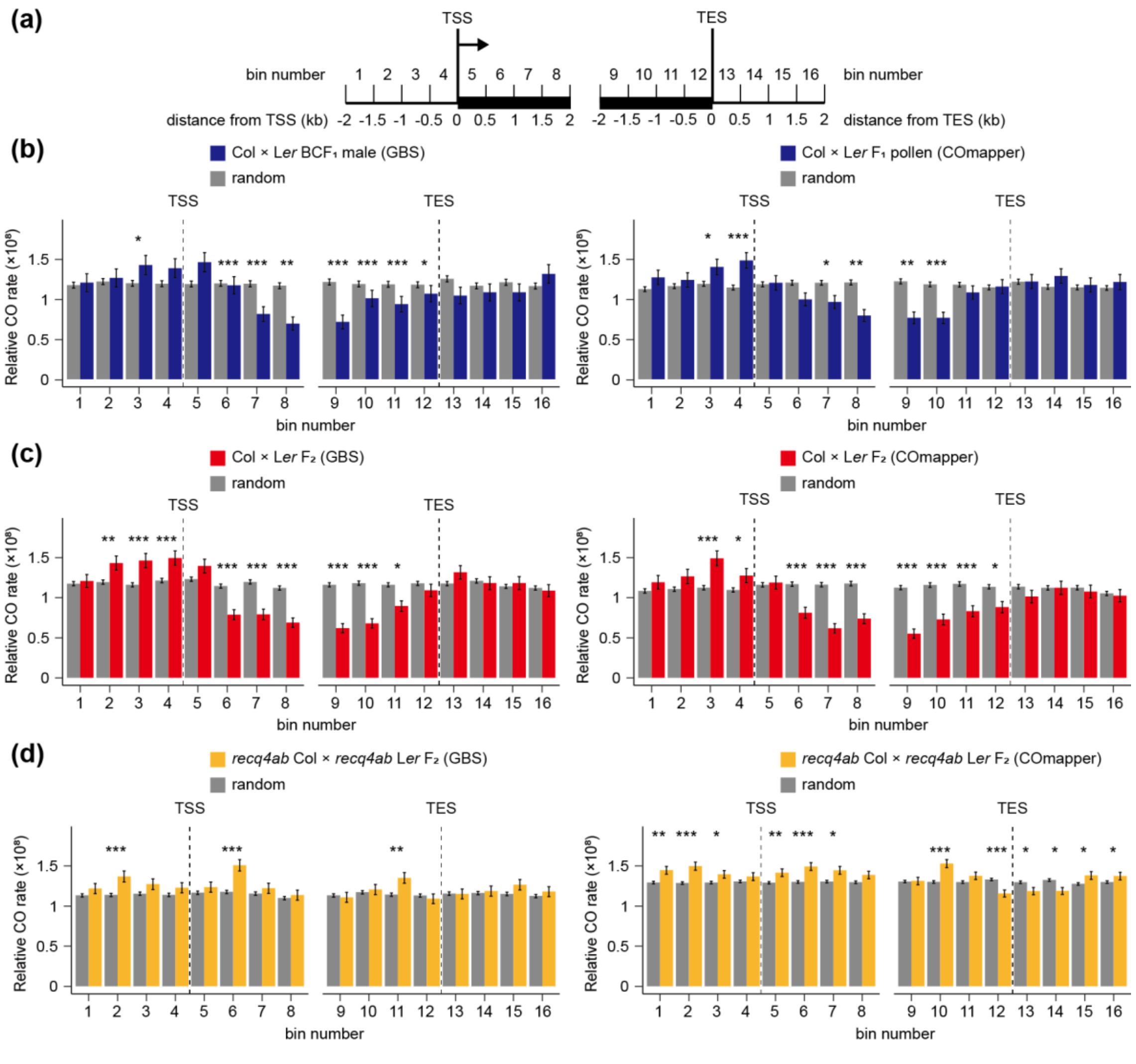

Fig. S17 Resolution of crossovers mapped by GBS and COmapper. (**a**) A model showing the resolution of crossover sites detected by COmapper and GBS. The resolution refers to the distance between the genotyped SNPs flanking the crossover site. COmapper directly determines the crossover site and the resolution is defined as the distance between the closest two SNPs surrounding it. In contrast, the resolution of crossover sites detected by GBS decreases with reduced library sequencing coverage around crossover sites. (**b**) Boxplot comparing resolution of crossovers grouped by sequencing coverage of GBS libraries. Crossover resolution from COmapper of Col × L*er* F_2 seedling and Col × L*er* F_1 pollen is shown. Different lowercase letters indicate significant differences ($P < 0.05$) as determined by ANOVA followed by the Games-Howell test. (**c**) Statistical analysis for resolution of crossovers mapped by GBS and COmapper, with GBS libraries grouped by coverage. SD = standard deviation. SE = standard error. (**d**) Metaplots showing the density of crossovers mapped by GBS. GBS libraries were divided into three coverage groups for plotting.

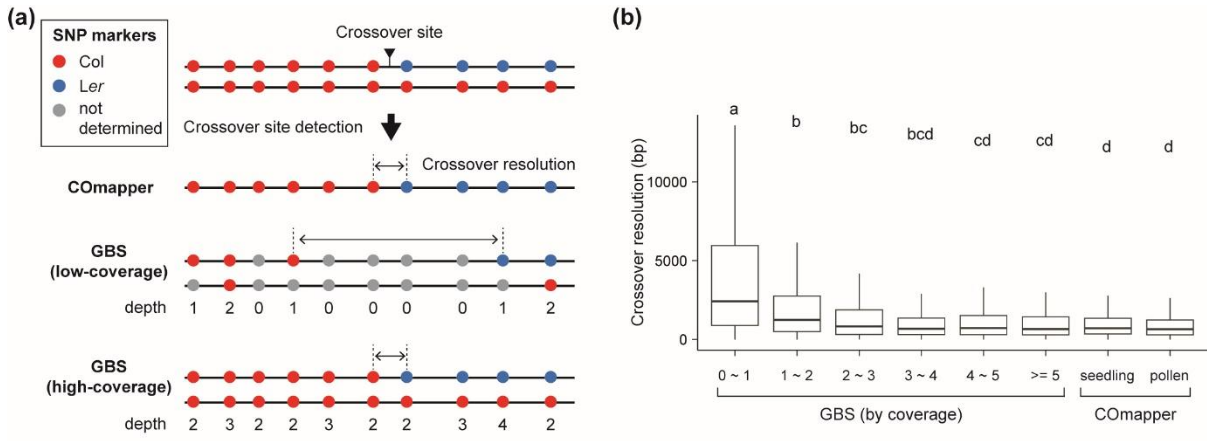

(c)

Method	Group	Libraries	Crossovers	Resolution (bp)			
				Median	Mean	SD	SE
GBS	0 - 1x	777	6,313	2,422	6,106	14,329	180
	1 - 2x	971	7,839	1,247	3,013	8,208	93
	2 - 3x	115	948	830	2,293	8,413	274
	3 - 4x	85	696	684	2,289	10,183	386
	4 - 5x	66	557	729	1,921	6,161	261
	>= 5x	81	577	667	1,835	5,635	235
COmapper	F_2 seedling	1	1,433	716	1,265	2,164	57
	F_1 pollen	1	1,246	658	1,054	1,466	42

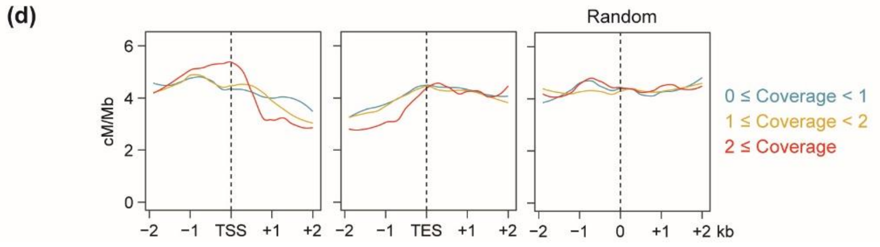

Fig. S18 Class I crossovers are associated with higher SNP density. (**a**) Metaplots showing that the crossovers from F_2 plants derived from a *J3pro:J3*^{G155R} Col × L*er* cross are associated with higher SNP density in a 2-kb window compared to a random distribution, as shown in WT Col × L*er* hybrid F_2 plants (Fig. **6a**). *n* indicates the number of crossover sites mapped by GBS or the number of randomly chosen regions. The left plot shows SNP density around crossovers mapped by GBS compared to random (middle plot). The right plot shows the significance of changes in SNP density according to the physical distance (bp) from the crossover site. Black, green and red dots indicate SNP density per 40-bp bin. Significance between genotype and the random distribution per 40-bp bin was tested using a Wilcoxon test. Red and blue dots above the plot indicate higher and lower SNP density than a random distribution, respectively, while gray dots indicate non-significance. (**b**) As in (**a**), but showing the crossovers mapped by GBS in F_2 individual plants from self-pollinated Col × Di-G F_1 hybrids.

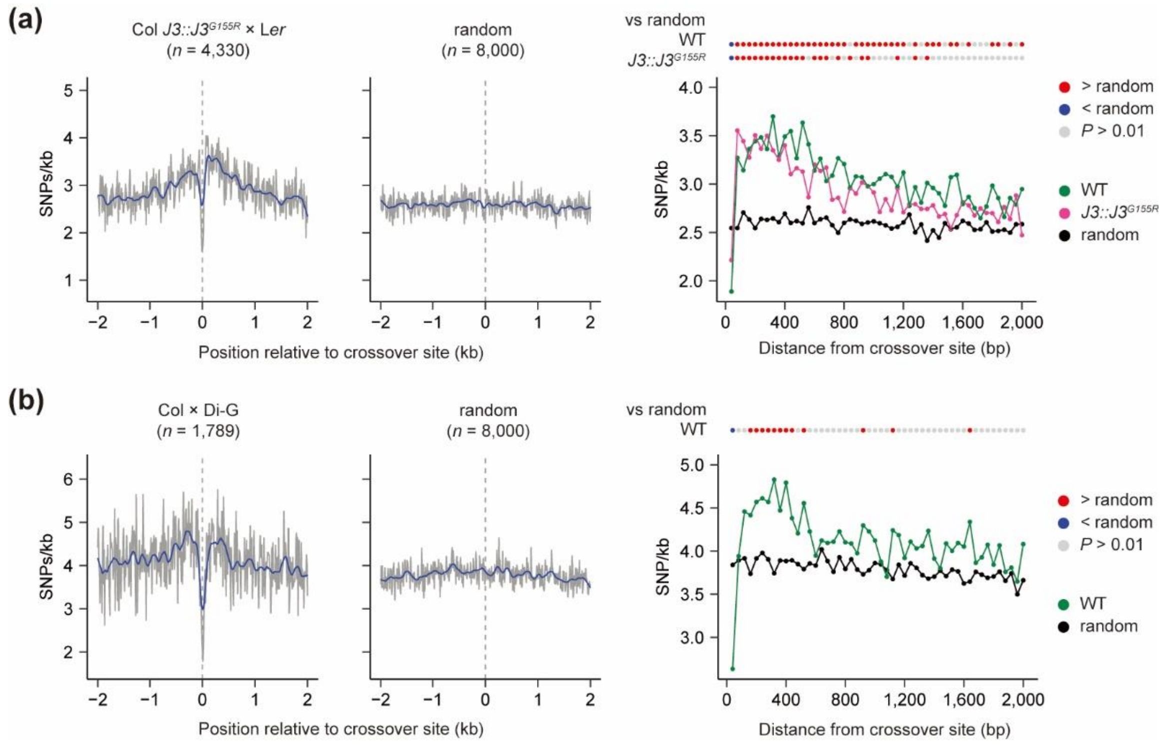

Fig. S19 Analysis of SNP density between Solanum *lycopersicum* and Solanum *pimpinellifolium.* (**a**) Genomic regions were classified into four categories based on the length of 8 SNPs flanking each SNP interval. (**b**) The proportion of the genome corresponding to each 8-SNP length category is shown.

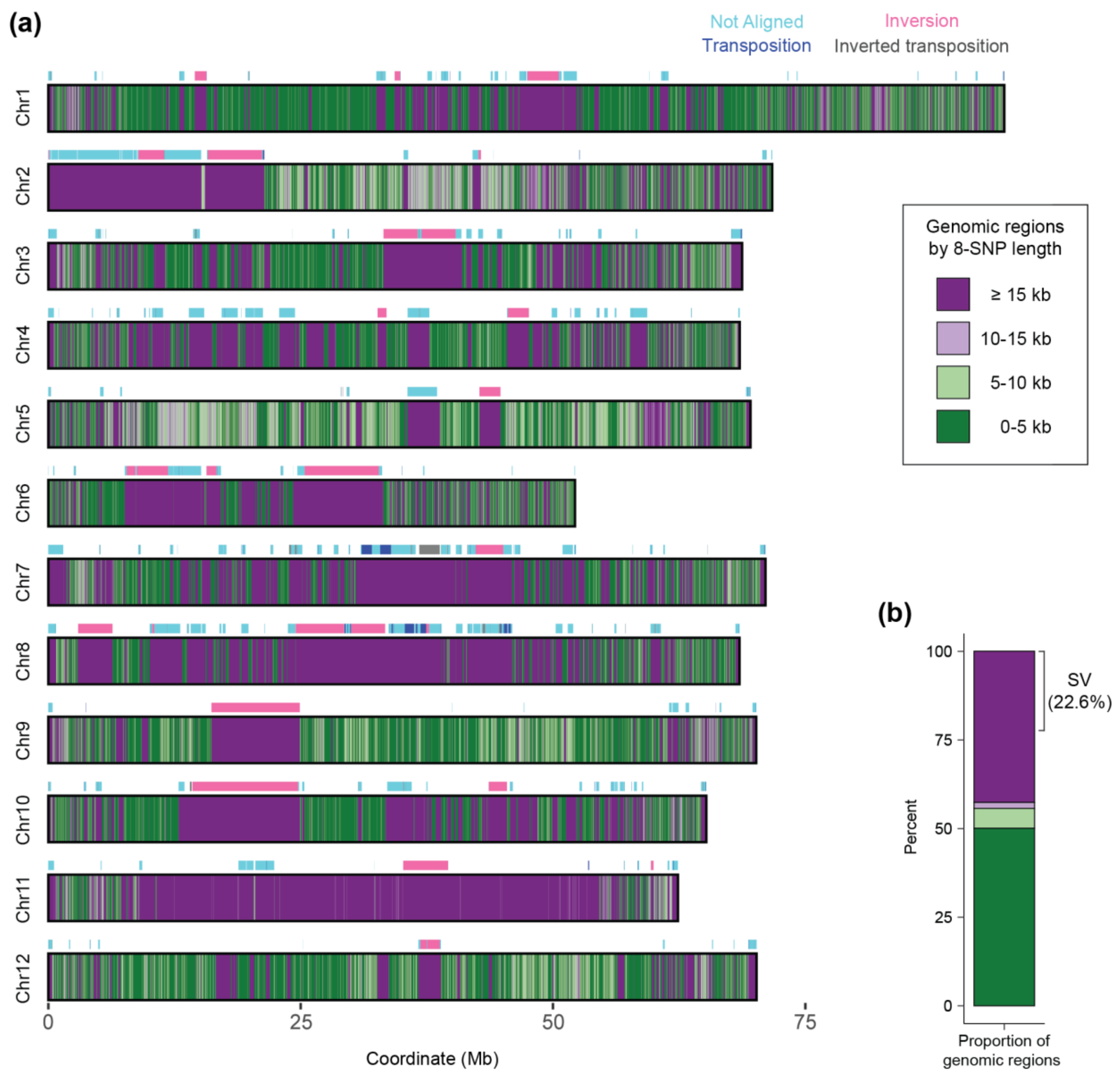

Table S1. Summary of long-read nanopore sequencing libraries used in this study. Ten samples with different genotypes and tissues were used to generate long-read nanopore sequencing libraries using high-molecular-weight genomic DNA. *n* indicates the number of pooled Col × L*er* F_2 individual plants. The information for the crossover analysis by COmapper is provided for each library.

Genotype Tissue	Aligned reads	Aligned bases (Gb)	Crossover reads (allows overlap)	Parental reads	Crossovers /haploid
Col × L*er* F_1 leaves	1,095,831	13.99	2	561,814	0.03
recq4ab Col × *recq4ab* L*er* F_1 leaves	817,791	12.33	0	436,702	0.00
Col × L*er* F_2 leaves (*n* = 4)	3,429,425	40.14	630	1,772,364	2.79
Col × L*er* F_2 leaves (*n* = 46)	11,345,385	100.74	1,659	4,895,997	2.99
recq4ab Col × *recq4ab* L*er* F_2 leaves (*n* = 39)	9,747,722	48.83	1,448	3,546,114	7.87
Col × L*er* F_1 pollen set1	14,041,724	149.34	1,146	9,773,260	1.59
Col × L*er* F_1 pollen set2	4,557,585	61.98	533	4,294,489	1.70
recq4ab Col × *recq4ab* L*er* F_1 pollen	8,789,632	126.66	1,963	4,988,761	3.92
Col × L*er* F_2 seedlings (*n* = 1000)	8,433,348	96.14	1,679	3,553,262	3.22
recq4ab Col × *recq4ab* L*er* F_1 seedlings (*n* = 1000)	9,980,173	95.35	6,719	4,685,186	12.42

Table S2. Detailed information for the sequencing libraries used in this study. The short-read next-generation and long-read nanopore sequencing libraries used in this study are listed and their information is provided. All data used in this study have been deposited in the ArrayExpress database at EMBL-EBL (http://www.ebi.ac.uk/arrayexpress).

Library	Dataset accession	Genotype	Read length	Tissue	References
gDNA	E-MTAB-6257	Col	2×100 bp	Floral buds	(Choi et al. 2018)
SPO11-1–oligo	E-MTAB-5041	Col	2×100 bp	Floral buds	(Choi et al. 2018)
MNase-seq	E-MTAB-5042	Col	2×100 bp	Floral buds	(Choi et al. 2018)
GBS	E-MTAB-8165<br>E-MTAB-10168<br>E-MTAB-10657	Col × *Ler*	2×150 bp	Leaves	(Rowan *et al.*, 2019; Nageswaran *et al.*, 2021; Kim *et al.*, 2022, 2024)
GBS	E-MTAB-12726	*recq4ab* Col × *recq4ab Ler*	2×150 bp	F_2 leaves	(Kim *et al.*, 2024)
GBS	E-MTAB-13414	*J3pro:J3*^{G155R} Col × *Ler*	2×150 bp	F_2 leaves	(Kim *et al.*, 2024)
GBS	E-MTAB-14382	Col × Di-G	2×150 bp	F_2 leaves	This study
GBS	E-MTAB-14380	Col × *Ler*	2×150 bp	50 F_2 leaves	This study
GBS	E-MTAB-12695<br>E-MTAB-15084	*Ler* × Col/*Ler*	2×150 bp	BCF_1 leaves	This study
GBS	E-MTAB-15081	*recq4ab Ler* × *recq4ab* Col/*Ler*	2×150 bp	BCF_1 leaves	This study
Nanopore-seq	E-MTAB-14381	Col × *Ler*	~13.5 kb	F_1 leaves	This study
Nanopore-seq	E-MTAB-14381	*recq4ab* Col × *recq4ab Ler*	~15.9 kb	F_1 leaves	This study
Nanopore-seq	E-MTAB-14367	Col × *Ler*	~12.3 kb	4 F_2 leaves	This study
Nanopore-seq	E-MTAB-14367	Col × *Ler*	~9.8 kb	46 F_2 leaves	This study
Nanopore-seq	E-MTAB-15082	*recq4ab* Col × *recq4ab Ler*	~9.1 kb	39 F_2 leaves	This study
Nanopore-seq	E-MTAB-14369	Col × *Ler*	~14.1 kb	F_1 pollen	This study
Nanopore-seq	E-MTAB-15083	Col × *Ler*	~6.8 kb	F_1 pollen	This study
Nanopore-seq	E-MTAB-14369	*recq4ab* Col × *recq4ab Ler*	~19.3 kb	F_1 pollen	This study
Nanopore-seq	E-MTAB-14368	Col × *Ler*	~20.5 kb	F_2 seedlings	This study
Nanopore-seq	E-MTAB-14368	*recq4ab* Col × *recq4ab Ler*	~16.0 kb	F_2 seedling	This study

Table S3. Comparison of genome-wide crossover mapping techniques. Summarizes key features of four genome-wide crossover (CO) mapping techniques: GBS, linked-read sequencing, Hi-C, and COmapper. Each method differs in detection principle, resolution, and data output.

Feature	GBS	Linked-read-seq	Hi-C	COmapper
Detection Principle	- Short read seq<br>- F_2 individuals	- Short read seq<br>- Reconstruct long DNA<br>- 10X index<br>- Sample pool	- Shor read seq<br>- Hi-C fragments<br>- Read-pairs<br>- Sample pool	- Long read seq<br>- Sample pool
Crossover Detection	- Indirect<br>- Chromosome	- Indirect<br>- Large molecule	- Indirect<br>- Proximity-ligated molecule	- Direct<br>- Long read
Resolution	- High resolution with high coverage	- High resolution with high coverage	- Low resolution	- High resolution<br>- Single long read
Advantages	- Available on high/low coverage<br>- Individual information<br>- Low SNP density	- High resolution<br>- Pooled sample<br>- Low SNP density	- Chromosome structure<br>- Low SNP density	- High resolution<br>- Pooled sample<br>- Simple library prep
Disadvantages	- Individual index<br>- Difficult library prep	- Difficult library prep<br>- Missing individual data	- Low resolution<br>- Difficult library prep<br>- Missing individual data	- Missing individual data<br>- High SNP density

Dataset S1. Allele frequency maps of 46 and 4 F_2 individuals. Haplotypes inferred by TIGER are shown as colored segments. Crossover sites missed by TIGER are indicated with red arrows.

46 F_2 individuals

Lib 1

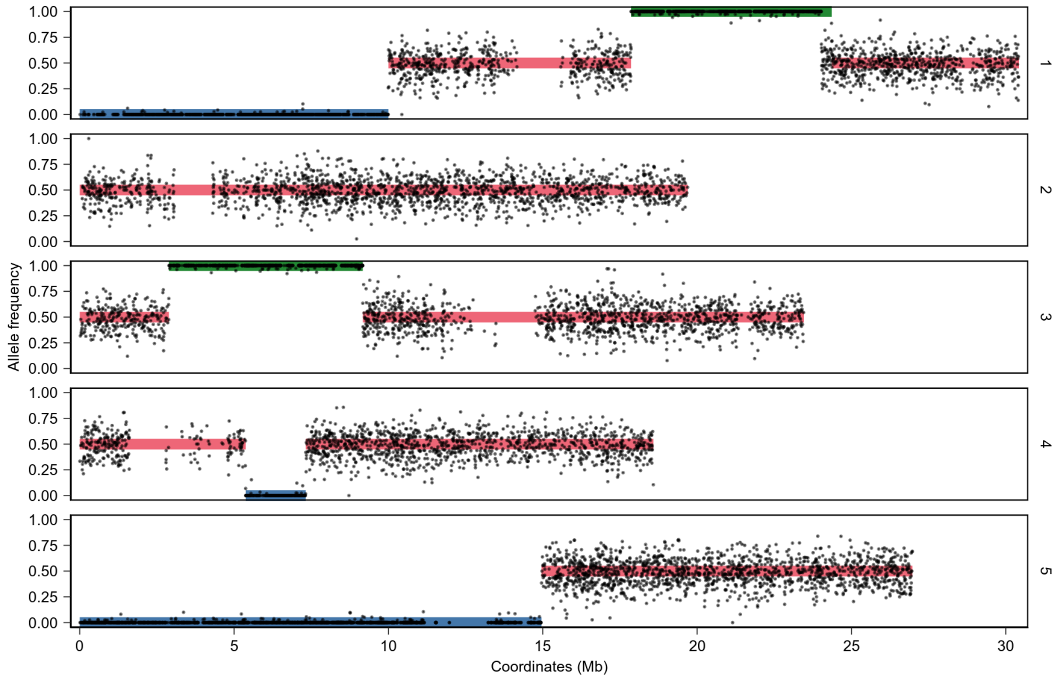

Allele frequency
1.00
0.75
0.50
0.25
0.00
1
2
3
4
5
0
5
10
15
20
25
30
Coordinates (Mb)

Lib 2

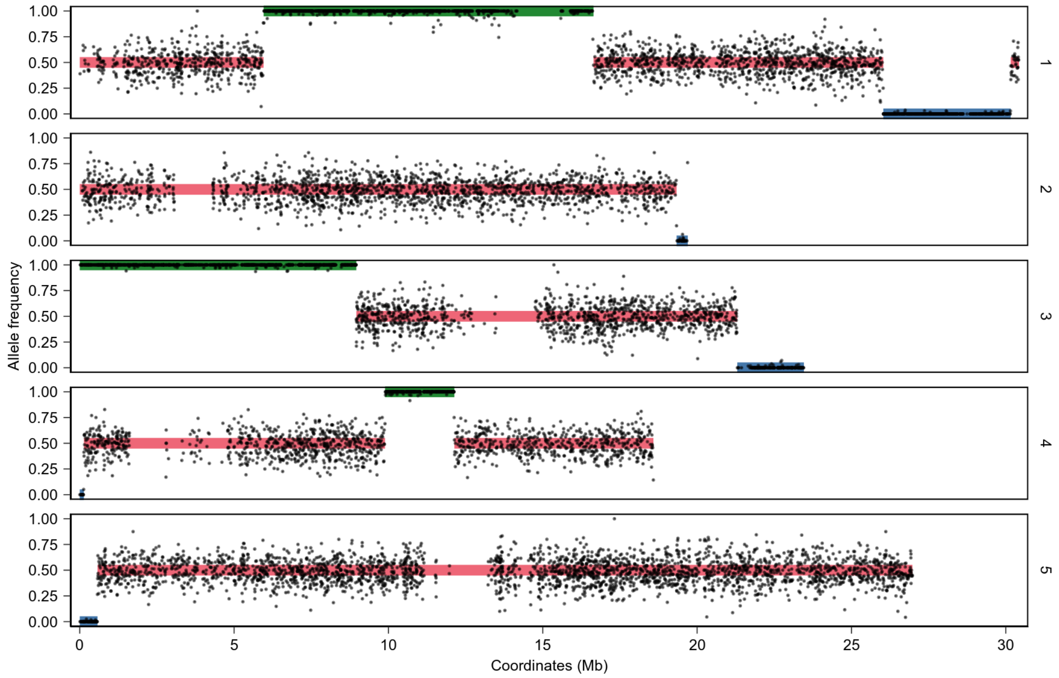

Allele frequency
1.00
0.75
0.50
0.25
0.00
1
2
3
4
5
0
5
10
15
20
25
30
Coordinates (Mb)

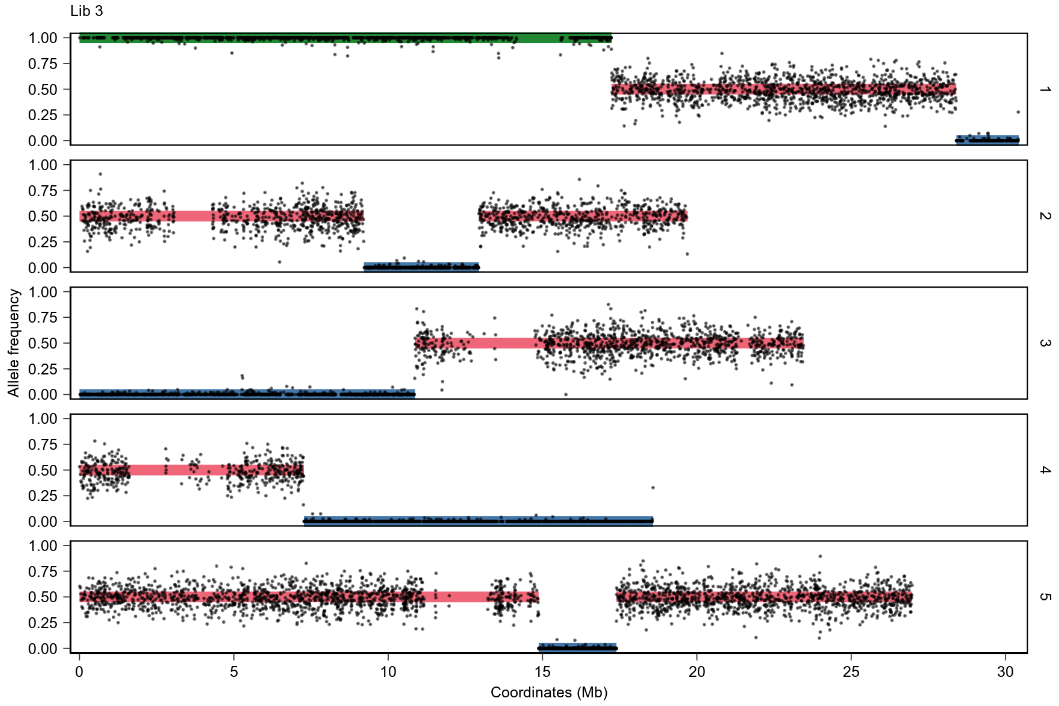
Lib 3
Allele frequency
Coordinates (Mb)
1
2
3
4
5

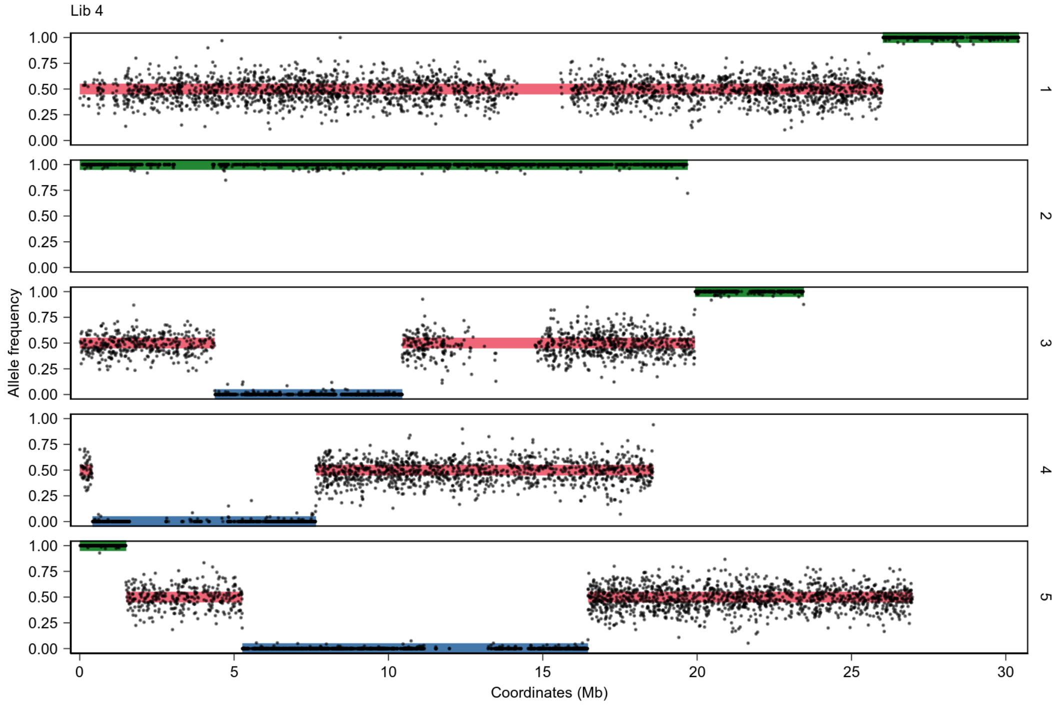
Lib 4
Allele frequency
Coordinates (Mb)
1
2
3
4
5

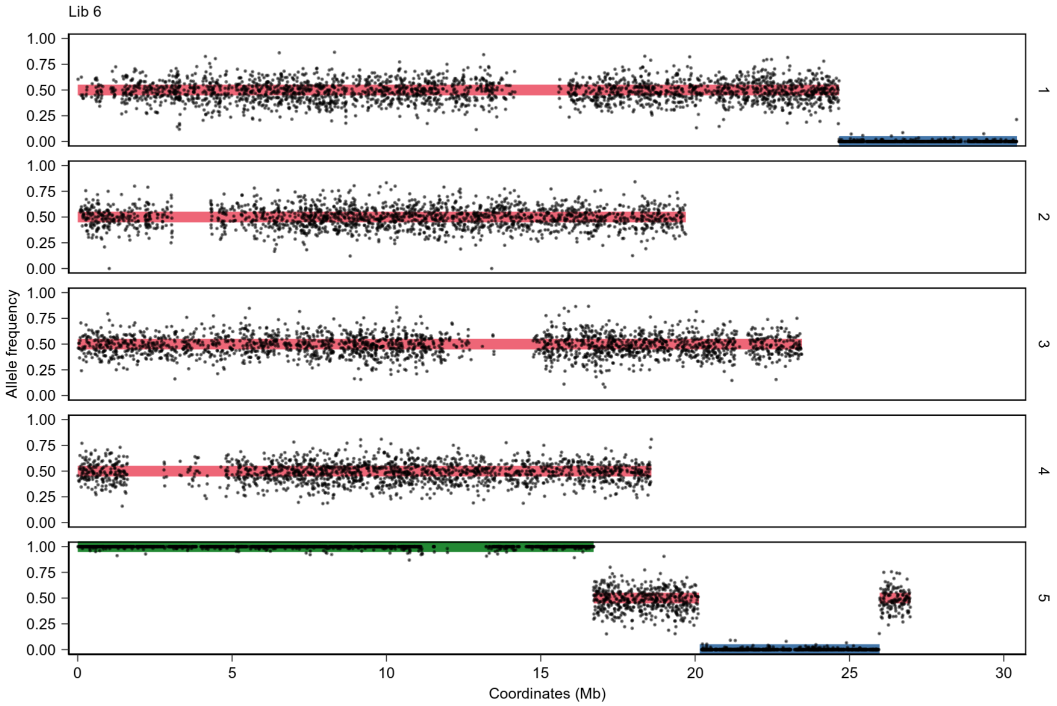
Lib 6
Allele frequency
1.00
0.75
0.50
0.25
0.00
1
2
3
4
5
0
5
10
15
20
25
30
Coordinates (Mb)

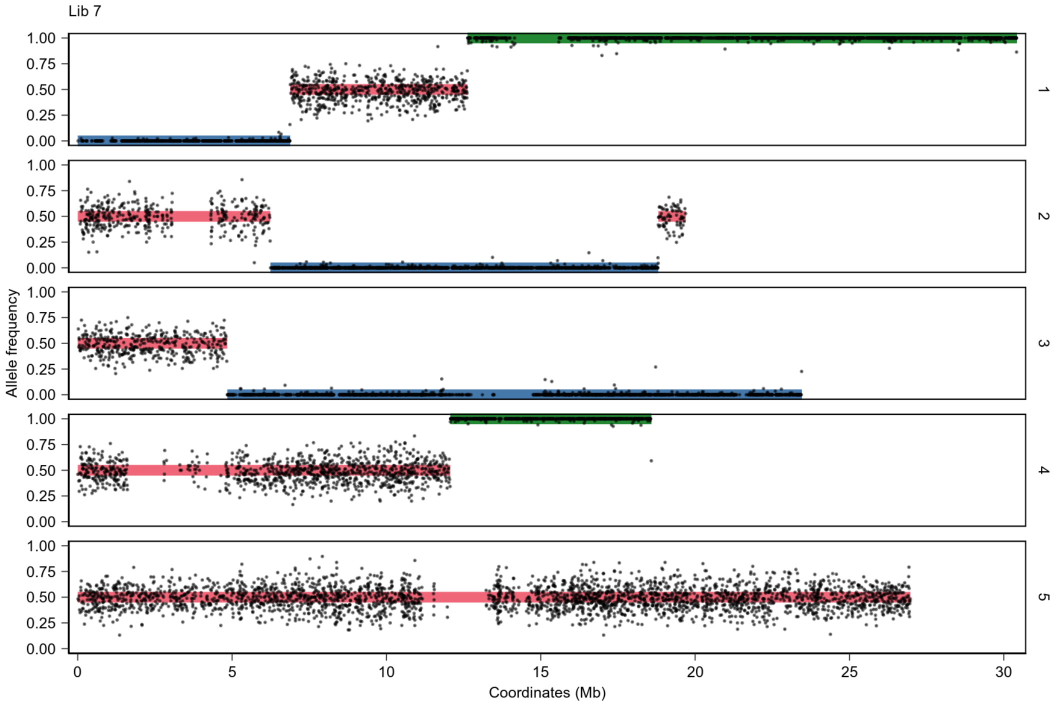
Lib 7
Allele frequency
1.00
0.75
0.50
0.25
0.00
1
2
3
4
5
0
5
10
15
20
25
30
Coordinates (Mb)

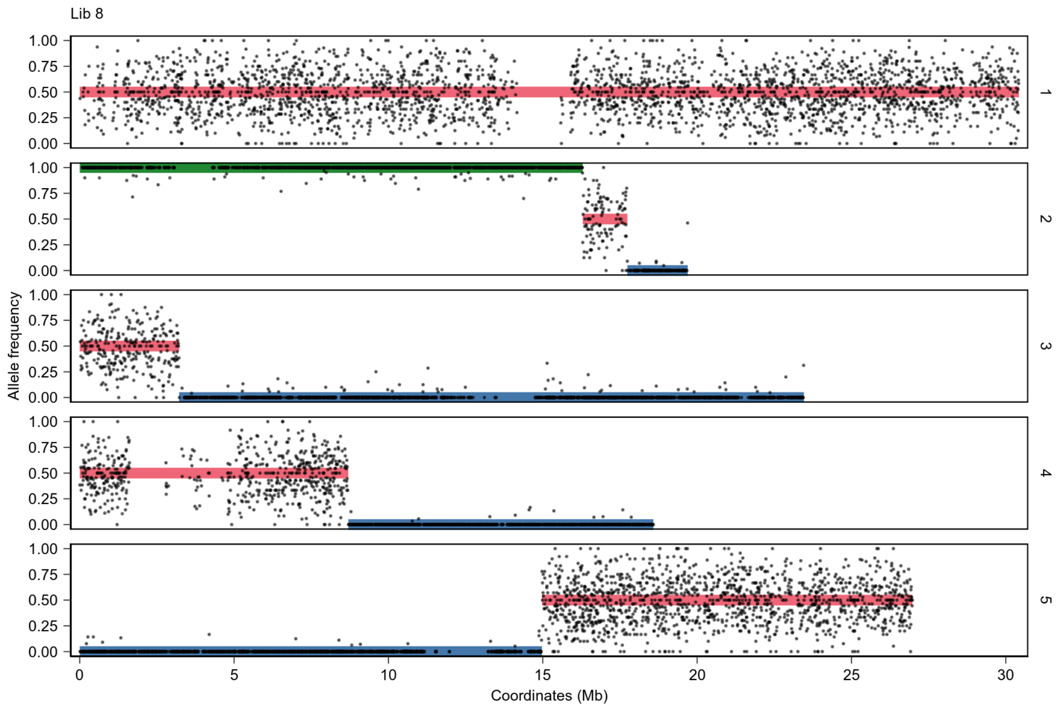
Lib 8
1.00
0.75
0.50
0.25
0.00
Allele frequency
1
2
3
4
5
0
5
10
15
20
25
30
Coordinates (Mb)

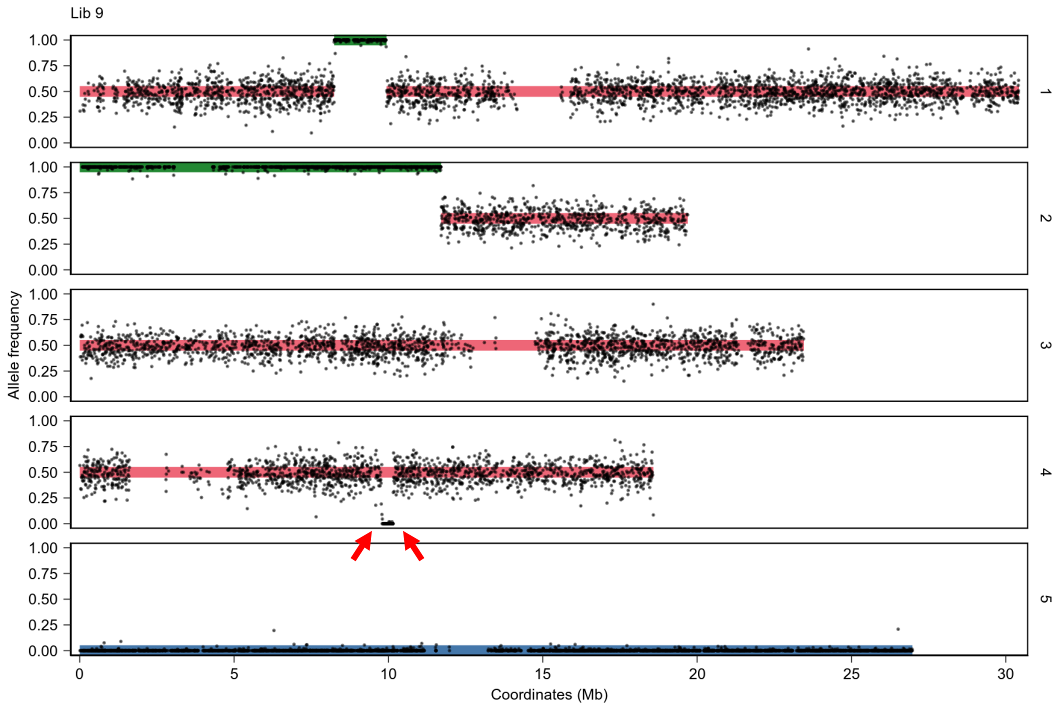
Lib 9
1.00
0.75
0.50
0.25
0.00
Allele frequency
1
2
3
4
5
0
5
10
15
20
25
30
Coordinates (Mb)

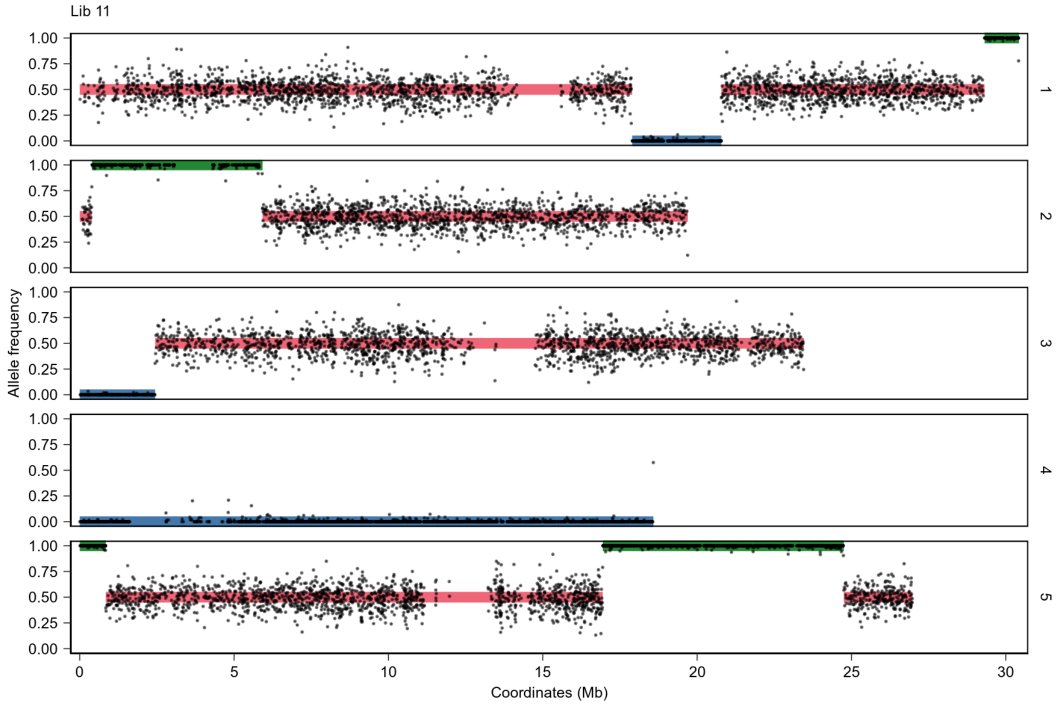

Lib 11
1.00
0.75
0.50
0.25
0.00
Allele frequency
1
2
3
4
5
0
5
10
15
20
25
30
Coordinates (Mb)

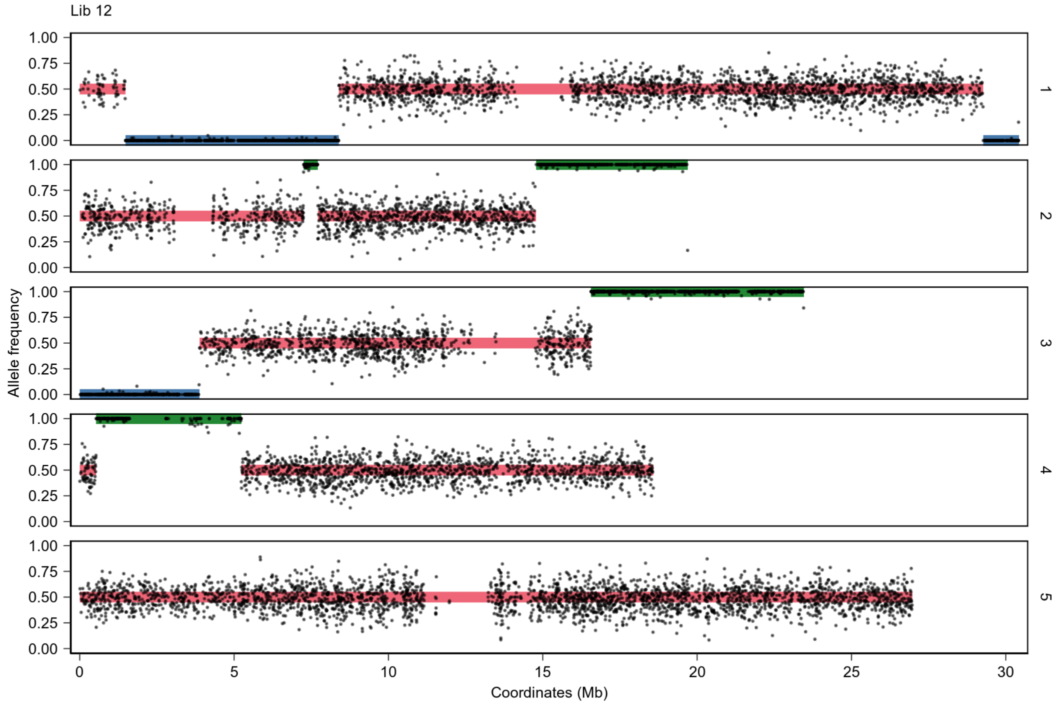

Lib 12
1.00
0.75
0.50
0.25
0.00
Allele frequency
1
2
3
4
5
0
5
10
15
20
25
30
Coordinates (Mb)

Lib 13

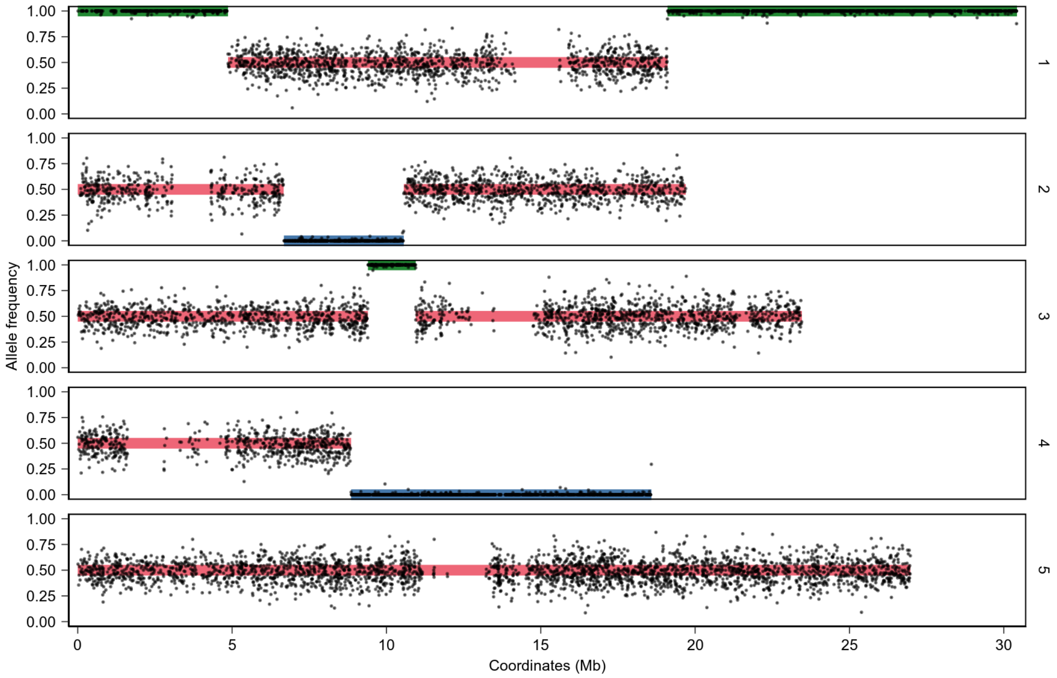

1.00
0.75
0.50
0.25
0.00
1
2
3
4
5
Allele frequency
0
5
10
15
20
25
30
Coordinates (Mb)

Lib 14

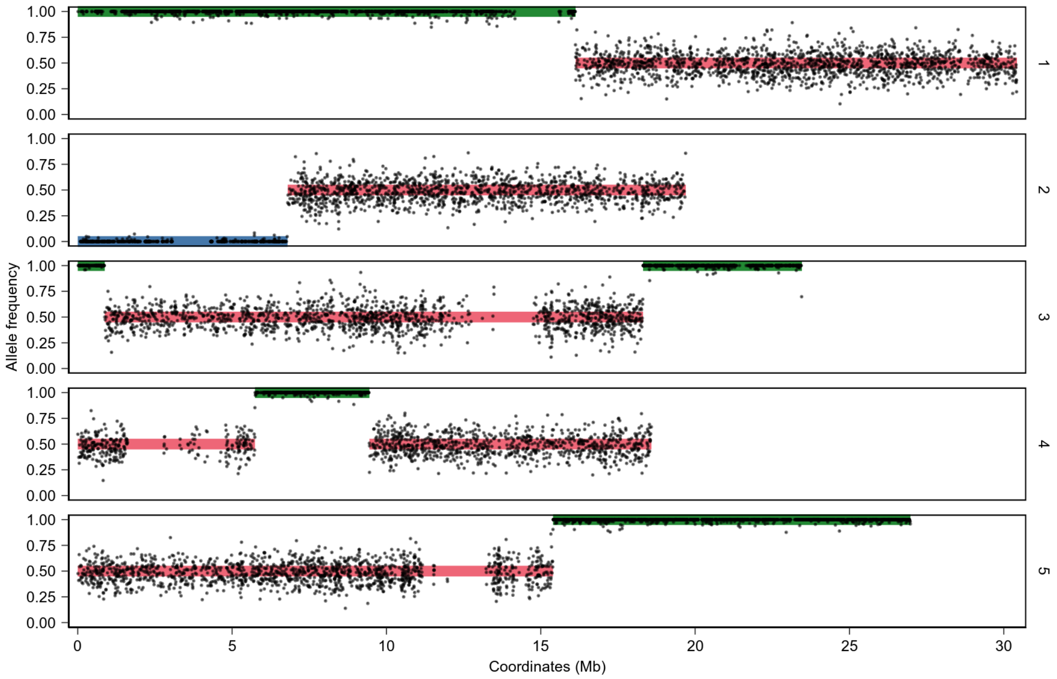

1.00
0.75
0.50
0.25
0.00
1
2
3
4
5
Allele frequency
0
5
10
15
20
25
30
Coordinates (Mb)

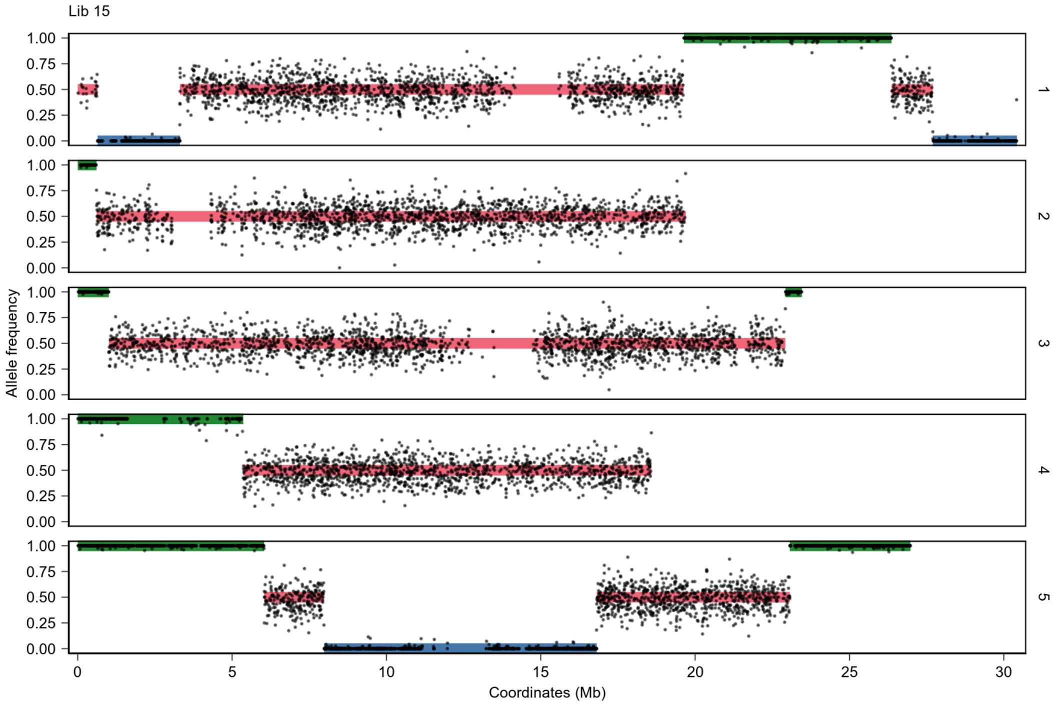
Lib 15
1.00
0.75
0.50
0.25
0.00
Allele frequency
1
2
3
4
5
0
5
10
15
20
25
30
Coordinates (Mb)

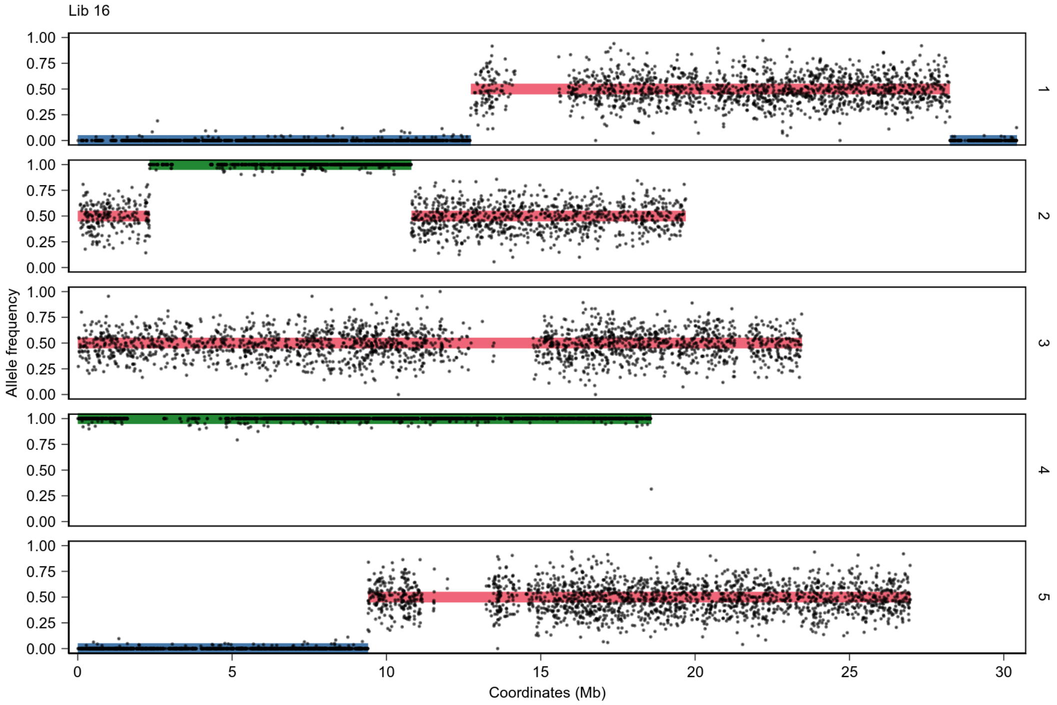
Lib 16
1.00
0.75
0.50
0.25
0.00
Allele frequency
1
2
3
4
5
0
5
10
15
20
25
30
Coordinates (Mb)

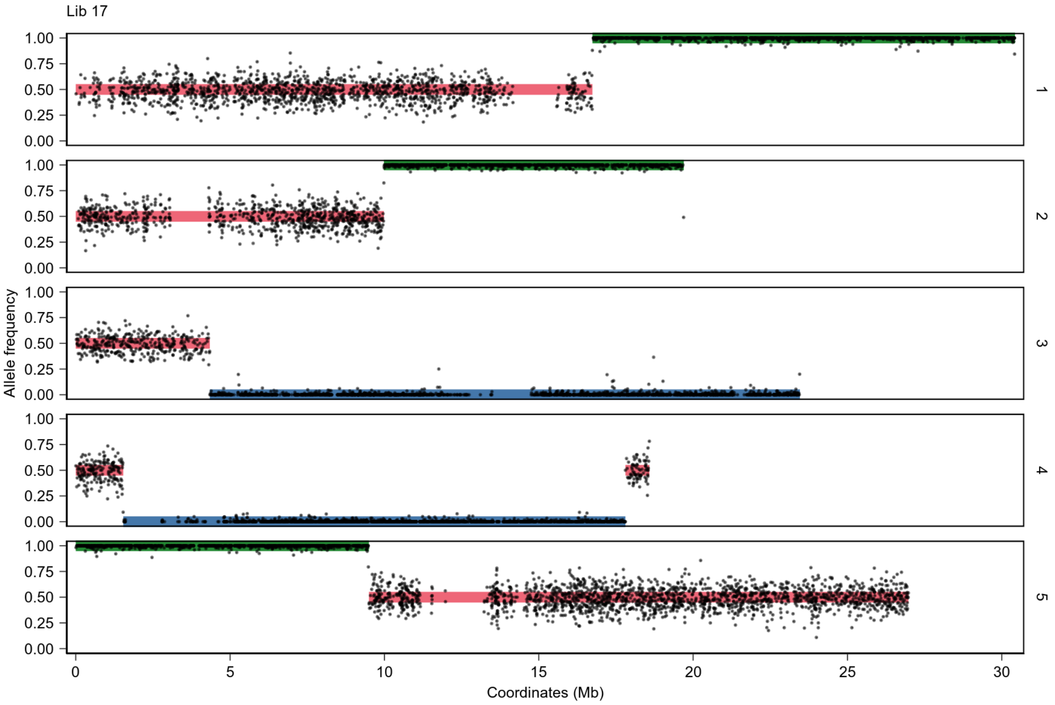
Lib 17
Allele frequency
Coordinates (Mb)
1
2
3
4
5

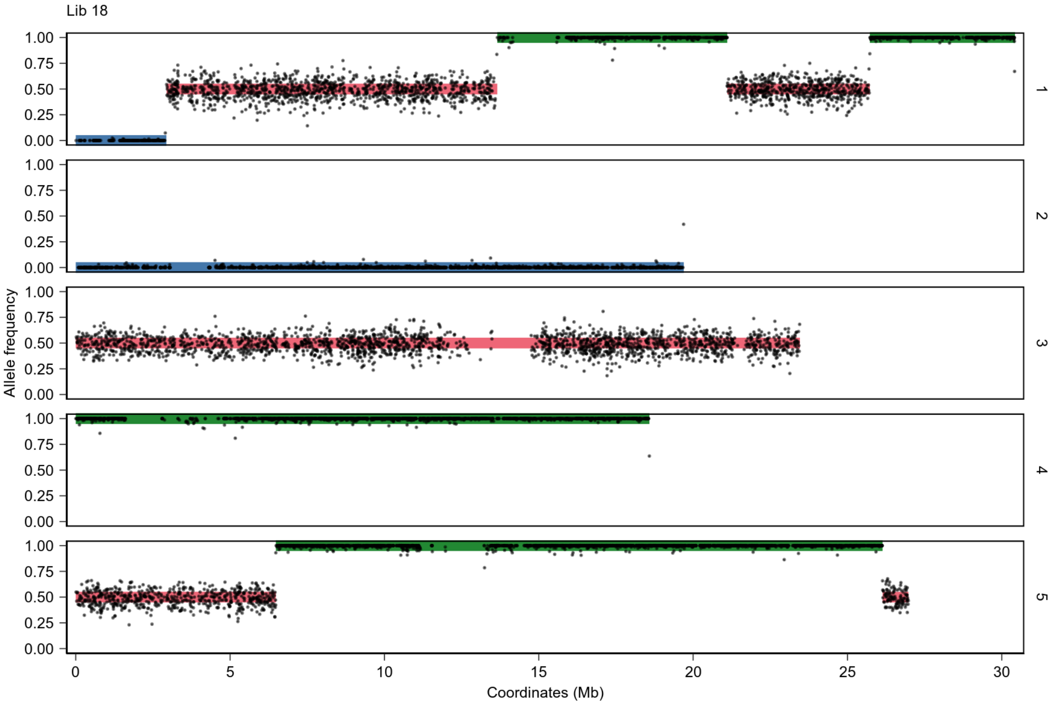
Lib 18
Allele frequency
Coordinates (Mb)
1
2
3
4
5

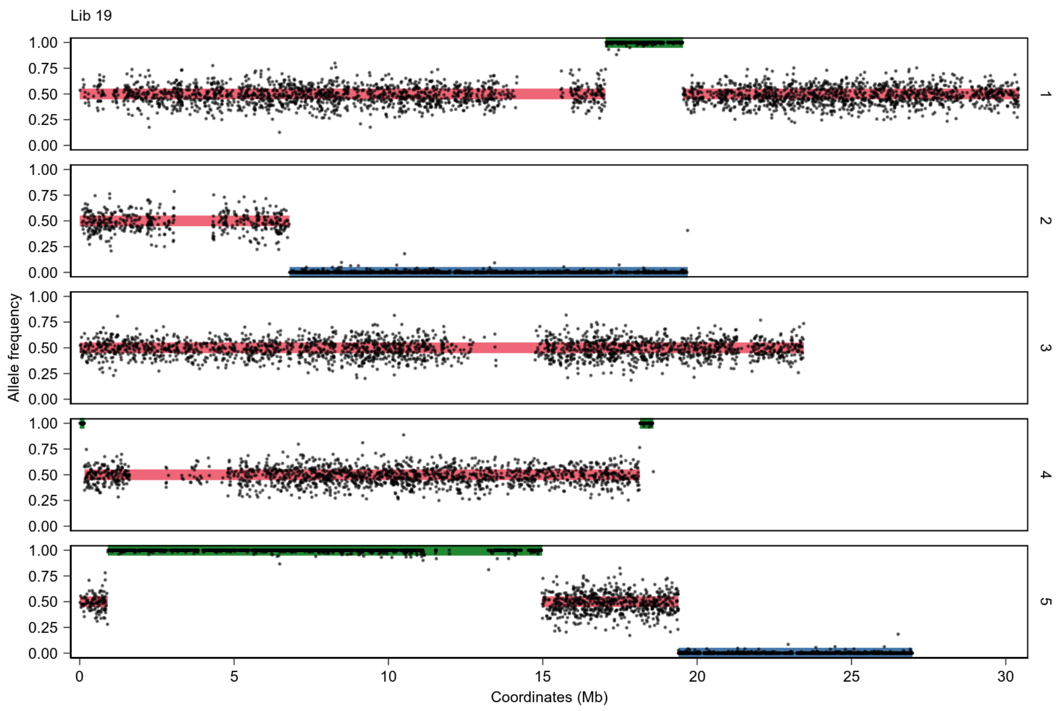
Lib 19
Allele frequency
Coordinates (Mb)
1.00
0.75
0.50
0.25
0.00
0
5
10
15
20
25
30
1
2
3
4
5

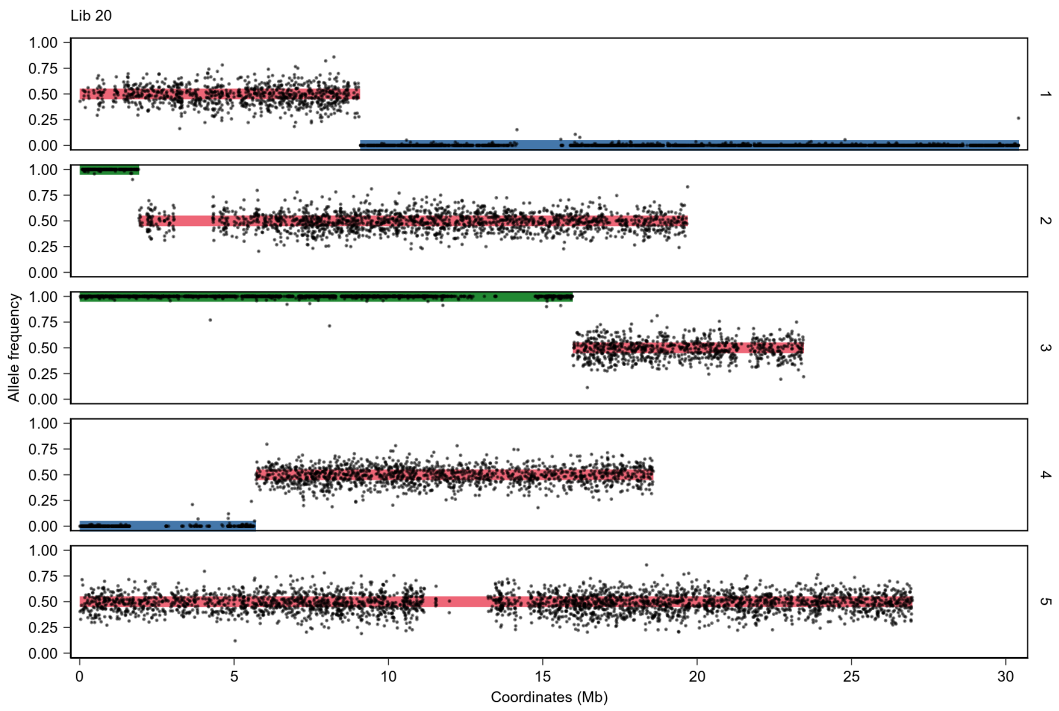
Lib 20
Allele frequency
Coordinates (Mb)
1.00
0.75
0.50
0.25
0.00
0
5
10
15
20
25
30
1
2
3
4
5

Lib 21

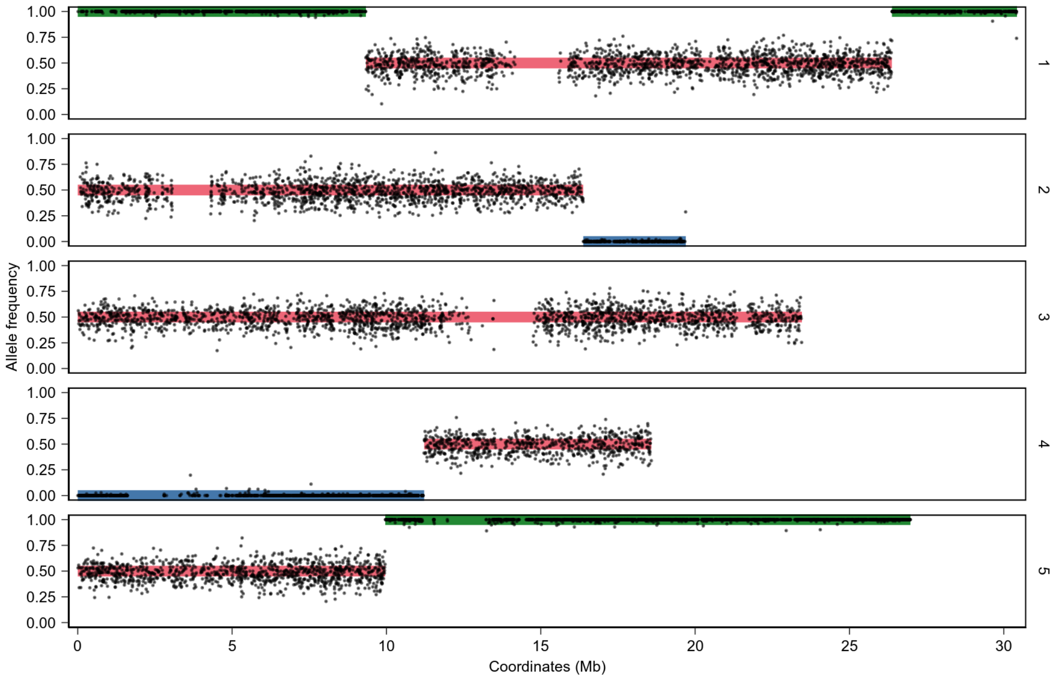

Allele frequency
1.00
0.75
0.50
0.25
0.00
1
2
3
4
5
0
5
10
15
20
25
30
Coordinates (Mb)

Lib 22

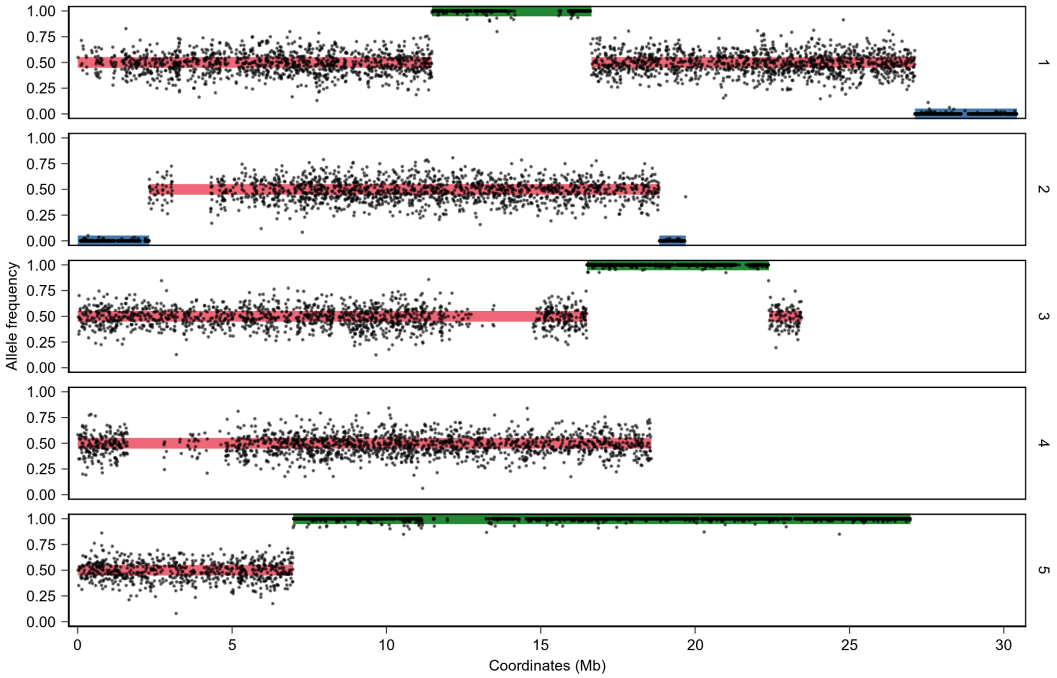

Allele frequency
1.00
0.75
0.50
0.25
0.00
1
2
3
4
5
0
5
10
15
20
25
30
Coordinates (Mb)

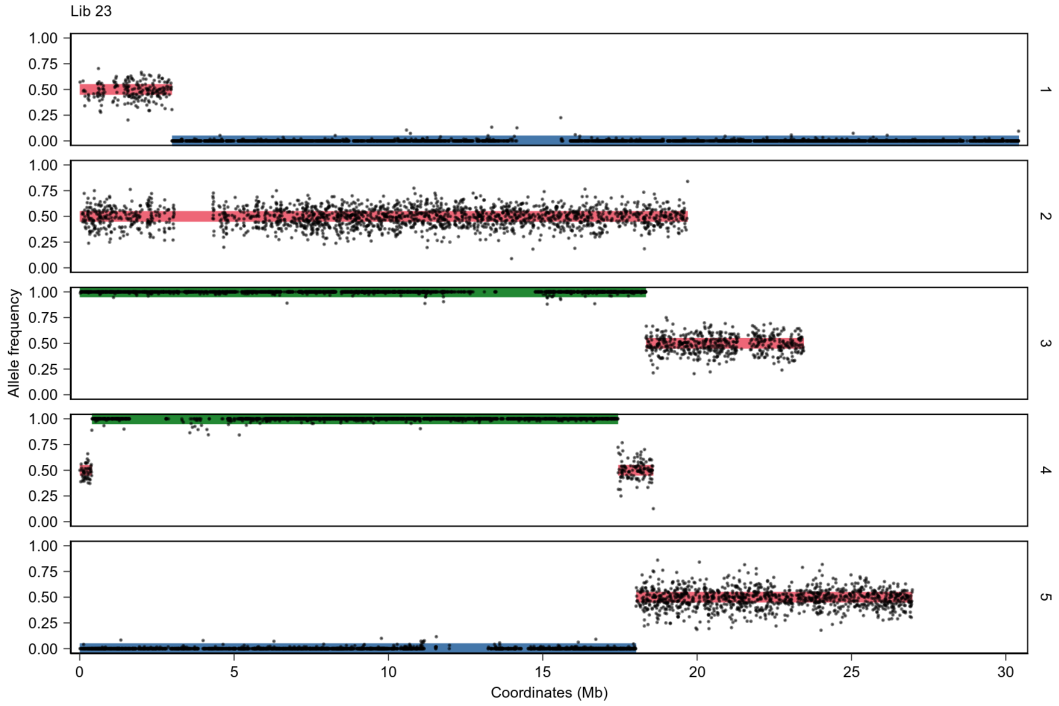
Lib 23
1.00
0.75
0.50
0.25
0.00
Allele frequency
1
2
3
4
5
0
5
10
15
20
25
30
Coordinates (Mb)

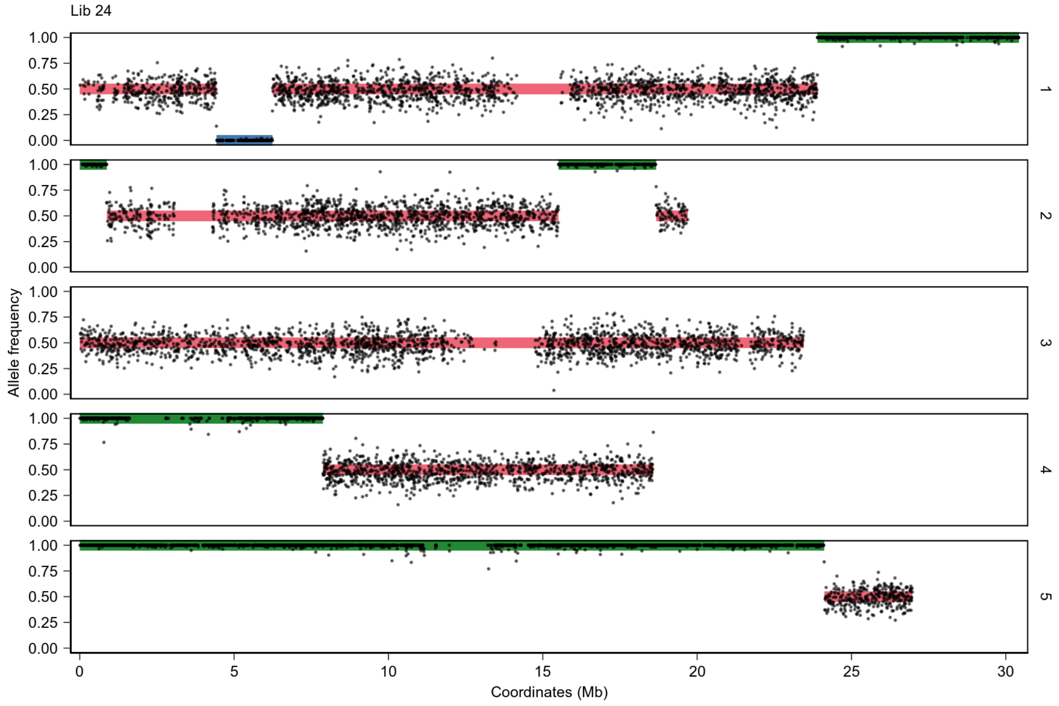
Lib 24
1.00
0.75
0.50
0.25
0.00
Allele frequency
1
2
3
4
5
0
5
10
15
20
25
30
Coordinates (Mb)

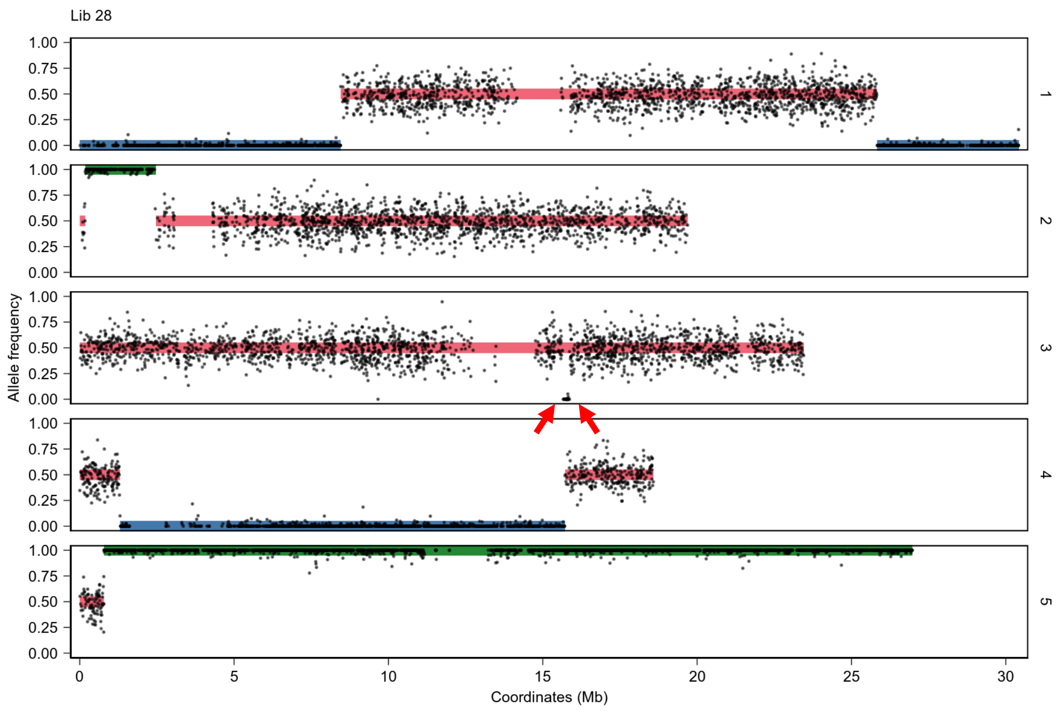
Lib 28
Allele frequency
Coordinates (Mb)
1.00
0.75
0.50
0.25
0.00
0
5
10
15
20
25
30
1
2
3
4
5

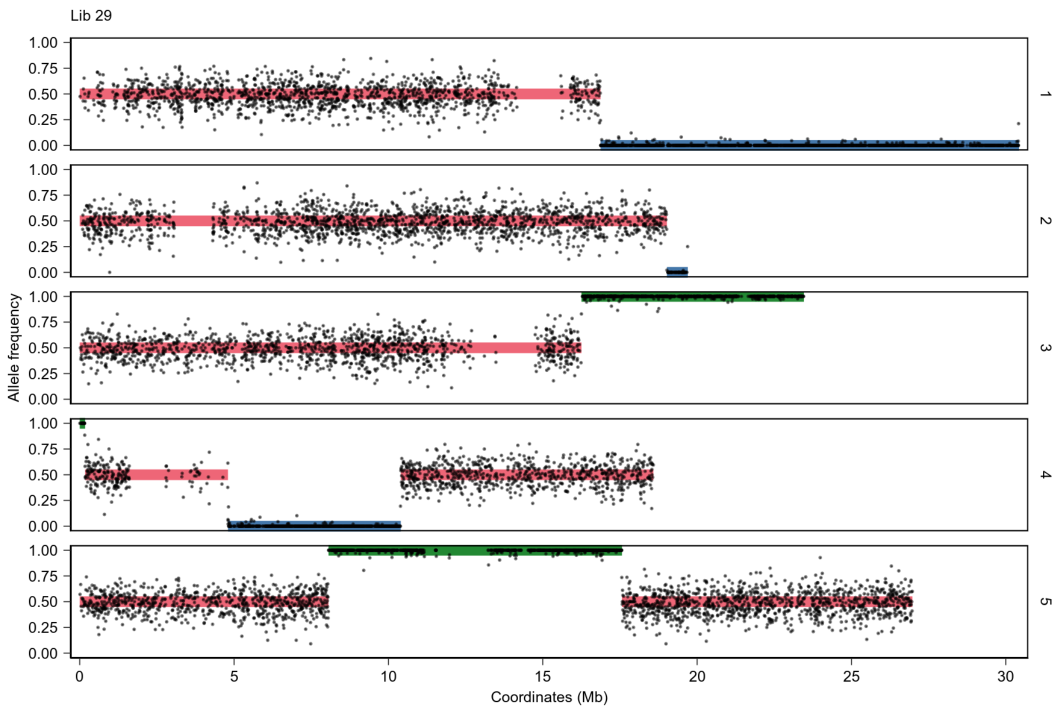
Lib 29
Allele frequency
Coordinates (Mb)
1.00
0.75
0.50
0.25
0.00
0
5
10
15
20
25
30
1
2
3
4
5

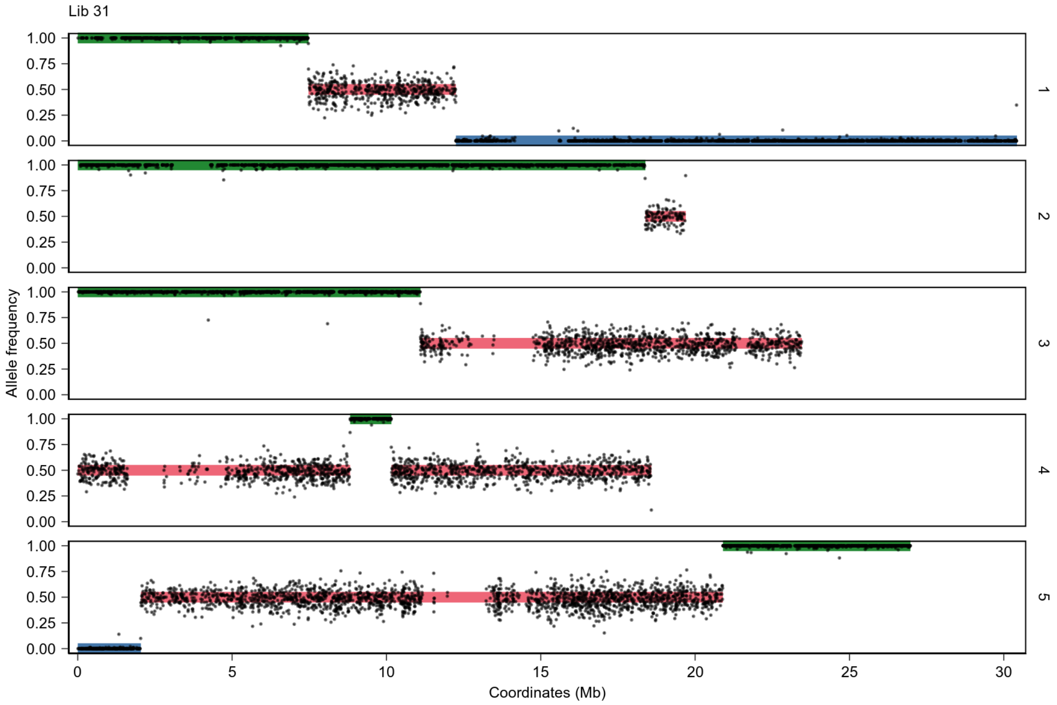

Lib 31
1.00
0.75
0.50
0.25
0.00
1
2
3
4
5
Allele frequency
0
5
10
15
20
25
30
Coordinates (Mb)

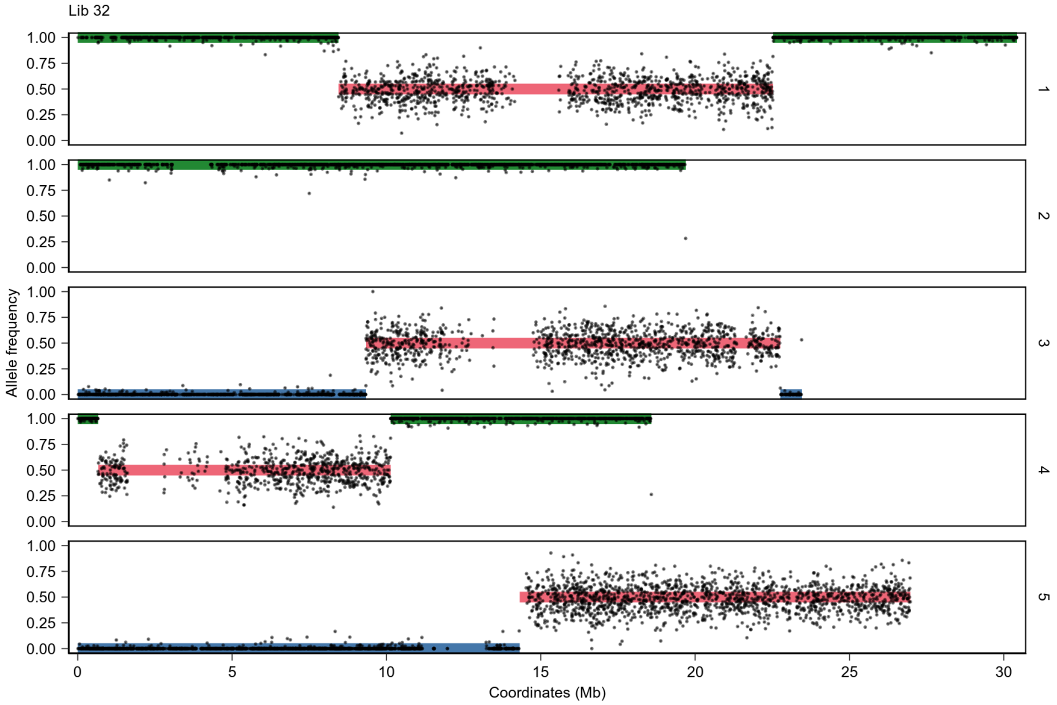

Lib 32
1.00
0.75
0.50
0.25
0.00
1
2
3
4
5
Allele frequency
0
5
10
15
20
25
30
Coordinates (Mb)

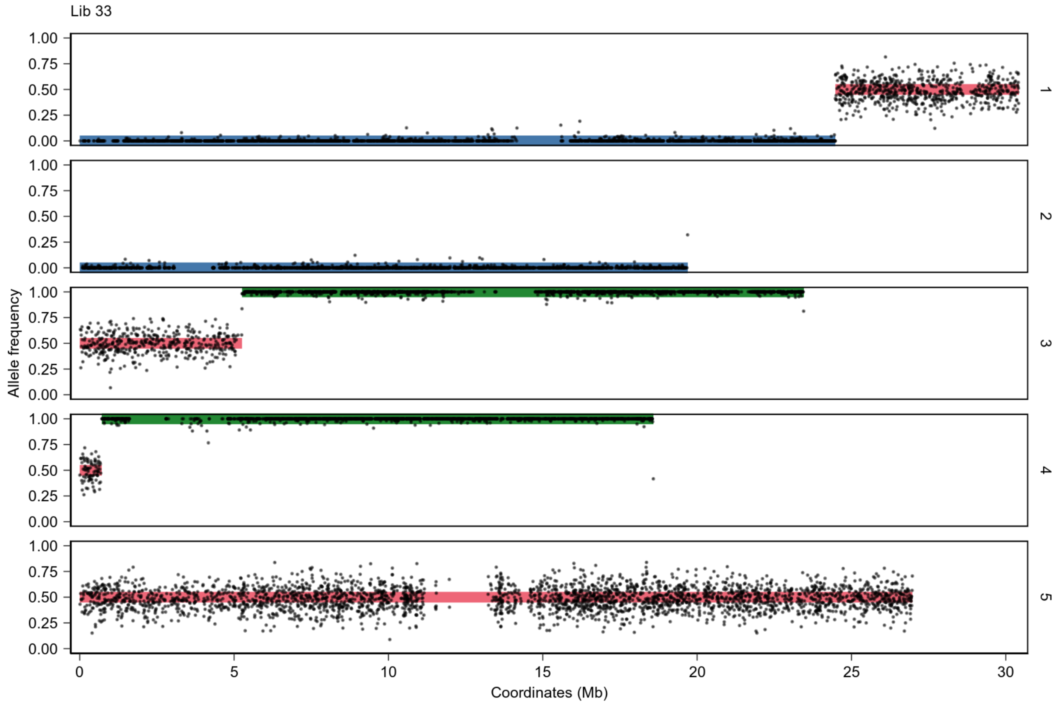

Lib 33
Allele frequency
1.00
0.75
0.50
0.25
0.00
1
2
3
4
5
0
5
10
15
20
25
30
Coordinates (Mb)

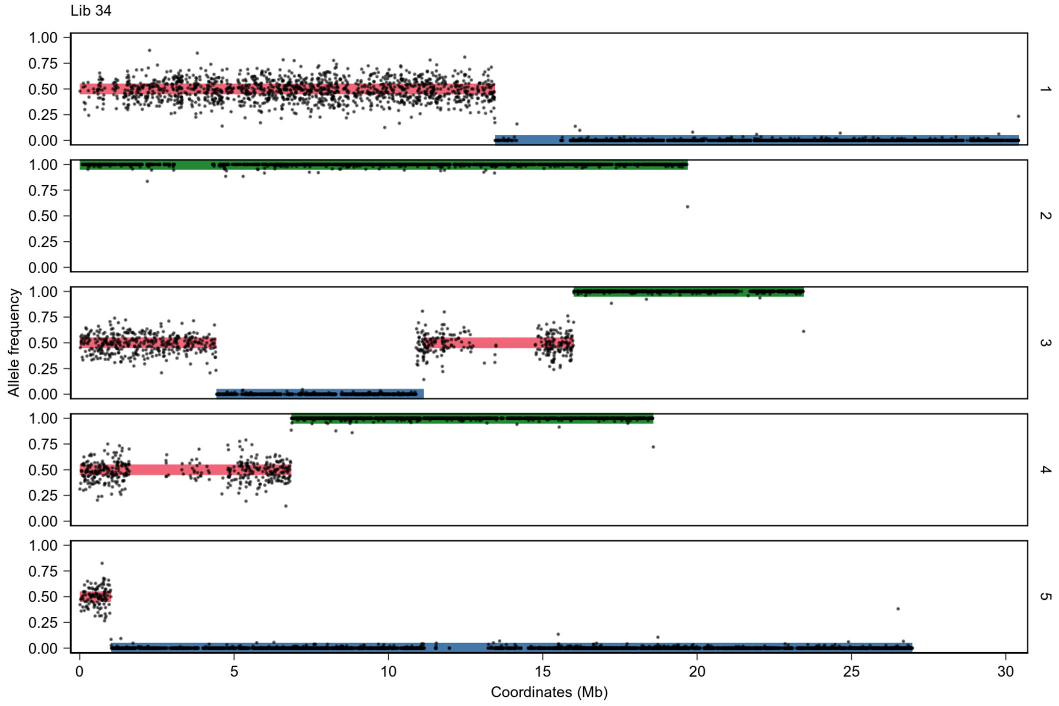

Lib 34
Allele frequency
1.00
0.75
0.50
0.25
0.00
1
2
3
4
5
0
5
10
15
20
25
30
Coordinates (Mb)

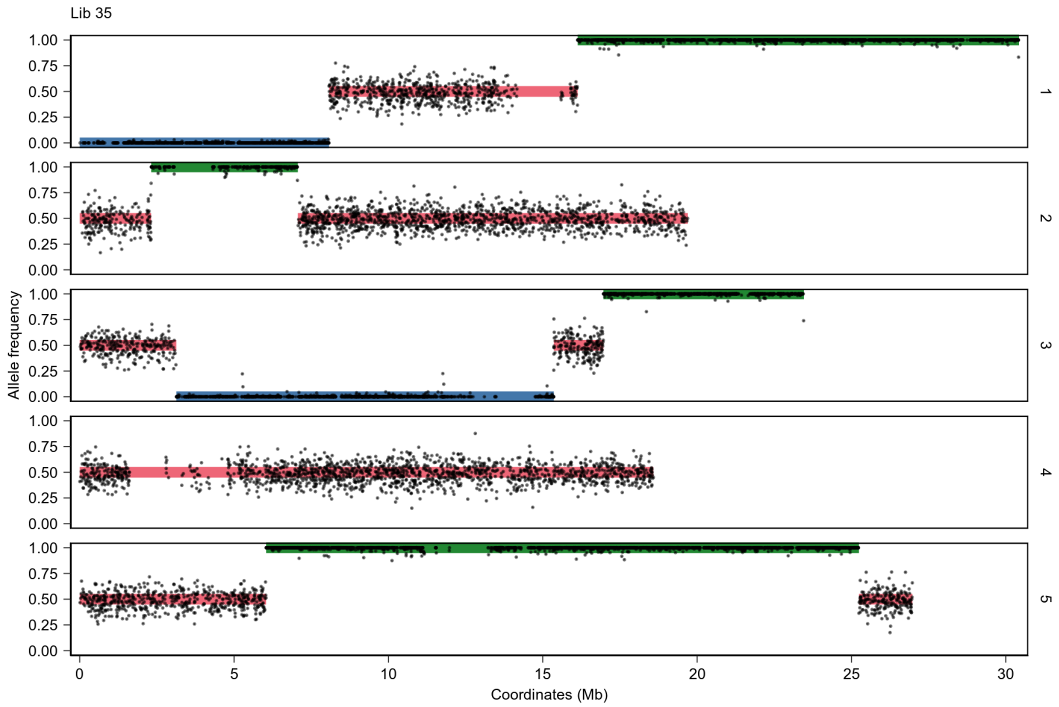
Lib 35
Allele frequency
Coordinates (Mb)
1
2
3
4
5

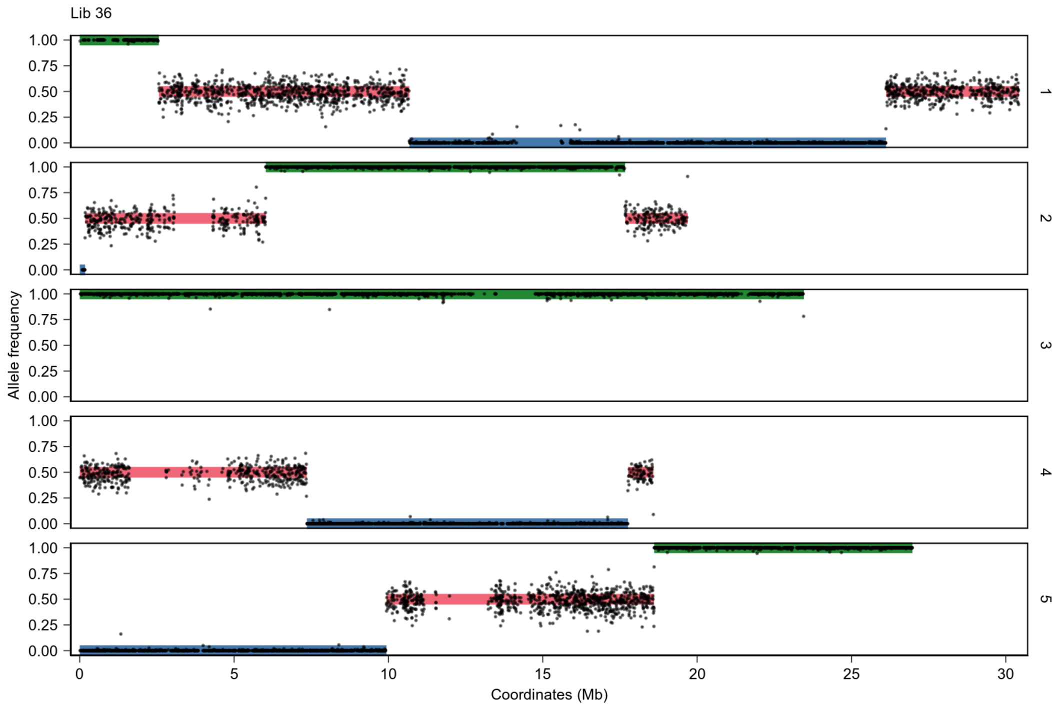
Lib 36
Allele frequency
Coordinates (Mb)
1
2
3
4
5

Lib 37

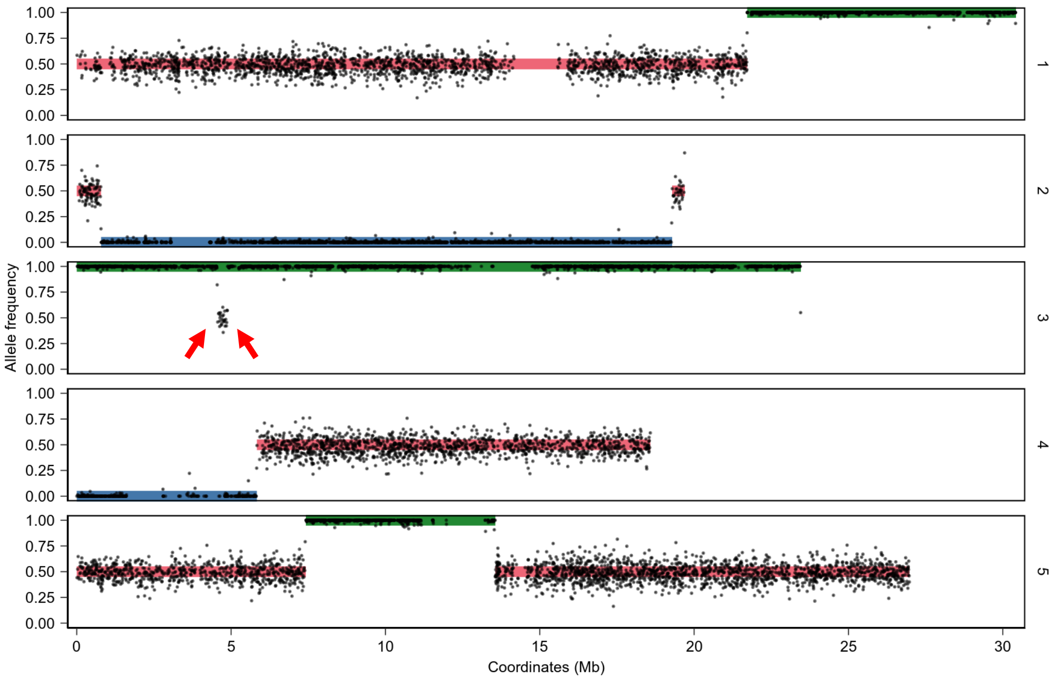

Allele frequency
1.00
0.75
0.50
0.25
0.00
1
2
3
4
5
0
5
10
15
20
25
30
Coordinates (Mb)

Lib 38

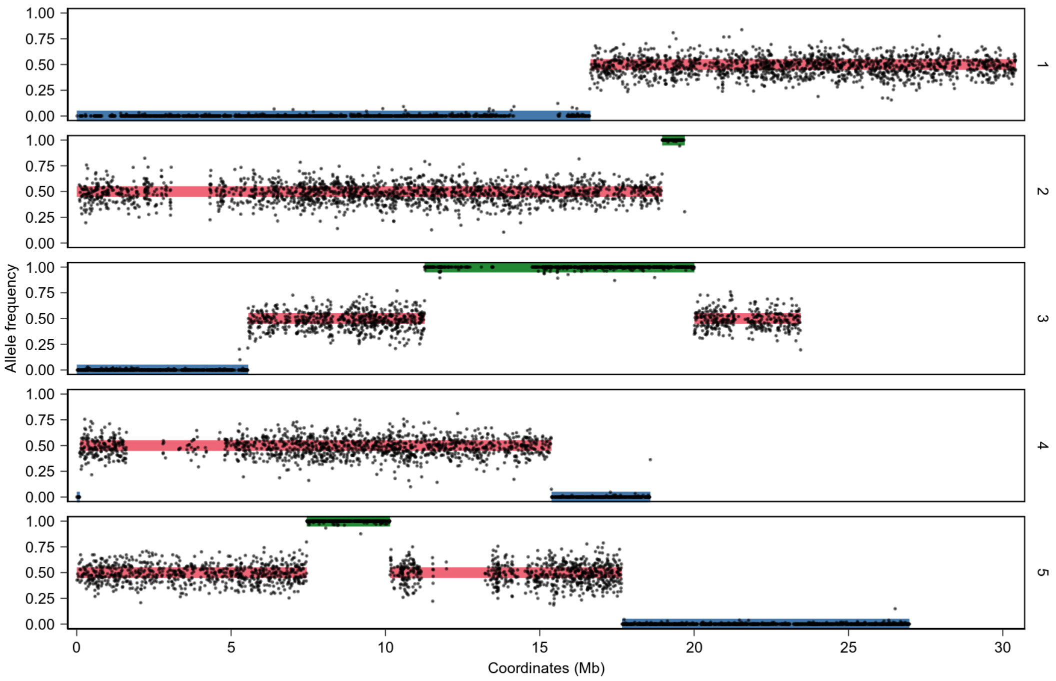

Allele frequency
1.00
0.75
0.50
0.25
0.00
1
2
3
4
5
0
5
10
15
20
25
30
Coordinates (Mb)

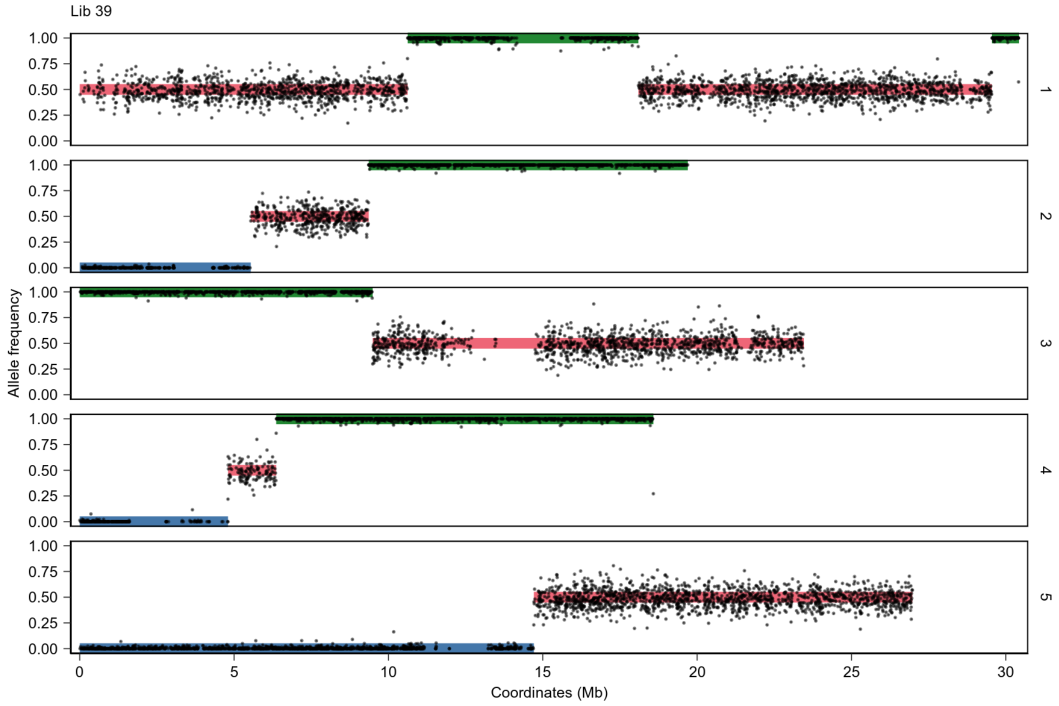
Lib 39
Allele frequency
Coordinates (Mb)
1
2
3
4
5

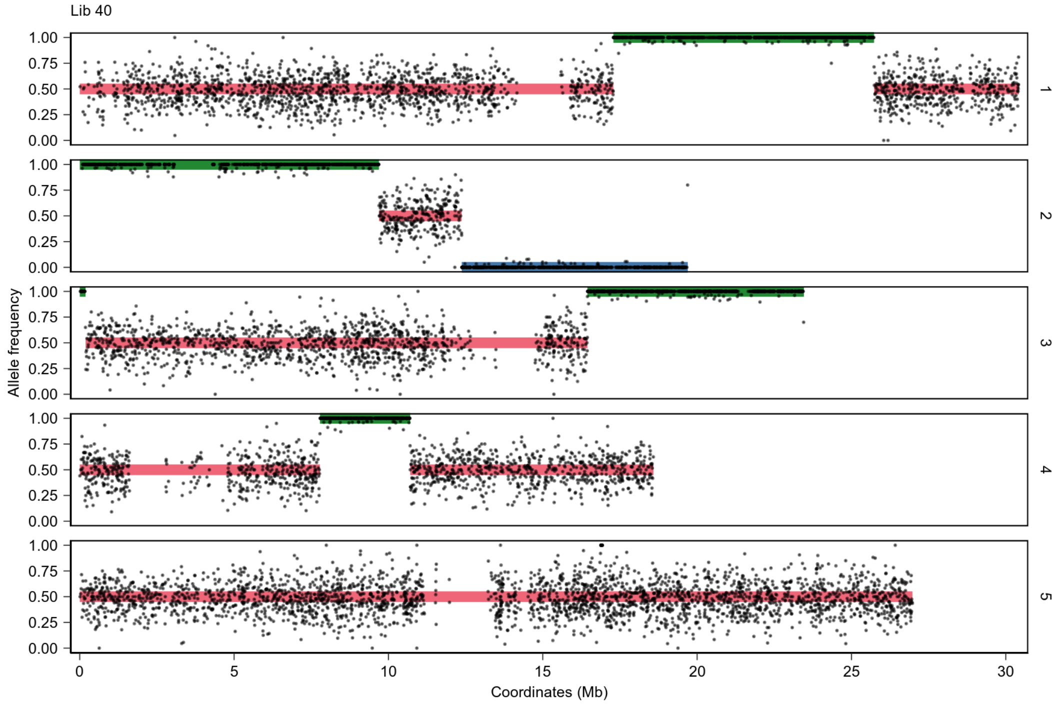
Lib 40
Allele frequency
Coordinates (Mb)
1
2
3
4
5

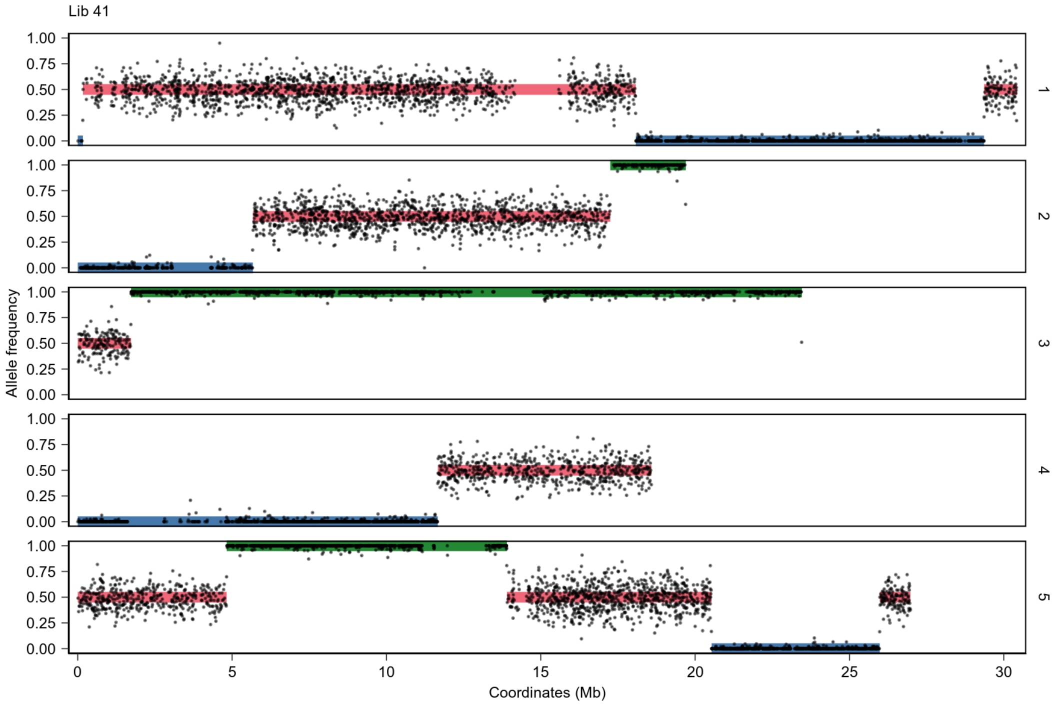
Lib 41
1.00
0.75
0.50
0.25
0.00
Allele frequency
1
2
3
4
5
0
5
10
15
20
25
30
Coordinates (Mb)

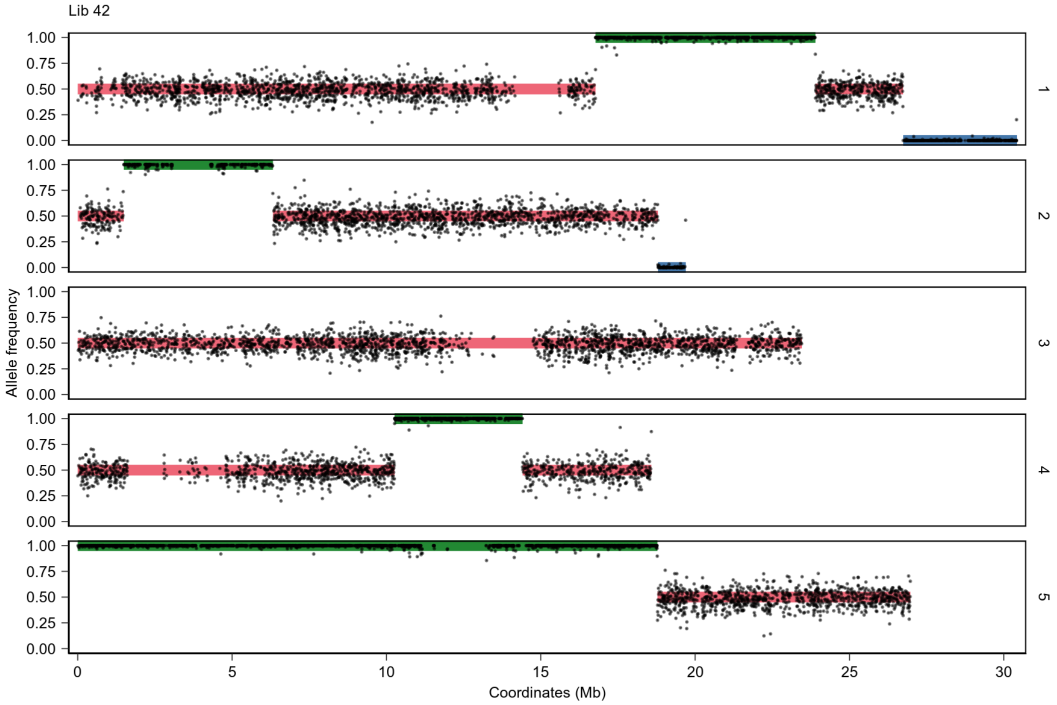
Lib 42
1.00
0.75
0.50
0.25
0.00
Allele frequency
1
2
3
4
5
0
5
10
15
20
25
30
Coordinates (Mb)

Lib 43

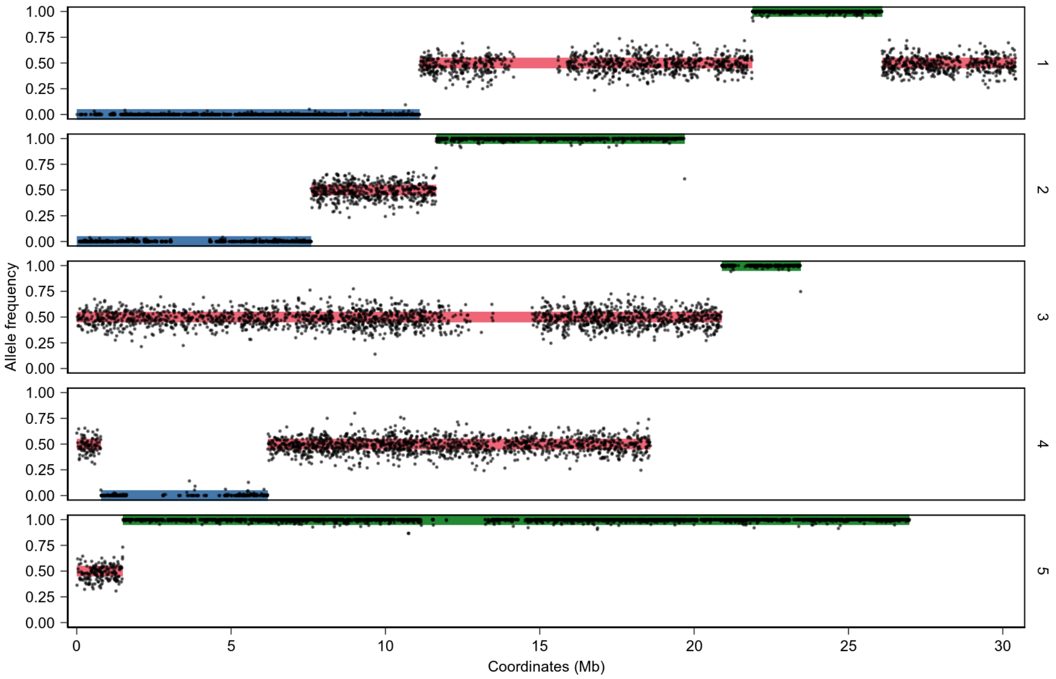

1.00
0.75
0.50
0.25
0.00
1
2
3
4
5
Allele frequency
0
5
10
15
20
25
30
Coordinates (Mb)

Lib 44

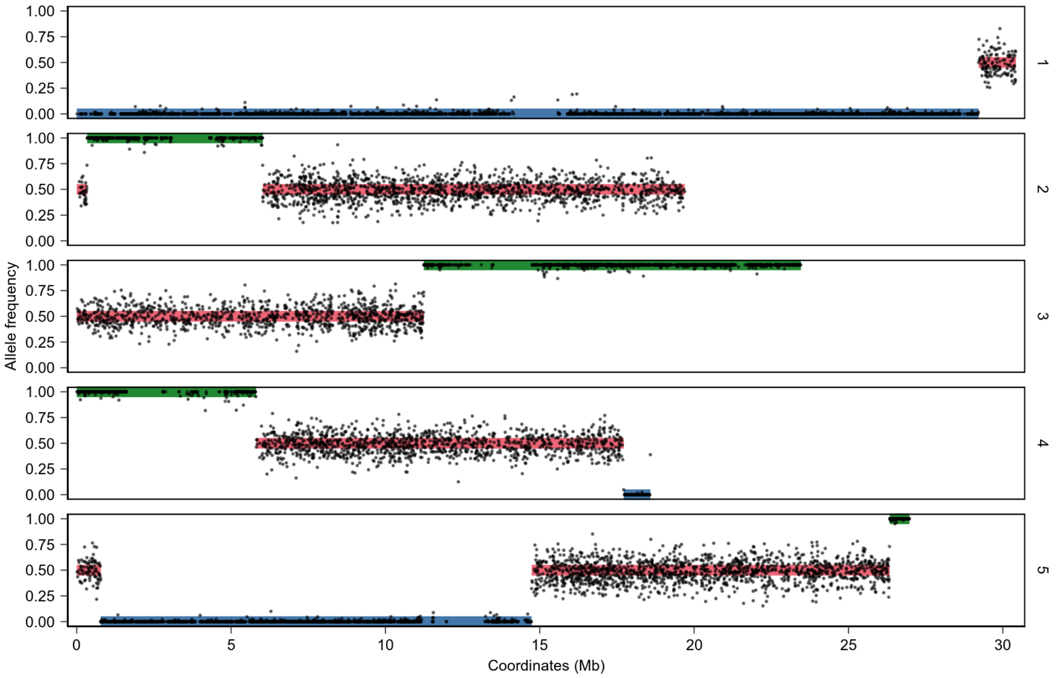

1.00
0.75
0.50
0.25
0.00
1
2
3
4
5
Allele frequency
0
5
10
15
20
25
30
Coordinates (Mb)

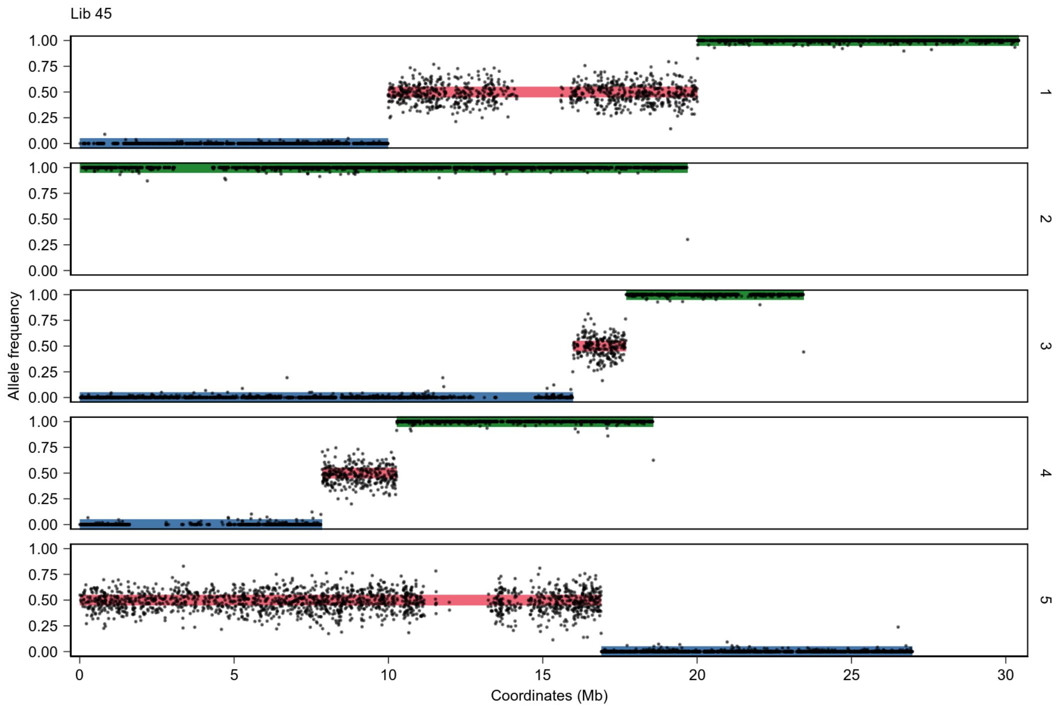
Lib 45
Allele frequency
Coordinates (Mb)
1
2
3
4
5
1.00
0.75
0.50
0.25
0.00
0
5
10
15
20
25
30

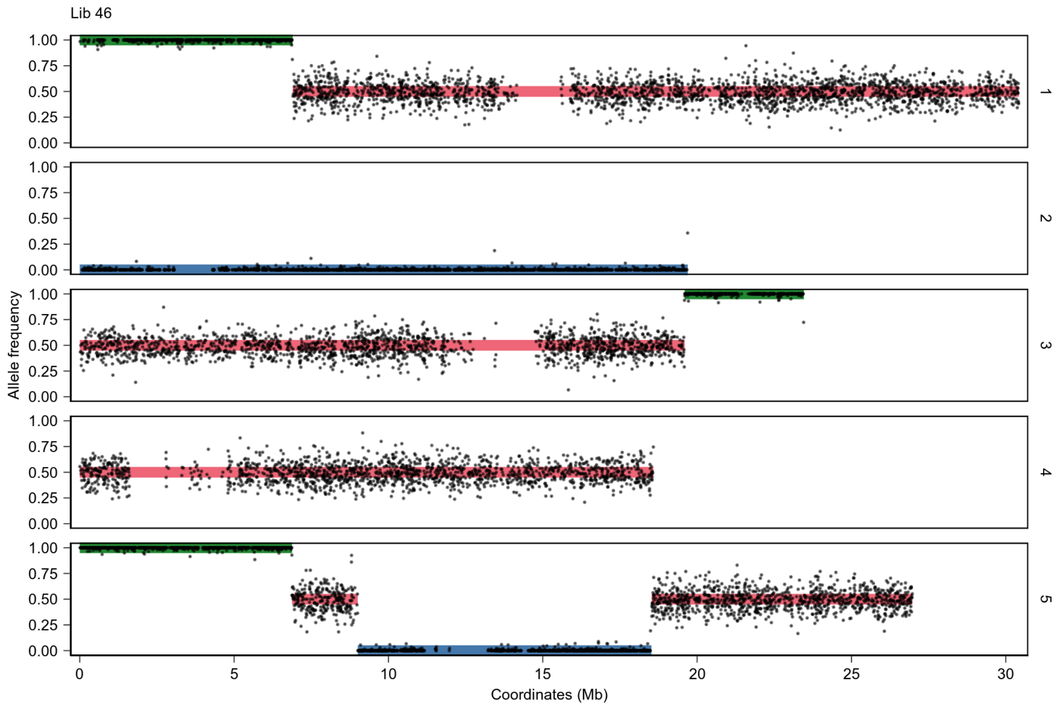
Lib 46
Allele frequency
Coordinates (Mb)
1
2
3
4
5
1.00
0.75
0.50
0.25
0.00
0
5
10
15
20
25
30

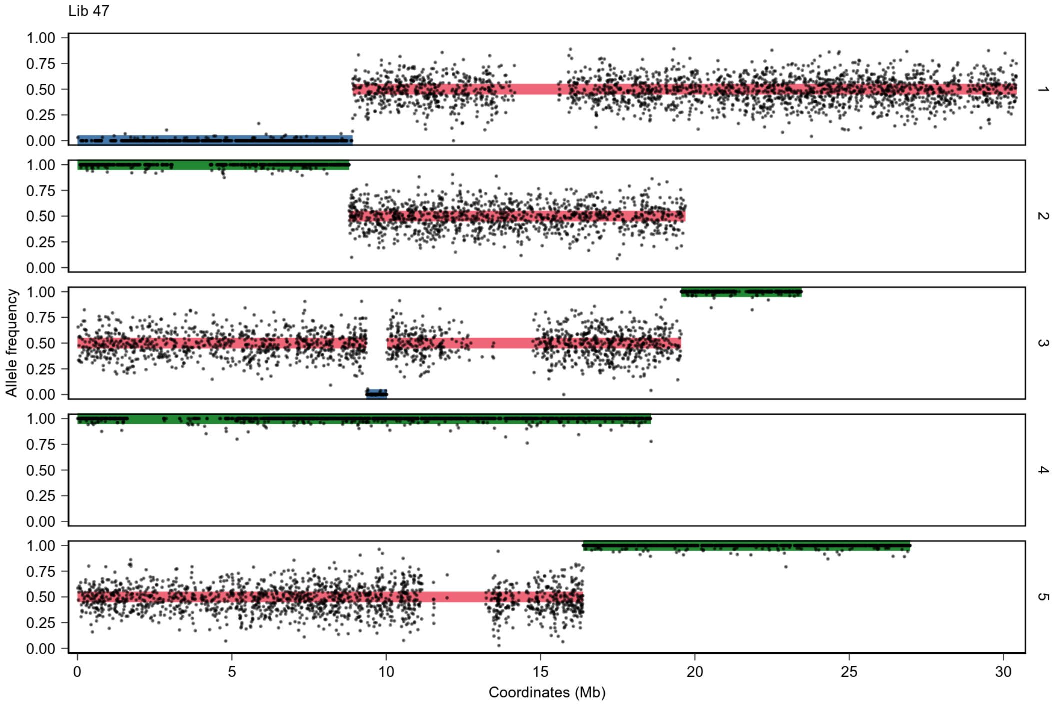

Lib 47
Allele frequency
Coordinates (Mb)
1.00
0.75
0.50
0.25
0.00
0
5
10
15
20
25
30
1
2
3
4
5

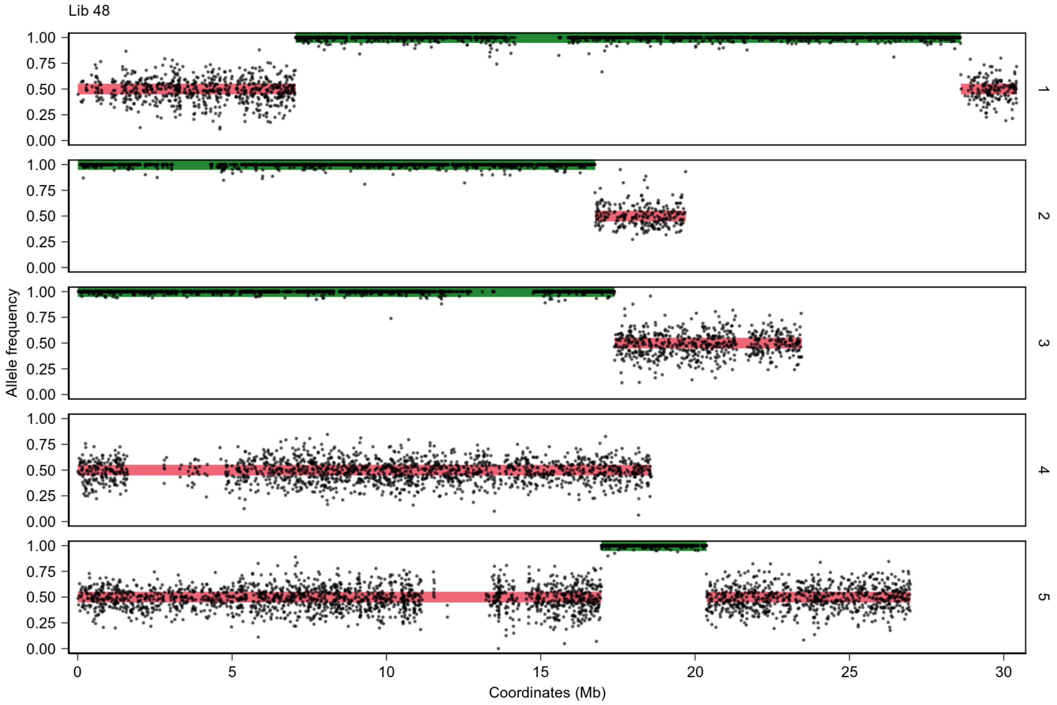

Lib 48
Allele frequency
Coordinates (Mb)
1.00
0.75
0.50
0.25
0.00
0
5
10
15
20
25
30
1
2
3
4
5

Lib 53

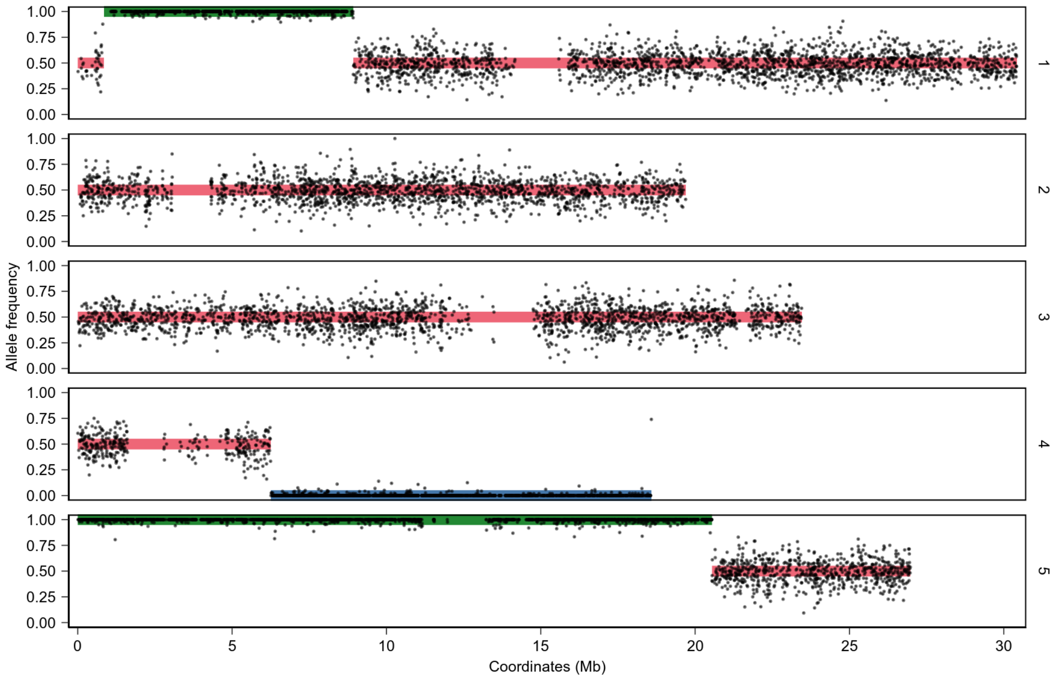

1.00
0.75
0.50
0.25
0.00
Allele frequency
1
2
3
4
5
0
5
10
15
20
25
30
Coordinates (Mb)

Lib 54

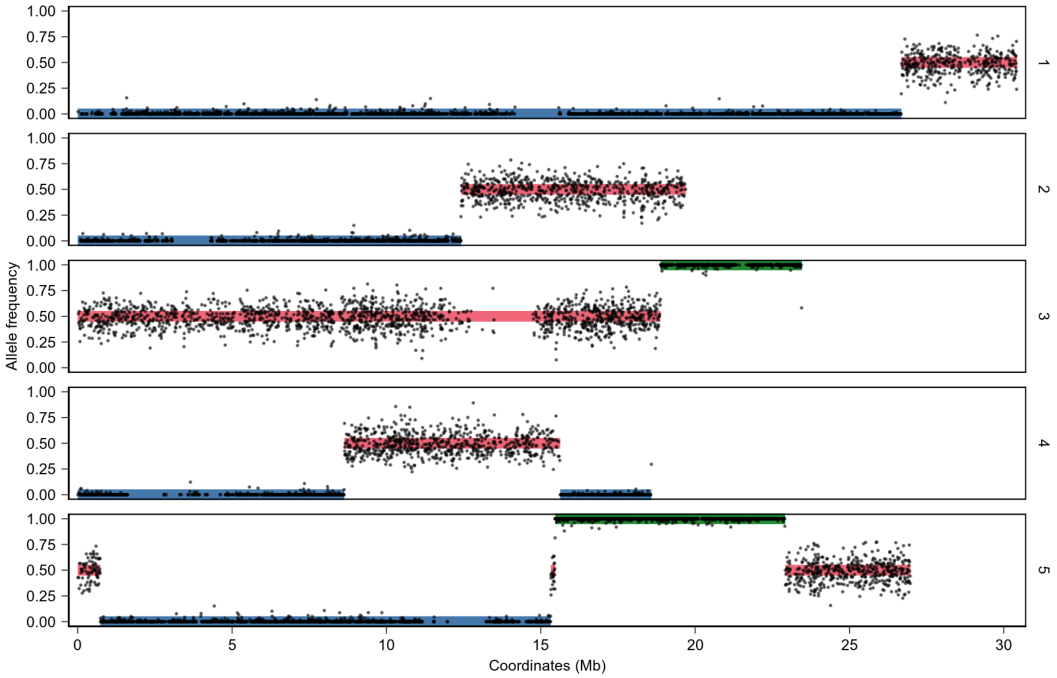

1.00
0.75
0.50
0.25
0.00
Allele frequency
1
2
3
4
5
0
5
10
15
20
25
30
Coordinates (Mb)

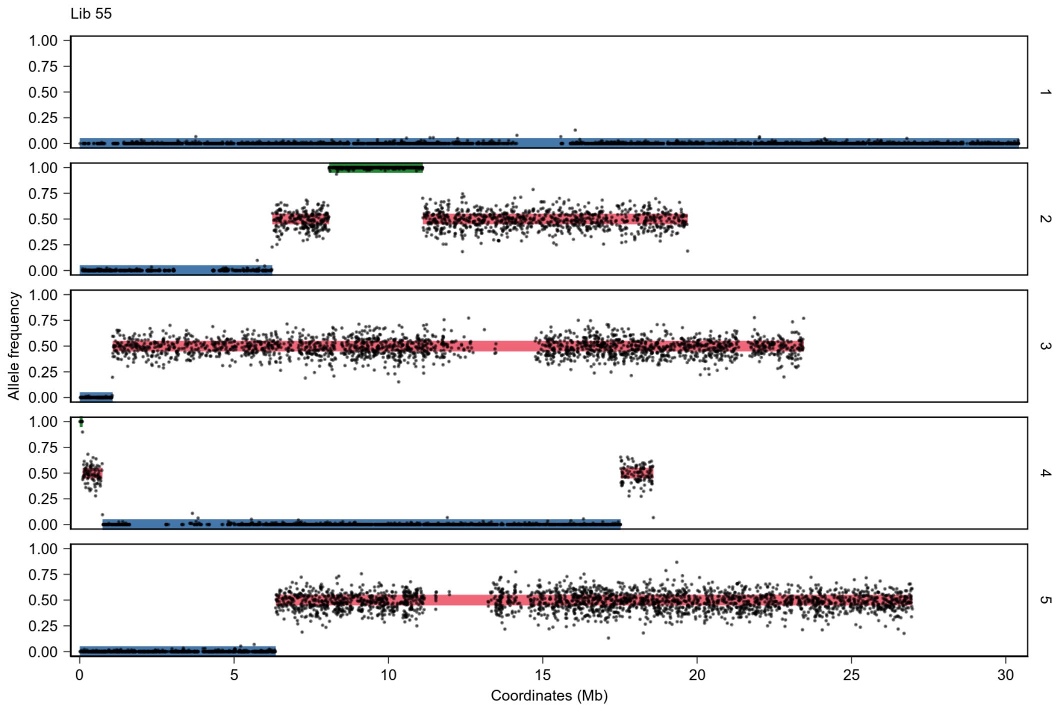
Lib 55
1.00
0.75
0.50
0.25
0.00
Allele frequency
1
2
3
4
5
0
5
10
15
20
25
30
Coordinates (Mb)

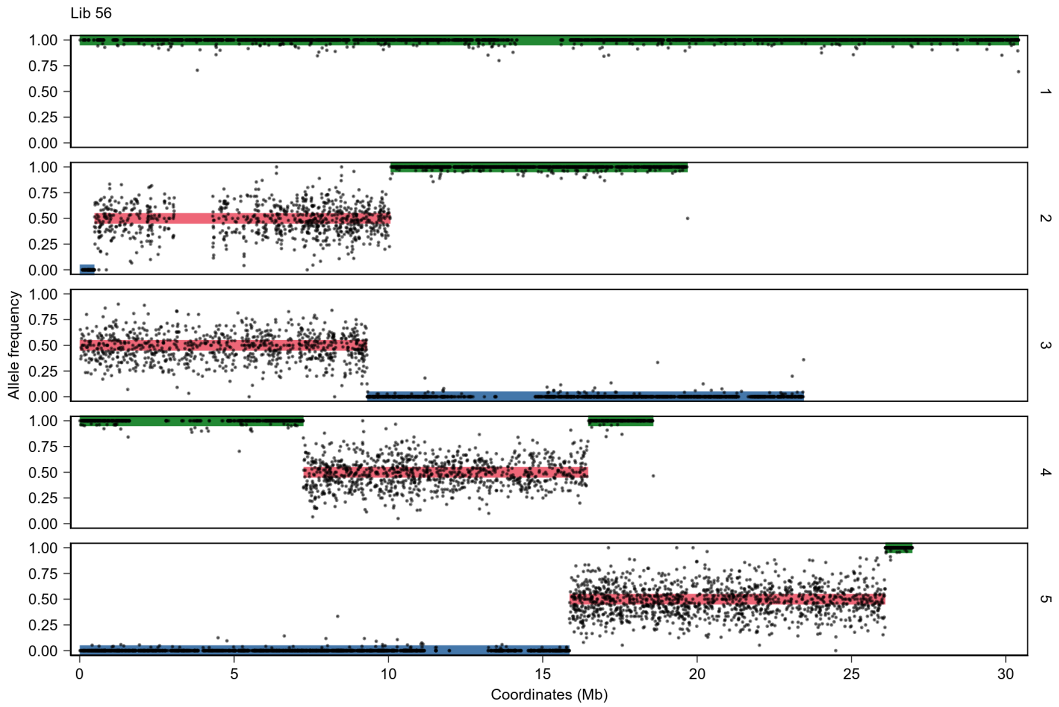
Lib 56
1.00
0.75
0.50
0.25
0.00
Allele frequency
1
2
3
4
5
0
5
10
15
20
25
30
Coordinates (Mb)

4 F_2 individuals

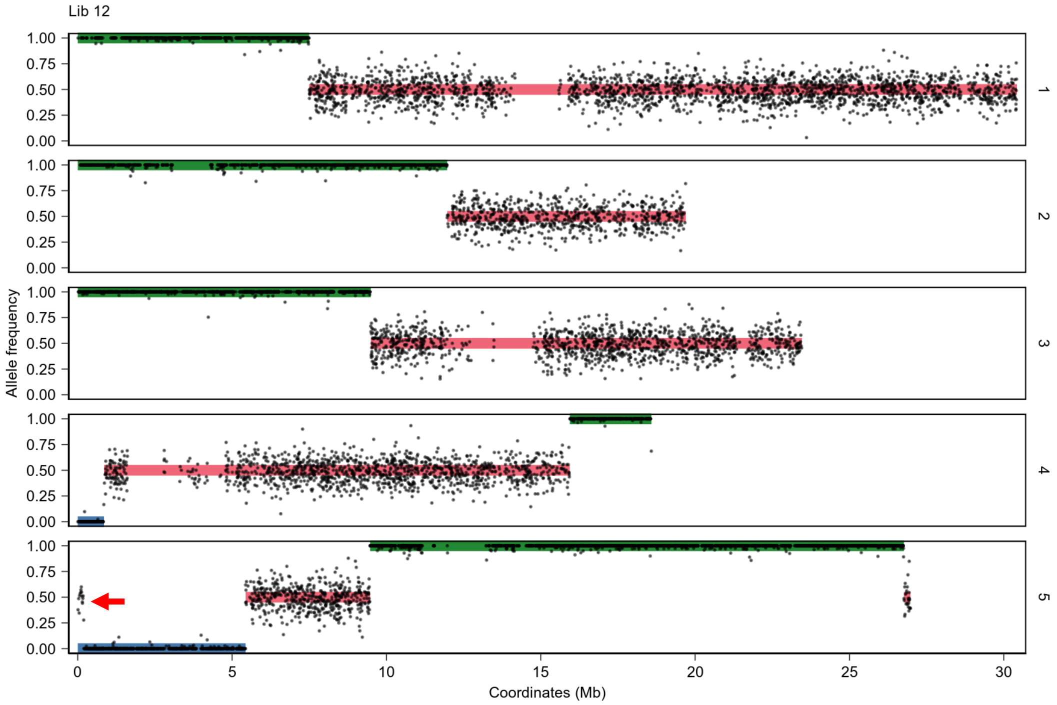
Lib 12
Allele frequency
Coordinates (Mb)
1.00
0.75
0.50
0.25
0.00
0
5
10
15
20
25
30
1
2
3
4
5

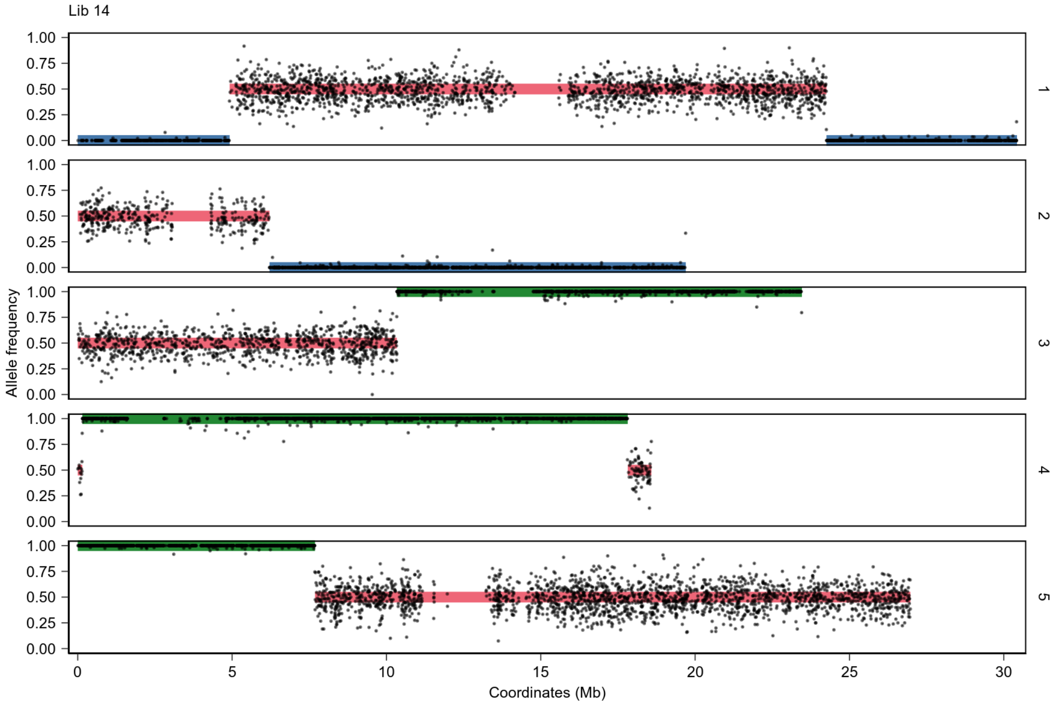
Lib 14
Allele frequency
Coordinates (Mb)
1.00
0.75
0.50
0.25
0.00
0
5
10
15
20
25
30
1
2
3
4
5

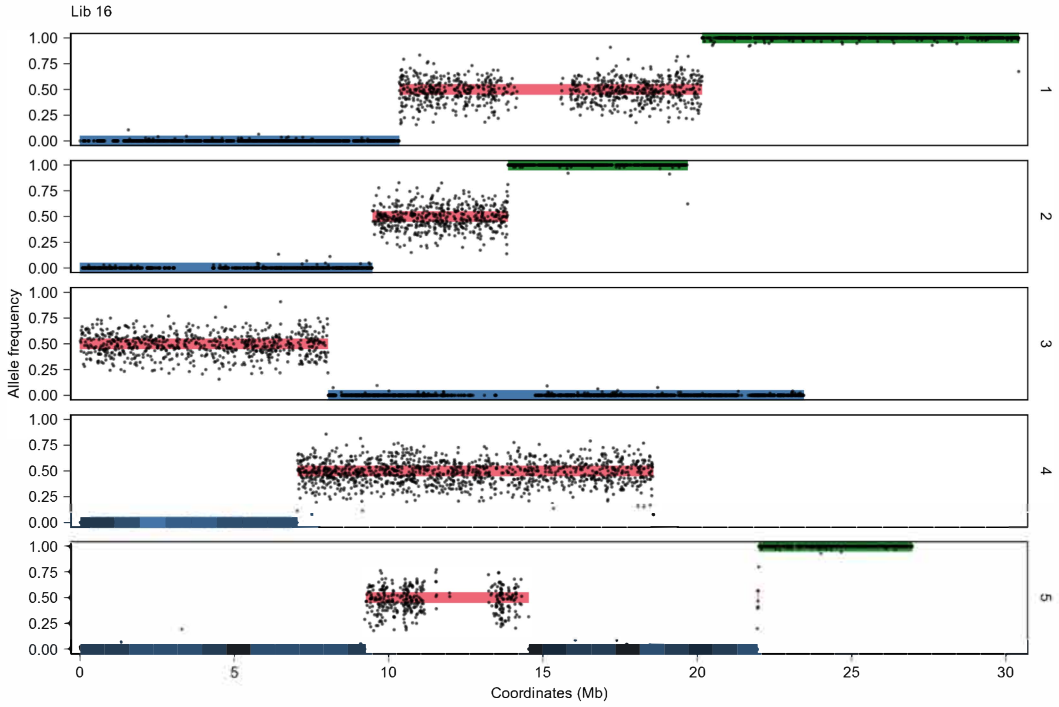

Lib 16
Allele frequency
1.00
0.75
0.50
0.25
0.00
1
2
3
4
5
0
5
10
15
20
25
30
Coordinates (Mb)

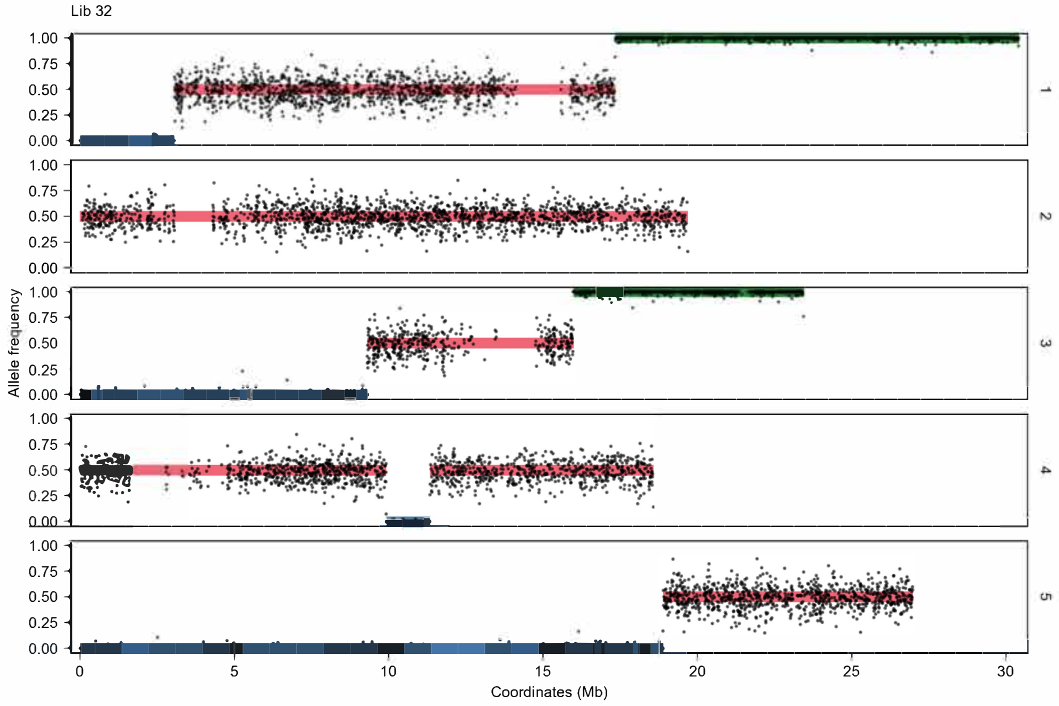

Lib 32
Allele frequency
1.00
0.75
0.50
0.25
0.00
1
2
3
4
5
0
5
10
15
20
25
30
Coordinates (Mb)

Dataset S3. Screenshots of false positives.

False positive case #1: Chimeric read with error cluster

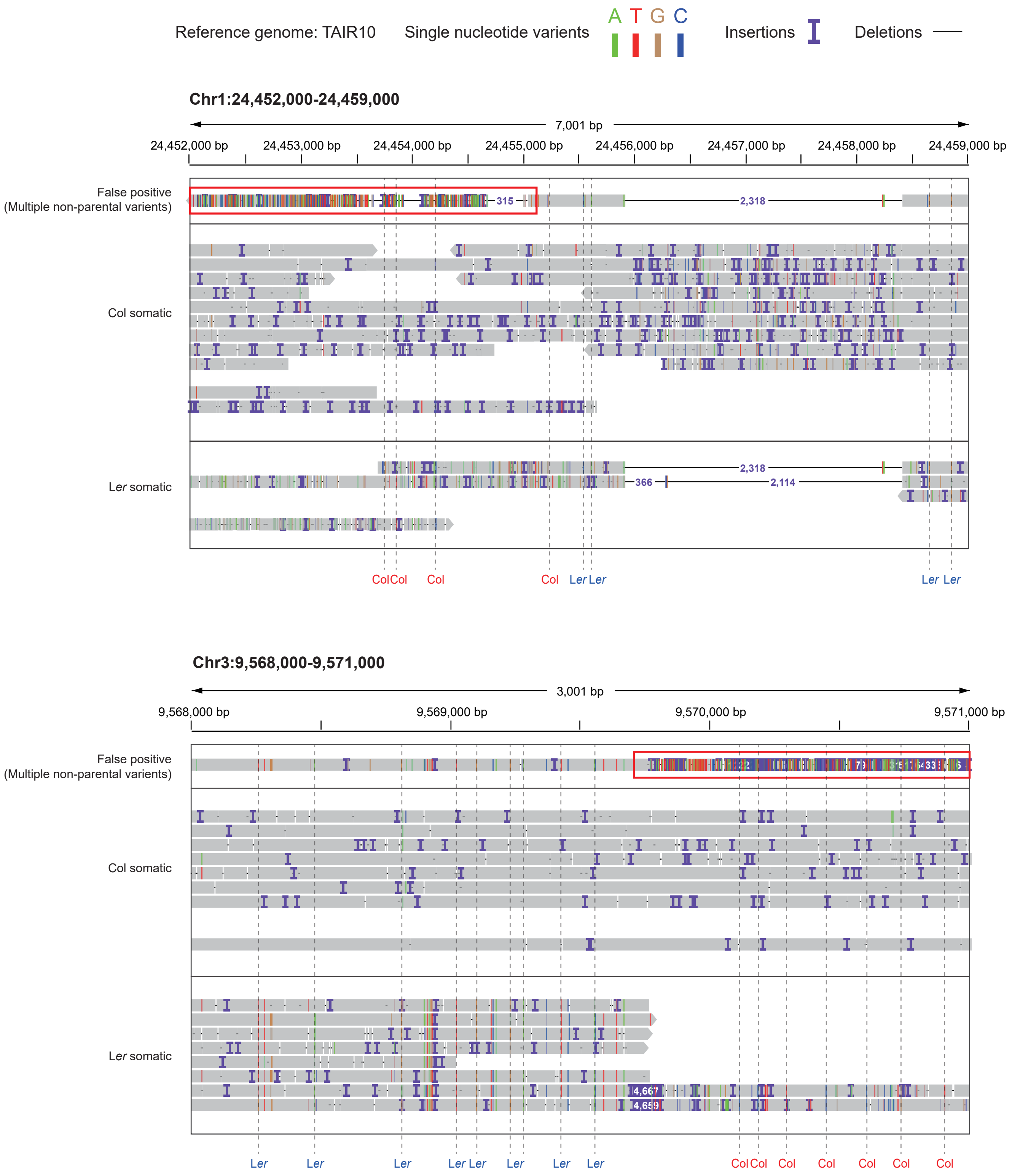

False positive case #2: Substitution error

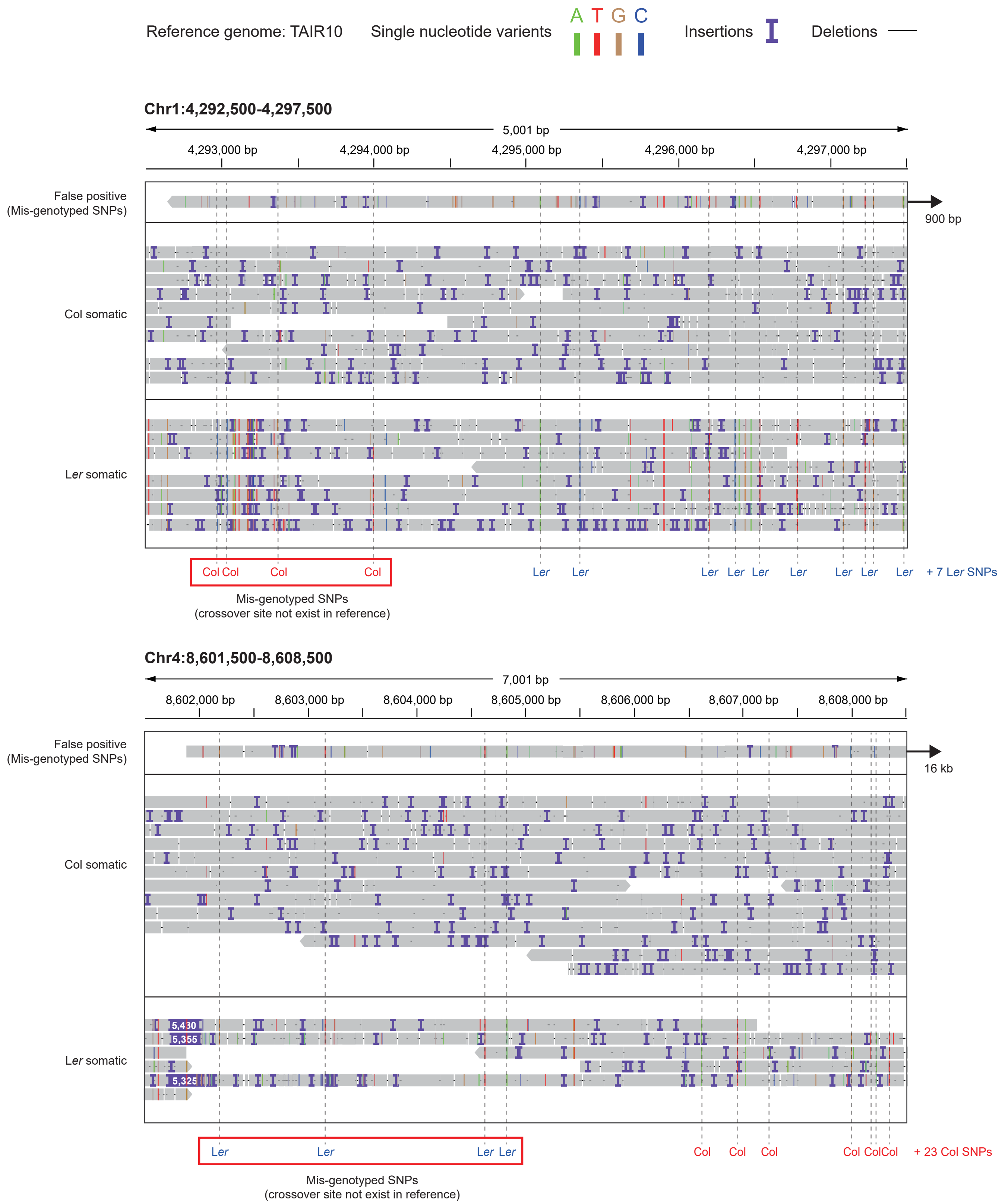

False positive case #3: Mitotic recombination

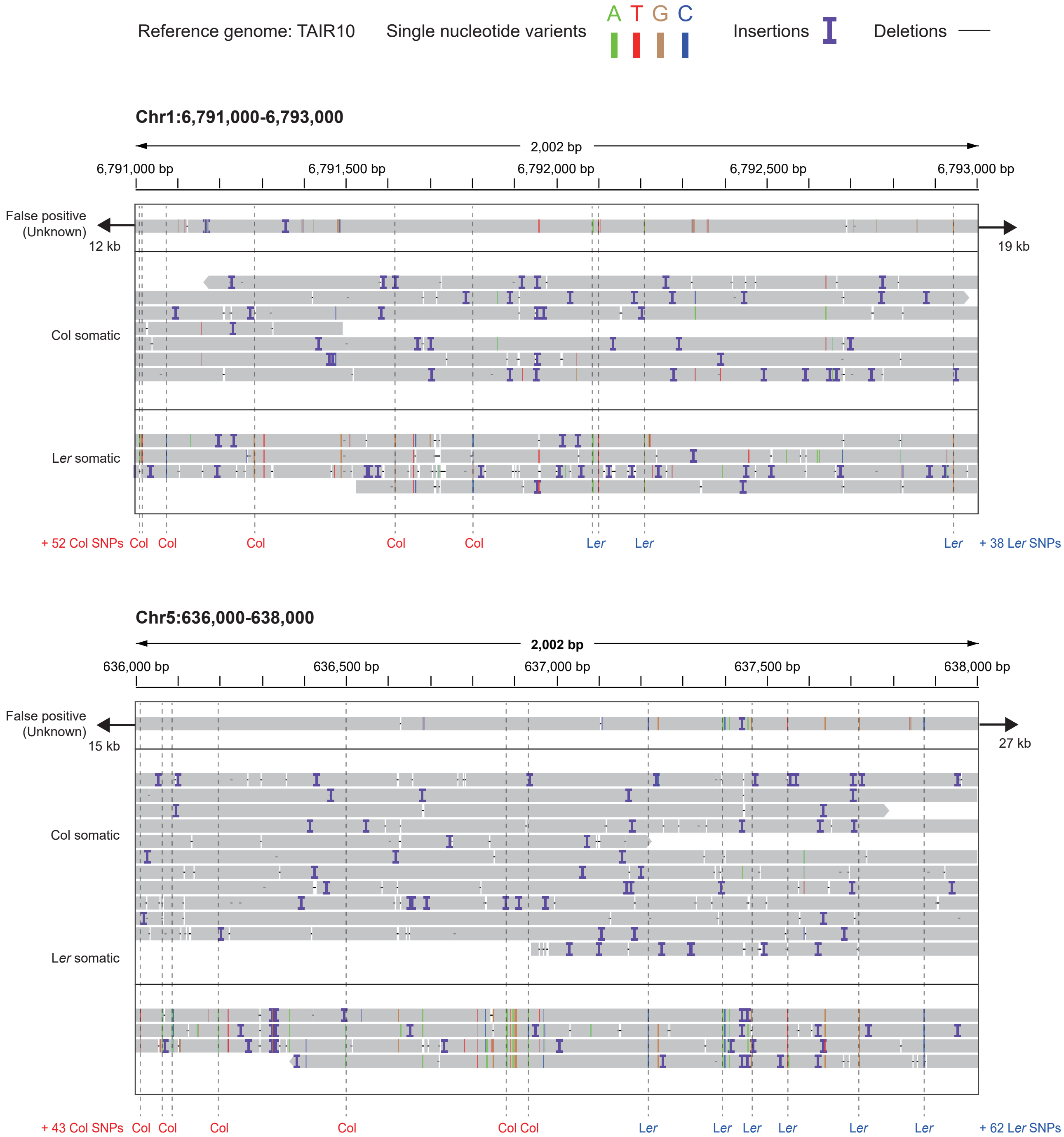

False positive case #4: Chimeric reads with large indel

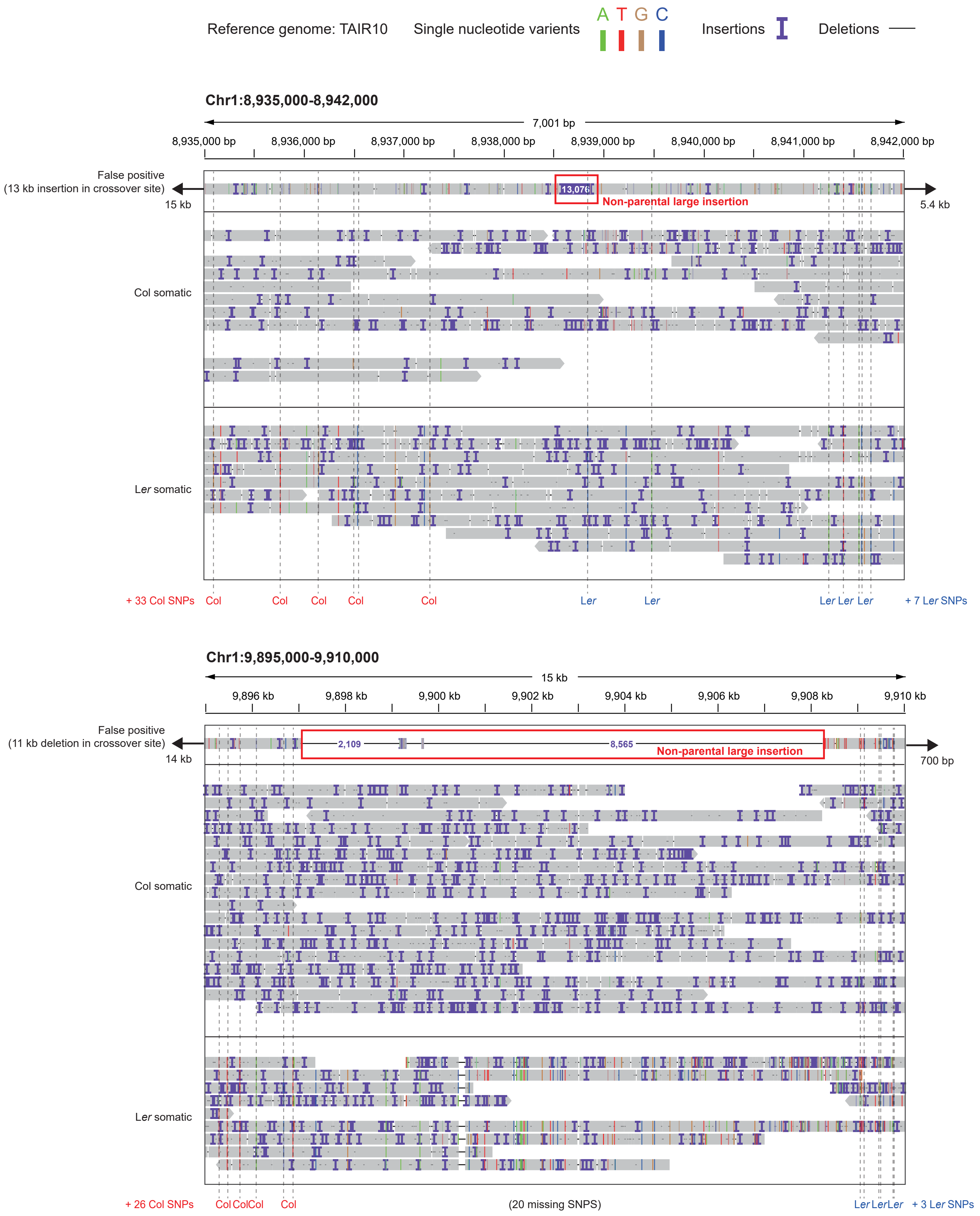

Reference

Kim H, Kim J, Son N, Kuo P, Morgan C, Chambon A, Byun D, Park J, Lee Y, Park YM, *et al.* 2024. Control of meiotic crossover interference by a proteolytic chaperone network. *Nature Plants* **10**: 453–468.

Kim J, Park J, Kim H, Son N, Kim E, Kim JJ, Byun D, Lee Y, Park YM, Nageswaran DC, *et al.* 2022. Arabidopsis HEAT SHOCK FACTOR BINDING PROTEIN is required to limit meiotic crossovers and HEI10 transcription. *The EMBO Journal* **41**: e109958.

Nageswaran DC, Kim J, Lambing C, Kim J, Park J, Kim E-J, Cho HS, Kim H, Byun D, Park YM, *et al.* 2021. HIGH CROSSOVER RATE1 encodes PROTEIN PHOSPHATASE X1 and restricts meiotic crossovers in Arabidopsis. *Nature Plants* **7**: 452–467.

Rowan BA, Heavens D, Feuerborn TR, Tock AJ, Henderson IR, Weigel D. **2019**. An Ultra High-Density Arabidopsis thaliana Crossover Map That Refines the Influences of Structural Variation and Epigenetic Features. *Genetics* **213**: 771–787.
